# Identification of key pharmacological components and targets for Aidi injection in the treatment of pancreatic cancer by UPLC-MS, network pharmacology, and in vivo experiments

**DOI:** 10.1186/s13020-023-00710-2

**Published:** 2023-01-14

**Authors:** Haojia Wang, Zhishan Wu, Xiaotian Fan, Chao Wu, Shan Lu, Libo Geng, Antony Stalin, Yingli Zhu, Fanqin Zhang, Jiaqi Huang, Pengyun Liu, Huiying Li, Leiming You, Jiarui Wu

**Affiliations:** 1 Department of Clinical Chinese Pharmacy, School of Chinese Materia Medica, Beijing University of Chinese Medicine, Beijing, 100102 China; 2 School of Chinese Medicine, Bozhou University, Bozhou, 236800 China; 3 Guizhou Yibai Pharmaceutical Co. Ltd, Guiyang, 550008 Guizhou China; 4 Institute of Fundamental and Frontier Sciences, University of Electronic Science and Technology of China, Chengdu, 610054 China; 5 School of Biology, Beijing Forestry University, Beijing, 100091 China; 6 School of Life Sciences, Beijing University of Chinese Medicine, Beijing, 100102 China

**Keywords:** Pancreatic cancer, Aidi injection, Network pharmacology, Molecular docking, Pharmacodynamic evaluation

## Abstract

**Background:**

Pancreatic cancer is one of the most lethal cancers worldwide. Aidi injection (ADI) is a representative antitumor medication based on Chinese herbal injection, but its antitumor mechanisms are still poorly understood.

**Materials and methods:**

In this work, the subcutaneous xenograft model of human pancreatic cancer cell line Panc-1 was established in nude mice to investigate the anticancer effect of ADI in *vivo*. We then determined the components of ADI using ultra-performance liquid chromatography-tandem mass spectrometry (UPLC-MS) and explored the possible molecular mechanisms against pancreatic cancer using network pharmacology.

**Results:**

In *vivo* experiments, the volume, weight, and degree of histological abnormalities of implanted tumors were significantly lower in the medium and high concentration ADI injection groups than in the control group. Network pharmacology analysis identified four active components of ADI and seven key targets, TNF, VEGFA, HSP90AA1, MAPK14, CASP3, P53 and JUN. Molecular docking also revealed high affinity between the active components and the target proteins, including Astragaloside IV to P53 and VEGFA, Ginsenoside Rb1 to CASP3 and Formononetin to JUN.

**Conclusion:**

ADI could reduce the growth rate of tumor tissue and alleviate the structural abnormalities in tumor tissue. ADI is predicted to act on VEGFA, P53, CASP3, and JUN in ADI-mediated treatment of pancreatic cancer.

**Supplementary Information:**

The online version contains supplementary material available at 10.1186/s13020-023-00710-2.

## Introduction

Pancreatic cancer (PC) is one of the most lethal cancers worldwide and the fourth leading cause of cancer-related death in the United States [1]. According to the American Cancer Society’s global cancer statistics for 2020, there were approximately 495, 000 new cases of PC and 466, 000 deaths [2]. This indicates that PC has a poor prognosis and high mortality. With the improvement of living standard in the Chinese population, the number of new PC cases increased from 95,000 in 2015 to 125,000 in 2020, and the number of deaths has increased from 85,000 to 122,000. The mortality rate of PC remains high and is the sixth leading cause of cancer death in China [3]. According to the NCCN Clinical Practice Guidelines for Pancreatic Cancer, radical resection is currently the most effective treatment for PC. However, as the disease progresses and worsens, patients seek medical attention with nonspecific symptoms such as abdominal pain, fatigue, jaundice, and weight loss. At this point, 80–85 percent of diagnosed patients have lost the chance of surgical cure [4, 5]. Chemotherapy is still the most commonly chosen clinical treatment in China. However, it has immense limitations, such as low response rate to treatment regimens, easy development of drug resistance, and serious adverse effects due to toxicity [6–8]. Traditional Chinese medicine (TCM) has a unique position in the treatment of tumors. By regulating the balance of local lesions and systemic functions, it ultimately maintains patients’ quality of life and achieves the goal of “survival with tumors” [9]. Aidi injection (ADI) is a modernized preparation of the extract of traditional Chinese medicines including *Panax ginseng* C.A.Mey. (Ginseng Radix et Rhizoma), *Astragalus mongholicus* Bunge (Astragali Radix), *Eleutherococcus senticosus* (Rupr. & Maxim.) Maxim (Acanthopanacis Senticosi Radix Et Rhizoma Seu Caulis) and *Mylabris phalerata* Pallas (Mylabris). Its indications include the elimination of heat and detoxification, dissipating blood stasis and removing knots. It is a representative antitumor medication of Chinese herbal injections and is mainly used for primary liver cancer, lung cancer, rectal cancer, etc. [10, 11], but its antitumor mechanisms are still poorly understood.

The network pharmacology method is based on the overall concept of TCM. By constructing “drug-gene-disease” modules that are interconnected and performing network topology analysis, the key targets of a drug in treating diseases can be predicted. The network pharmacology method is an important way to transform TCM from empirical to evidence-based medicine [12]. Therefore, in this work, we established a xenograft model using human pancreatic cancer cells in nude mice to investigate the anticancer effect of ADI in *vivo*. To decipher the active components and potential targets of ADI in the treatment of PC, we not only directly determined the possible active components of ADI by the method of ultra-performance liquid chromatography-tandem mass spectrometry (UPLC-MS), but also predicted the potential targets of the obtained active components by network pharmacology and finally merged them with the reported disease targets of PC to obtain more confident targets of ADI in the treatment of PC. We also performed molecular docking to verify further binding between the active components and their potential targets. These results may provide new insights into the mechanism of action of ADI in the treatment of PC.

## Materials and methods

### Experimental animals

Female specific pathogen-free (SPF) BALB/c nude mice (age 4–6 weeks) were purchased from the Beijing SiPeiFu Biotechnology Co., Ltd., under the Laboratory Animal Production License No. SCXK 2019-0010. These mice were maintained under SPF conditions in the Animal house with Laboratory Animal License No. SYXK 2020-0050. The use of the animals and the experimental protocols were approved by the Animal Experimentation Ethics Committee of Beijing University of Chinese medicine (Ethical Code: BUCM-4-2021032003-1091).

### Cell culture

The human pancreatic carcinoma cell line Panc-1 was purchased from Procell Biotechnology Co., Ltd. (Wuhan, China). Cell culture was performed in Dulbecco’s modified Eagle’s medium (DMEM, Gibco, USA) supplemented with 10% fetal bovine serum (FBS, Corning, USA), and 1% penicillin/ streptomycin (Gibco, USA) in a humidified incubator at 37 °C and 5% CO_2_. ADI is provided by Guizhou Yibai Pharmaceutical Co., Ltd. (Guizhou, China). Information about the Aidi injection is shown in Additional file 1: Table S1.

### Animal grouping and model establishment

After 1 week of adaptive feeding of female BALB/c nude mice, Panc-1 cells (2 × 10^6^) were injected subcutaneously into the right axilla of the mice. Mice were randomly assigned to the following 4 groups (n = 5 per group) using a table of random numbers: Control, Low-Medium–High dose of ADI (ADI-L, ADI-M, ADI-H) groups. ADI was administered in equivalent doses converted from upper clinical limits in human, with doses of 7.8 mL/kg (ADI-L), 15.6 mL/kg (ADI-M) and 31.2 mL/kg (ADI-H). The low, medium and high doses of ADI corresponded to 0.6, 1.2, and 2.4 times, respectively, the equivalent dose after conversion. When the tumors had grown to about 50–100 mm^3^, the mice were injected with ADI intraperitoneally for 21 consecutive days.

The tumor size and body weight of the mice were measured every 3 days, and tumor volume was calculated via 1/2 × L^2^ × W, where L represents the biggest diameter (mm) and W represents the smallest diameter (mm). On day 21, the mice were weighed and the subcutaneous tumor nodules were surgically removed and photographed. The formula for tumor inhibition rate was as follows: (1 − the tumor weight of the ADI group/the tumor weight of the control group) × 100%.

### Hematoxylin–eosin (HE) staining

Tumor tissue was fixed in 4% paraformaldehyde, dehydrated, hyalinized, then embedded in paraffin, sliced and baked, and finally deparaffinized. The sections were stained with HE for 5–7 min for light microscopic examinations.

### Chemicals, reagents and apparatus

The reference standards of Cantharidin, Isofraxidin, Formononetin, Chlorogenic acid, Calycosin-7-glucoside, Calycosin 7-O-β-D-glucospyranoside, Astragaloside I, Astragaloside II, Astragaloside IV, Ginsenoside Rg1, Rf, Rd, Rc, Rb1, Rb2, Rb3, Re and Notoginsenoside R4 were obtained from Chengdu Lemeitian BioTechnology Co., Ltd. (Sichuan, China). All CAS of the reference standards is listed in Additional file 1: Table S2. Acetonitrile, methanol and formic acid (mass spectrometry grade) were obtained from ThermoFisher Scientific (China), whereas all other chemicals were analytical grade.

Chromatographic analysis was performed on an Ultimate 3000 (UPLC, Thermo Fisher Scientific) equipped with an online degasser, auto-sampler, column temperature controller, quaternary gradient low pressure pump, and photodiode detector (PDA). Mass spectrometry analysis was performed using a Thermo Scientific LTQ-Orbitrap XL mass spectrometer equipped with electrospray ionization (ESI), and data were acquired using Xcalibur software 2.1 (Thermo Scientific).

### Identification of ADI compounds and targets

Each of the 18 standards was individually dissolved in methanol and prepared as a stock solution at a concentration of 1 mg/ml. Exactly 100 µl of each standard reference stock solution was taken and mixed to obtain a standard solution. For the sample solution, 10 ml of ADI was passed over a rotary evaporator to remove glycerol, dissolved with methanol, and transferred to a 25 ml volumetric flask.

Chromatographic separation was performed on an ACQUITY PRM UPLC BEH C18 Column with Van Guard FI (2.1 × 100 mm,1.7 µm). The mobile phases consisted of 0.1% formic acid in water (solvent A) and acetonitrile (solvent B). The chromatographic gradient program of 66.0 min was as follows: 0 ~ 5 min, 1%B; 5 ~ 14 min, 1% ~ 15%B; 14 ~ 38 min, 15% ~ 35%B; 38 ~ 55 min, 35% ~ 55%B; 55 ~ 60 min, 55% ~ 95%B; 60 ~ 66 min, 95%B. The flow rate was 0.2 ml/min, the column temperature was 30 °C, and the sample injection volume was 2 μl.

Mass spectrometric detection was carried out in positive and negative modes using an ESI source. Nitrogen was used as an auxiliary and sheath gas at flow rates of 20 and 40 arbitrary units, while helium was used as a collision gas. The voltage of the ESI source was 3.0 kV, the temperature of the column was set at 30 °C, and the temperature of the source was 350 °C. The flow rate of the drying gas was 15 L/min and the collision voltage was 6 ~ 10 V. The primary mass spectrometry data with a mass range of 50–1200 m/z were acquired in Fourier transform high-resolution full sweep (FT, Full scan) with a resolution of 30,000.

Compounds retrieved from PubMed (https://pubmed.ncbi.nlm.nih.gov/) and China National Knowledge Infrastructure (CNKI, https://www.cnki.net/) were also used for network pharmacology analysis [13]. The SMILES structures of the compounds were collected from the PubChem database. Then, the protein targets corresponding to the chemical components of ADI were retrieved from SwissTargetPrediction (http://www.swisstargetprediction.ch/), STITCH (http://stitch.embl.de/), and Traditional Chinese Medicine Database and Analysis Platform (TCMSP, https://tcmsp-e.com/).

### Collection of targets associated with PC

The diseases corresponding targets were obtained from five resources: (1) Therapeutic Target Database (TTD, http://db.idrblab.net/ttd/); (2) Online Mendelian Inheritance in Man (OMIM, https://omim.org/); (3) MalaCardshuman disease database (https://www.malacards.org/); (4) DisGeNET (https://www.disgenet.org/); (5) GEO DataSets (https://www.ncbi.nlm.nih.gov/gds/). These databases were searched using key word such as “Pancreatic carcinoma” and “Pancreatic cancer”.

The common targets of compounds and diseases were imported into the STRING 11.0 database (https://string-db.org/) [14]. The species was limited to humans (Homo sapiens), and a confidence score higher than 0.7 were extracted to construct a protein–protein interaction (PPI) network. The networks were visualized using Cytoscape 3.3.0 (https://cytoscape.org/) and then further analyzed [15].

### Module analysis and enrichment analysis

The “Network Analyzer” function of Cytoscape software was used to analyze the topology of the PPI network and calculate three parameters (degree, betweenness, closeness) [16]. The larger the value of the above network parameters, the more important the node is. The Molecular Complex Detection (MCODE) algorithms in Cytoscape were used to analyze and extract the closely interacting node clusters in the network [17]. Finally, the following visual network was constructed: (1) Compound-putative target network of ADI. (2) Compounds-PC putative targets network. (3) PPI network of ADI-PC merge targets. (4) Module analysis network. (5) Drug-key compounds-hub targets-pathways network.

Enrichment analyses of Gene Ontology (GO) and Kyoto Encyclopedia of Genes and Genomes (KEGG) for the targets in the PPI network were performed using the “Bioconductor” package of R 4.0.4 software. The GO enrichment included three aspects, the biological process (BP), the molecular function (MF), and the cellular component (CC).

### Molecular docking

The structures of the core compound were downloaded from the PubChem database, and the three-dimensional crystal structures of the core target were obtained from the Research Collaboratory for Structural Bioinformatics (RCSB) Protein Database (PDB, https:// www. RCSB. org/). For screening, the following conditions applied: (1) the protein structure was obtained by X-ray crystal diffraction; (2) the resolution is less than 3 Å; (3) the protein structure reported by molecular docking is preferred; and (4) the biological source is Homo sapiens. Then, the protein structures were processed by AutoDock Tools, including the removal of ligands and water molecules, calculation of Gasteiger charge, the addition of polar hydrogen, and a combination of non-polar hydrogen. Finally, molecular docking was carried out via AutoDock Vina, and the results were viewed and analyzed using PyMOL (http://www.pymol.org).

### Data analysis

Statistical analyzes were performed using GraphPad Prism software version 8.01 (San Diego, California, USA). Data were presented as mean ± sd. For parametric variables, differences between groups were analyzed by one-way analysis of variance (ANOVA) or a two-tailed, unpaired Student’s t-test. Differences were considered statistically significant when *P* < 0.05.

## Results

### The ability of ADI to inhibit PC cell proliferation in vivo

**Fig. 1:**
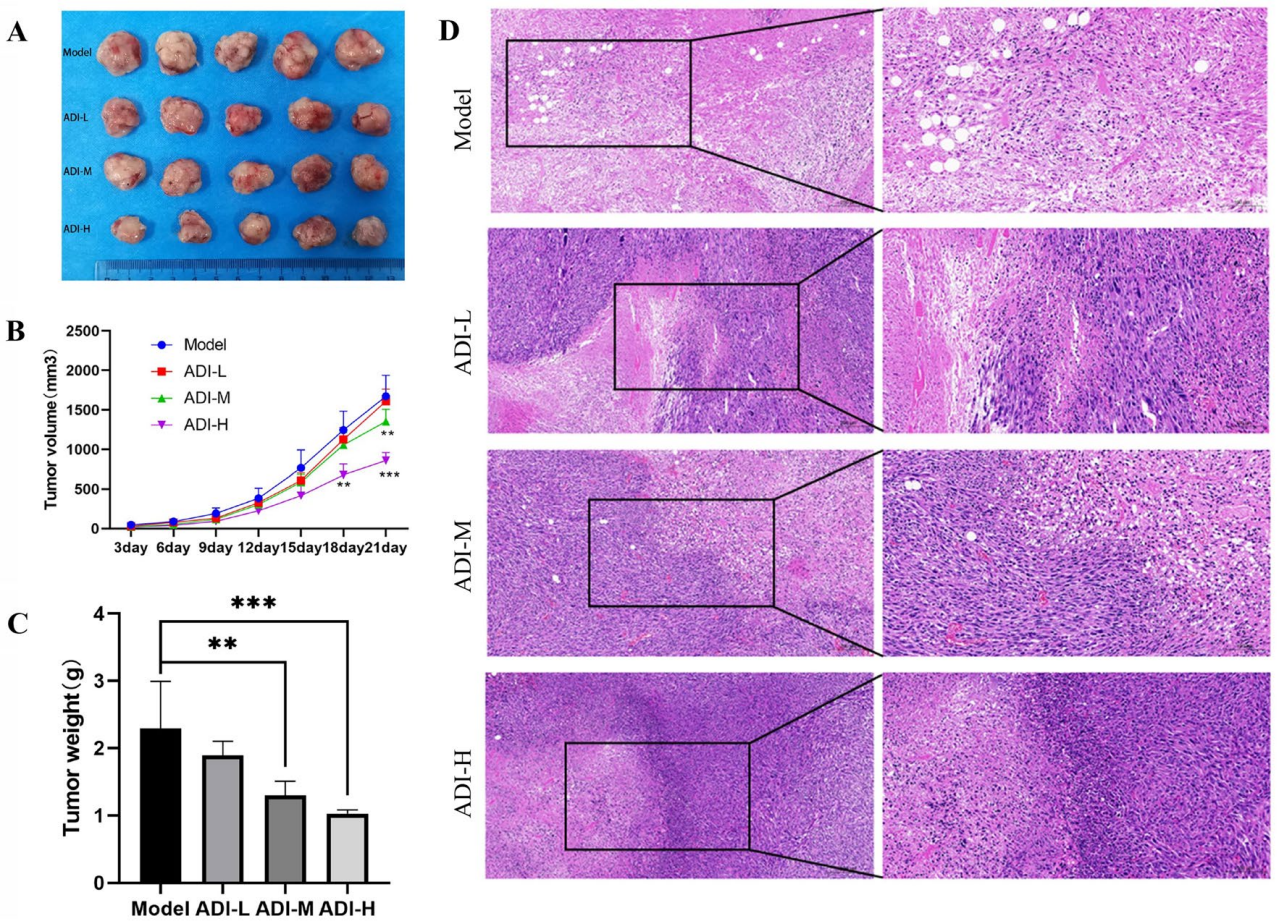
Evaluation of the therapeutic effect of ADI on the pancreatic cancer xenograft mice. **A** Subcutaneous tumors were observed in mice from four groups. **B** Growth curves of tumor volume in mice from four groups. **C** Tumor weight in mice from four groups. **D** HE staining for tumor tissues in mice from four groups. The right diagram (20 ×) is the local amplification of the left one (10 ×). ADI-L, Low-dose ADI-treated mice group; ADI-M, Medium-dose ADI-treated group; ADI-H, High-dose ADI-treated mice group. ^**^p < 0.01, ^***^p < 0.001 compared with Control group

The xenograft Panc-1 cells formed solid tumors subcutaneously in the nude mice. After 21 days of ADI administration, the tumor volume in the high-dose ADI group and the medium-dose group was significantly smaller than that in the control group (Fig. 1A, B), especially in the high-dose group. The mean tumor volume of mice in the high-dose group was markedly smaller than that of the control group from day 18. These results indicate that ADI could inhibit tumorous growth in mice bearing tumors.

After 21 days of drug administration, tumor nodules were photographed and their weights were measured. The weights of tumors treated with middle and high doses of ADI were significantly lower than those of the control groups (Fig. 1C). The means, standard deviations, and group comparisons for tumor weights, and inhibition rates are shown in Table 1. The inhibition rates of tumor weight in the low, medium, and high dose groups were 17.51%, 43.42% and 55.13%, respectively. The data show that the tumor inhibition rate improved with the increase of ADI concentration.

**Table 1:** Weight of subcutaneous tumors and inhibition rate resulting from ADI-treatment in mice

Group	Numbers	Weight (g) (mean ± *sd*)	Inhibition rates (%)
Control	5	2.29 ± 0.70	–
ADI-L	5	1.89 ± 0.21	17.51
ADI-M	5	1.30 ± 0.21^**^	43.42
ADI-H	5	1.03 ± 0.05^***^	55.13

*ADI-L* Low-dose ADI-treated mice group, *ADI-M* Medium-dose ADI-treated mice group, *ADI-H* High-dose ADI-treated mice group

^**^p < 0.01, ^***^p < 0.001 compared with Control group

The results of HE staining indicated that the tumors of the mice in the control group had severe histological abnormalities, including: shuttle-like or irregular shape, inconsistent size, disordered arrangement, and distinct foci of necrosis (Fig. 1D). The tumor tissue exhibited massive necrosis, mitotic, and no infiltration of red blood cells, suggesting ischemic necrosis due to inadequate blood supply in a rapidly growing tumor. With the increase of ADI concentration, histological abnormalities of tumor tissue decreased: focal necrosis area and vacuoles decreased; dark inflammatory cells and red blood cell infiltration appeared in the tissue. This suggests that ADI can slow down tumor growth and increased the effect with increasing ADI concentration.

### Compound-putative target network

**Fig. 2:**
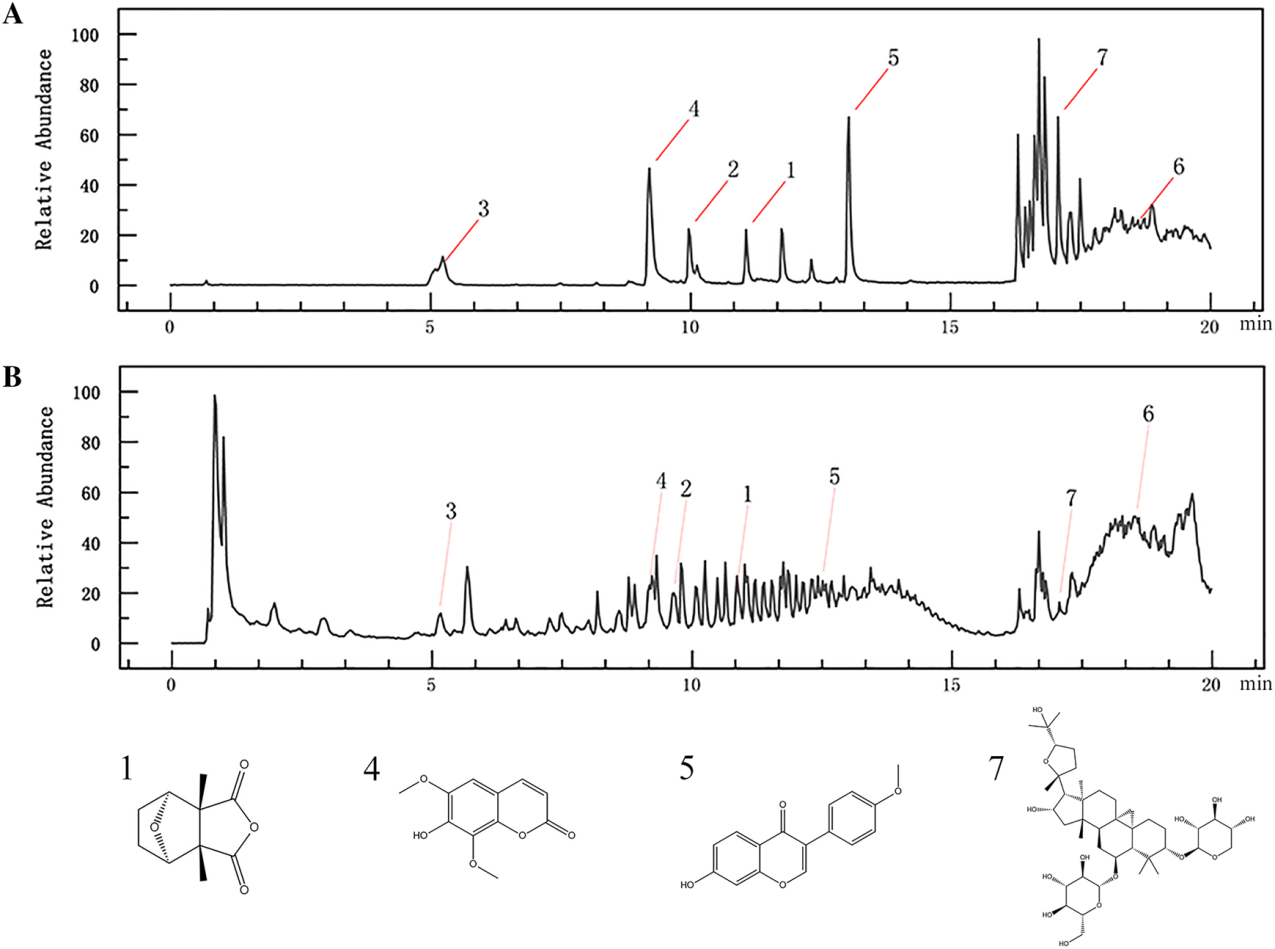
UPLC-PDA chromatogram analysis of the active compounds in ADI. **A** chromatograms of standard solution; **B** chromatograms of ADI sample solution. 1, Cantharidin; 2, Calycosin-7-glucoside; 3, Chlorogenic acid; 4, Isofraxidin; 5, Formononetin; 6, Astragaloside IV; 7, Astragaloside I

Molecular formulas were calculated using Compound Discoverer 3.0 software with a mass error of 5 ppm. Then 7 compounds were tentatively identified based on molecular weight, retention time (RT), and fragment ions in the mzCloud and mzVault databases. Finally, the identifications of the compounds were confirmed by chemical standards, including Cantharidin, Calycosin-7-glucoside, Chlorogenic acid, Sofraxidin, Formononetin, Astragaloside IV, and Astragaloside I. The data on the main fragment ions, identified compounds, molecular ions, and retention time are shown in Table 2. UHPLC-PDA shows the chromatograms of the standard solution and ADI sample solution (Fig. 2).

**Table 2:** Compounds in Aidi injection identified by UPLC-MS/MS

Peak no	Rt (min)	Measured value by ion mode		Theoretical value	Error (PPM)	Molecular formula	MS/MS ions	Chemical name
		pos	neg					
1	10.82	197.08	195.07	196.07	2.80	C_10_H_12_O_4_	133.09, 89.06	Cantharidin
2	11.52	447.13	445.11	446.12	0.15	C_22_H_22_O_10_	285.09	Calycosin-7-glucoside
3	5.15	355.10	353.09	354.10	−1.54	C_16_H_18_O_9_	162.91, 144.94, 116.84	Chlorogenic acid
4	9.20	223.06	221.05	222.05	2.47	C_11_H_10_O_5_	207.91, 162.90, 106.81	Isofraxidin
5	12.51	269.08	267.07	268.07	2.05	C_16_H_12_O_4_	253.98, 237.01, 213.04	Formononetin
6	18.53	785.47	783.45	784.46	0.70	C_41_H_68_O_14_	309.26	Astragaloside IV
7	17.06	827.48	825.46	868.48	0.63	C_45_H_72_O_16_	309.25	Astragaloside I

**Fig. 3:**
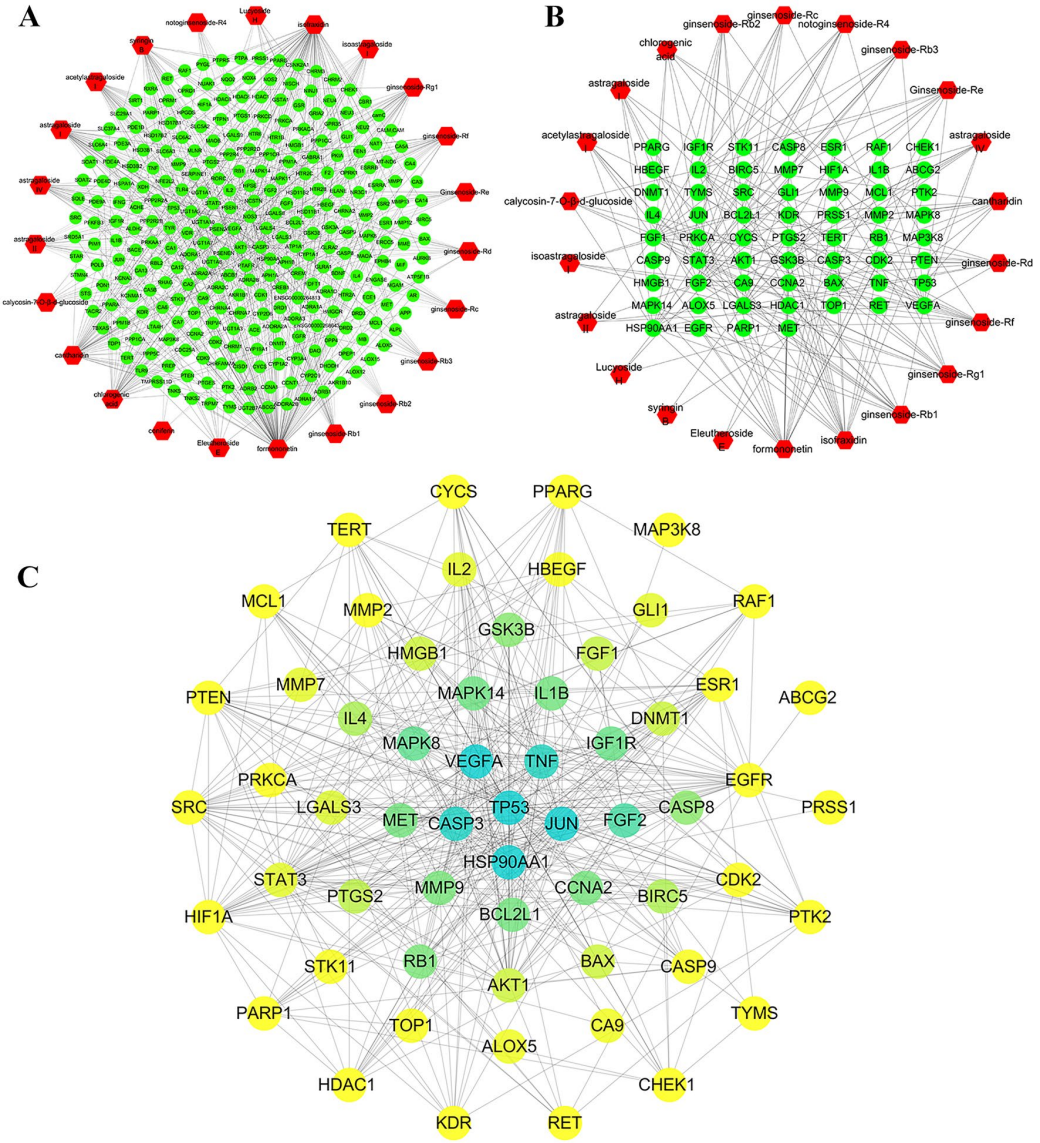
Network analyses for the ADI compound-corresponding targets implicated in PC treatment. **A** Compound-putative targets network of ADI. **B** Compounds-PC putative targets network. Red nodes represent compounds of ADI, and green nodes represent potential targets of ADI against PC. **C** PPI network of ADI-PC merge targets. The change of filled color from yellow to blue means the increased degree value

The reported chemical components in ADI were 16, and all 23 compounds from experiments and databases were listed in Additional file 1: Table S3 [18–23]. A total of 280 potential targets were identified. In the compound-putative target network, there are 303 nodes and 809 interaction edges between nodes (Fig. 3A). Among them, Isofraxidin has the highest degree (degree = 72), and the VEGFA-related nodes contain 2 targets and 14 compounds. In the generated network, different compounds can correspond to the same target, suggesting that these compounds seem to play a potential role in the simultaneous regulation of some biological processes. For example, the target AKT1 is associated with 3 compounds in ADI, including Astragaloside IV, Ginsenoside- Rb1, and Ginsenoside-Rd.

### Targets’ network and ADI-PC PPI network

A total of 585 disease targets were searched in the database. Using the ‘Merge’ function in Cytoscape, 65 intersection targets were selected for the ADI/PC-putative targets. They were entered into the STRING database to generate the PPI network. Then the compound-ADI/PC-putative therapeutic target network plot was constructed (Fig. 3B). The network contains 82 nodes (including 60 targets and 22 compounds) and 180 interaction edges between nodes. Formononetin (degree = 20) is the most important compound and targets with the highest degree value of 14 include VEGFA, LGALS3, FGF1 and FGF2.

In the generated PPI network, there are 60 nodes and 416 interaction edges among these nodes (Fig. 3C). When the color changes from yellow to blue, it indicates that the degree value of the target increases. Based on the double median of the degree (degree ≥ 14), a total of 7 core targets were selected. The betweenness centrality, closeness centrality, and details of the targets are shown in Table 3. Those targets that maintain extensive connections with other targets may be involved in common biological functions.

**Table 3:** Topological information of 7 potential core targets

UniProt ID	Gene symbol	Protein name	Betweenness centrality	Closeness centrality	Degree
P15692	*VEGFA*	Vascular endothelial growth factor A	0.02954843	0.62105263	25
P07900	*HSP90AA1*	Heat shock protein HSP 90-alpha	0.07343558	0.67816092	33
P42574	*CASP3*	Caspase-3	0.05067922	0.67045455	31
Q16539	*MAPK14*	Mitogen-activated protein kinase 14	0.00595583	0.56730769	17
P04637	*TP53*	Cellular tumor antigen P53	0.14157543	0.74683544	40
P01375	*TNF*	Tumor necrosis factor	0.01968688	0.60824742	23
P05412	*JUN*	Transcription factor AP-1	0.04203529	0.66292135	30

### Module analysis and functional enrichment analysis

**Fig. 4:**
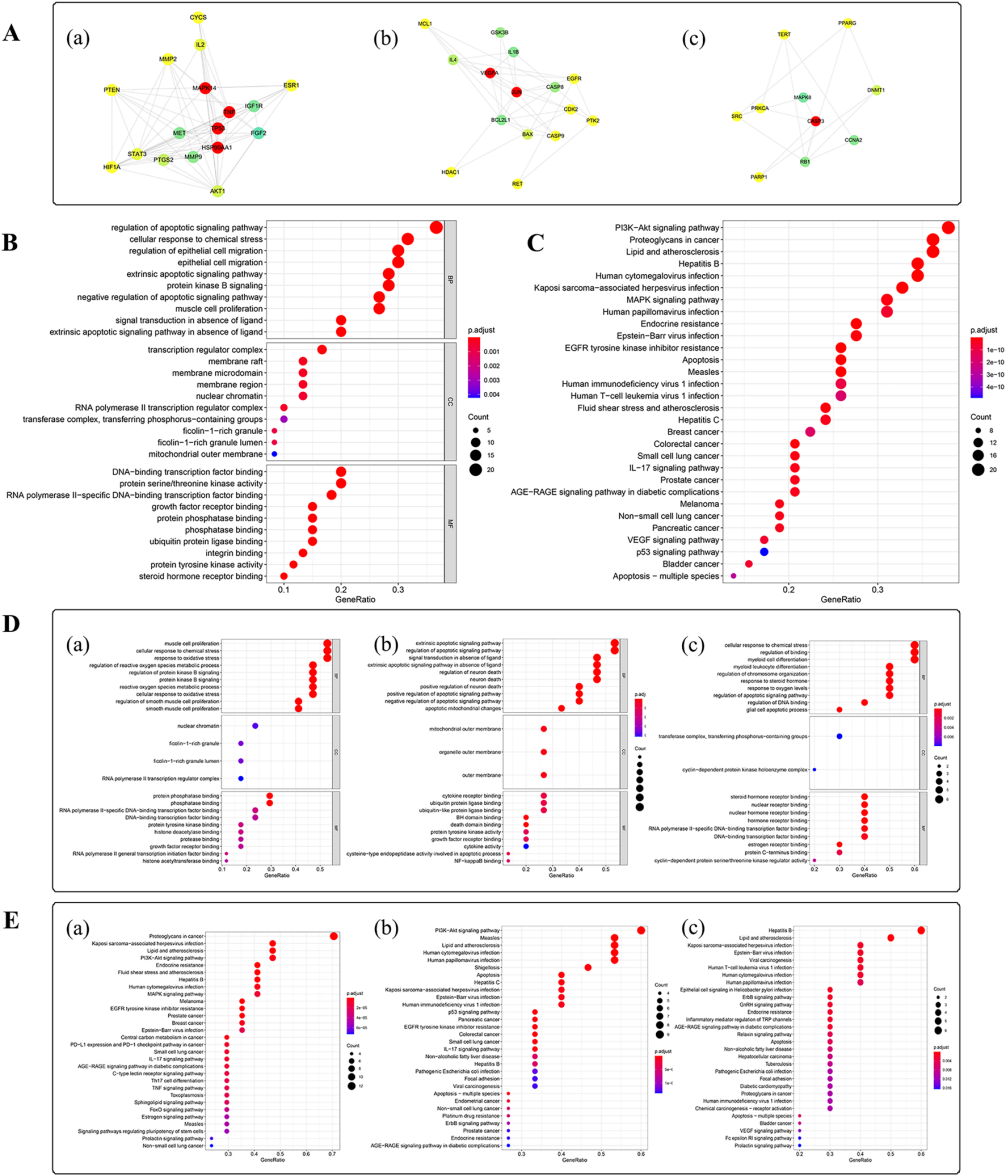
The integrated network of PPI network and modules analysis. **A** Cluster modules of the genes in the ADI-PC PPI network. From left to right is Module 1 (score = 9.125). Module 2 (score = 4.857). Module 3 (score = 3.556). Nodes marked red are the key targets that appear in the module. **B** GO enrichment analysis of target proteins in the PPI network. *p* < 0.01, *q* < 0.05. **C** KEGG pathway analysis of the target proteins in the PPI network. *p* < 0.05, *q* < 0.05. **D-E** GO and KEGG enrichment analysis of genes in each module. From left to right is Module 1, Module 2, and Module 3. *p* < 0.01 or *p* < 0.05, *q* < 0.05. The top 10 GO terms and the top 30 KEGG pathways are shown

To investigate the potential biological functions of the targets in the network, the closely related target clusters were extracted by module-constructing analysis. Finally, 3 modules (score = 9.125, 4.857, 3.556) were selected for further functional analysis (Fig. 4A).

GO functional enrichment analysis was performed for the targets in the modules, especially for the common targets, with the condition of *p* < 0.01 and *q* < 0.05 (Fig. 4B, D). The result was that module 1 was related to the BP termed muscle cell proliferation, the CC termed nuclear chromatin and the MF named protein phosphatase binding. Module 2 was involved in extrinsic apoptotic signaling (BP), outer mitochondrial membrane (CC) and cytokine receptor binding (MF). Module 3 was involved in the cellular response to chemical stress (BP), the transferase complex that transfers phosphorus − containing groups (CC), and steroid hormone receptor binding (MF). In summary, the common targets are related to the regulation of the apoptotic signaling pathway, cellular response to chemical stress and regulation of epithelial cell migration (BP), transcription regulator complex (CC) and protein serine/threonine kinase activity (MF). The information of GO enrichment analysis is shown in Additional file 1: Table S4.

In addition, the KEGG enrichment analysis was performed for these genes included in the modules to reveal their potential signaling pathways, with setting *p* < 0.05 and *q* < 0.05. As shown (Fig. 4C, E), module 1 was involved in the proteoglycans pathway in cancer, module 2 was associated with the PI3K − Akt signaling pathway and module 3 was related to the lipids and atherosclerosis signaling pathways of. The information of KEGG enrichment analysis is shown in Additional file 1: Table S5.

### Molecular docking

**Fig. 5:**
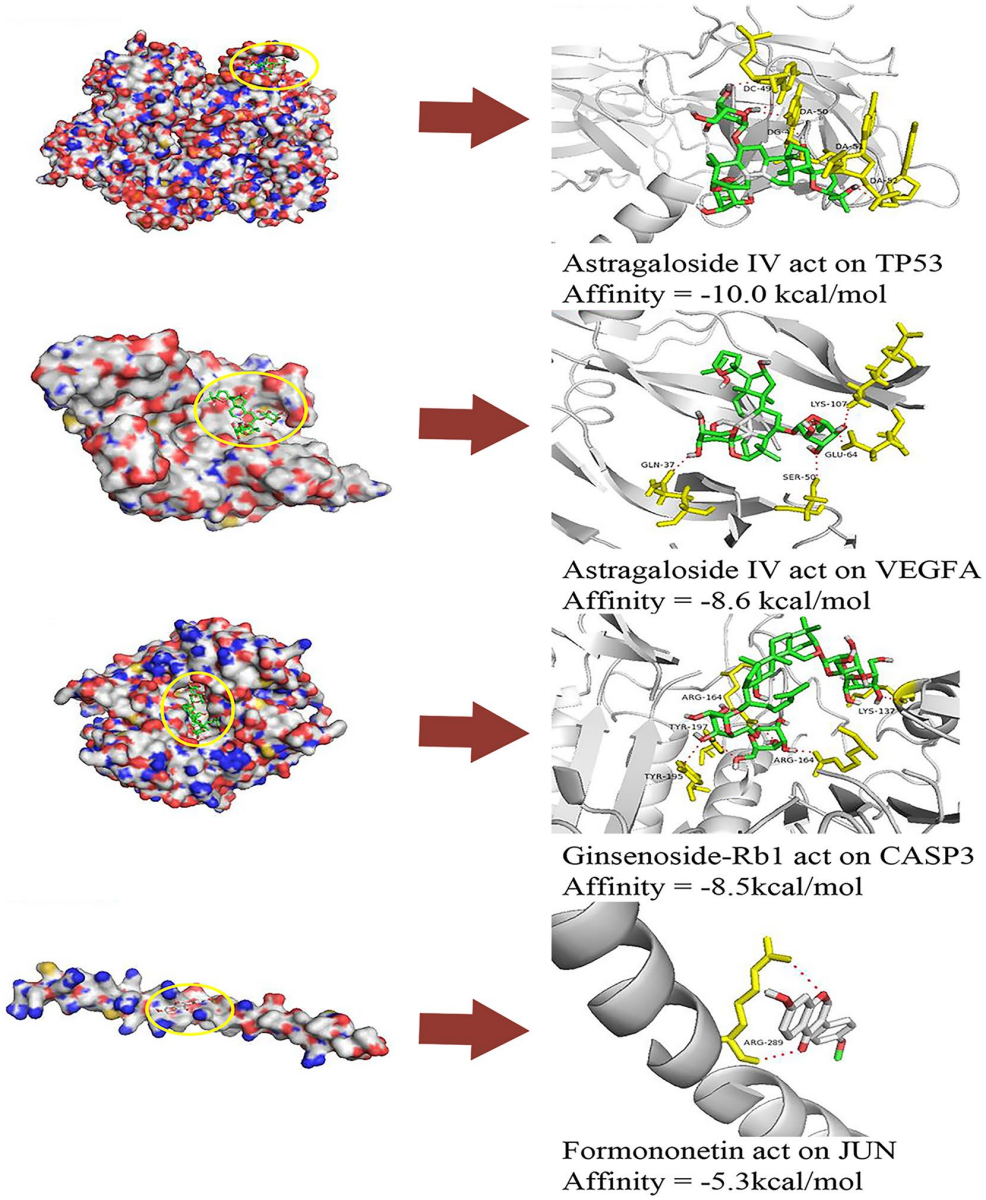
Molecular docking of the core targets and their corresponding compounds

To further verify the direct binding between the core targets and the ADI compounds, the 6 targets including P53, VEGFA, CASP3, JUN, MAPK14 and TNF were used for molecular docking verification. The top 25 compound-target pairs with high affinity are listed in Table 4. Combined with the network topology characteristics and the results of the module analysis targets, molecular docking was performed for 4 core targets (P53, VEGFA, CASP3, and JUN). The binding sites of the four protein-compound groups with the strongest affinity were visualized (Fig. 5). Astragaloside IV mainly forms 6 hydrogen bonds with residues DC-49, DA-50, DG-4, DA-51and DA-52 on P53 protein. Ginsenoside Rb1 mainly forms 5 hydrogen bonds with residues ARG-164, TYR-197, TYR-195, ARG-164, and LYS-137 on CASP3 protein. Formononetin forms mainly 2 hydrogen bonds with residues ARG-289 on JUN protein.

**Table 4:** The molecular docking results of the hub genes with the components of ADI

No	Targets	PDB ID	Compounds	Affinity (kcal/mol)
1	TNF	1tnf	Calycosin-7-O-Β-D-Glucoside	−10.2
2	P53	3q05	Astragaloside IV	−10.0
3	VEGFA	6zcd	Astragaloside IV	−8.6
4	TNF	1tnf	Ginsenoside-Rf	−8.5
5	CASP3	6zfl	Ginsenoside-Rb1	−8.5
6	MAPK14	5eti	Formononetin	−8.3
7	VEGFA	6zcd	Notoginsenoside-R4	−8.1
8	CASP3	6zfl	Formononetin	−8.0
9	VEGFA	6zcd	Isoastragaloside I	−8.0
10	VEGFA	6zcd	Acetylastragaloside I	−8.0
11	VEGFA	6zcd	Ginsenoside-Rb3	−7.9
12	VEGFA	6zcd	Ginsenoside-Rb2	−7.9
13	VEGFA	6zcd	Ginsenoside-Rg1	−7.7
14	VEGFA	6zcd	Ginsenoside-Rb1	−7.7
15	VEGFA	6zcd	Astragaloside II	−7.7
16	CASP3	6zfl	Chlorogenic Acid	−7.6
17	VEGFA	6zcd	Ginsenoside-Rf	−7.6
18	VEGFA	6zcd	Ginsenoside-Rc	−7.6
19	MAPK14	5eti	Ginsenoside-Rb1	−7.5
20	VEGFA	6zcd	Astragaloside I	−7.5
21	VEGFA	6zcd	Ginsenoside-Rd	−7.4
22	P53	3q05	Cantharidin	−7.1
23	VEGFA	6zcd	Ginsenoside-Re	−6.9
24	CASP3	6zfl	Cantharidin	−6.2
25	JUN	1s9k	Formononetin	−5.3

### Network of drug-key compounds, hub targets, and pathway

**Fig. 6:**
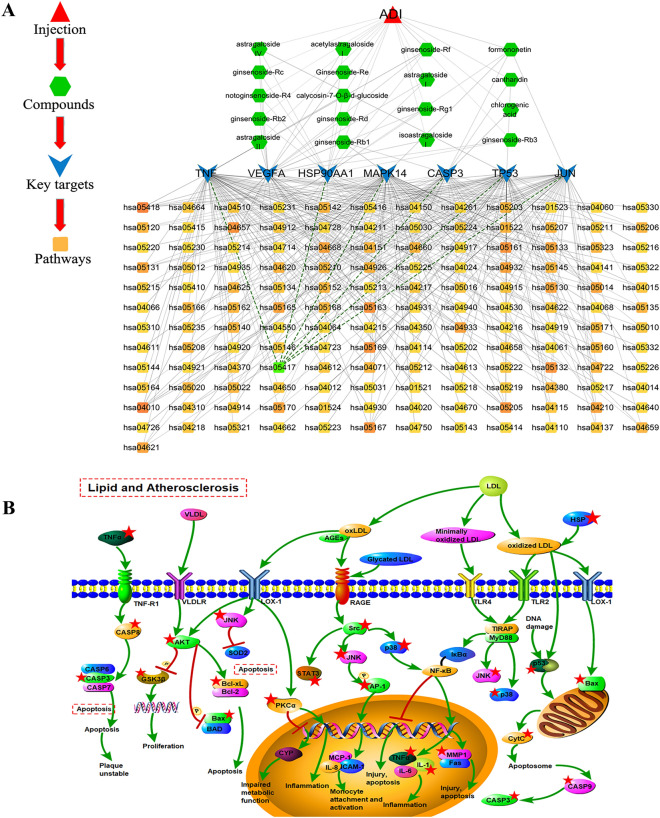
The integrated network and main signaling pathways. **A** The integrated network of key drug compounds, hub targets and signaling pathways. **B** The main signaling pathways of the targets implicated in Aidi injection-mediated PC treatment

In order to explain the mechanism of ADI in the treatment of PC, the role of key targets was analyzed and an integrated network was generated to describe the relationships between drug-key compounds, hub targets and signaling pathways. The compounds and signaling pathways were closely related to these key targets (Fig. 6A). There were 171 nodes in the network (18 compounds of ADI, 7 key targets, and 127 signaling pathways), and there were 366 edges among the nodes.

Moreover, TNF was the target with the highest degree (degree = 68), and VEGFA was the target with the most related compounds. According to the minimum *p*-value and *q*-value, the lipid and atherosclerosis pathway (hsa05417) was considered the most important KEGG pathway. Portable Pathway Builder Tool 2.0 was used to draw a cartoon map of this pathway and the targets with red asterisks indicate the potential targets of ADI (Fig. 6B).

## Discussion

According to the theory of TCM, in the advanced stage of cancer, the human body is gradually weakened, and the treatment at this time should be mainly based on strengthening the body and tonifying the deficiency, supplemented by activating *qi* and promoting blood circulation, detoxification and dispersion [24]. The extract of Ginseng Radix et Rhizoma, Astragali Radix, and Acanthopanacis Senticosi Radix Et Rhizoma Seu Caulis have a good curative effect on improving immunity. The monarch medicine Cantharidin is a commonly used animal poisonous Chinese medicine. Its toxicity can play a counteracting role in some diseases and syndromes. Combined with its effect of dredging meridians and removing stagnation, it has a good therapeutic effect on tumor masses. Meanwhile, studies have shown that Ginseng, Astragalus, and Acanthopanax alone can inhibit the growth of tumor cells. When used together with Cantharidin, they can show significant synergistic effects and inhibit tumor growth [25, 26]. In a clinical trial, ADI was clearly found to have better clinical efficacy than chemotherapy alone in the treatment of PC in combination with chemotherapy and reduced the incidence of adverse effects such as leukopenia, thrombocytopenia, and gastrointestinal reactions [27].

In this study, a PC tumor-bearing mouse model was first established. After 21 days of the administration, it was found that the tumor volume and weight of mice in the middle and high dose groups of ADI were significantly smaller than those in the model group. The growth rate of tumor tissue, the degree of necrosis, and the number of cavities in tumor tissue were significantly reduced. This indicated that ADI could inhibit tumor growth in PC tumor-bearing mice. Then, the network pharmacology method was used to initially explore the mechanism of ADI in the treatment of PC. The important compounds in ADI were analyzed, and there were 22 compounds with potential therapeutic effects on PC. Network pharmacology predicted that there were seven core targets for ADI to treat PC, including TNF, VEGFA, HSP90AA1, MAPK14, CASP3, P53, and JUN.

Combined with the network topology characteristics and the results of the module analysis of targets, this study suggests that these 4 targets (P53, VEGFA, CASP3, and JUN) are potential therapeutic targets for ADI treatment of PC and molecular docking was performed. The full name of P53 is tumor protein P53 (TP53). The protein encoded by the wild-type gene may be involved in cell biological processes such as cell cycle induction and arrest. Its mutation is closely related to the occurrence and development of various human cancers. Previous studies have shown that *P53* is one of the four major genes contributing to PC, and the mutant P53 protein has significantly high expression in the nucleus of PC cells [28–31]. VEGFA (Vascular endothelial growth factor A) is a member of the PDGF/VEGF growth factor family, mainly secreted by endothelial cells and is widely distributed in the human body. It has the functions of stimulating endothelial cell proliferation, promoting angiogenesis, and improving vascular permeability. VEGFA is expressed in almost all types of malignant tumors and is currently still an important target for anti-angiogenesis drugs targeting tumors [32]. A research report based on molecular dynamics simulations and experimental verification showed that Astragaloside IV can inhibit the proliferation, migration, and invasion of human hepatoma HepG2 cells. The study predicted that the core gene of Astragaloside IV intervention is VEGFA, which is mutually confirmed by the molecular docking results of this study. In addition, the study demonstrated that Astragaloside IV can downregulate the expression level of VEGFA mRNA in HepG2 cells through RT-qPCR experiments [33]. CASP3 is a cysteine protease, also known as CASPASE-3 or CPP32. It is mainly responsible for cleaving DNA repair enzymes such as PARP, inactivating their function and finally causing cell apoptosis. One study has shown that the silencing of CCNB1 protein can increase the expression of CASP3 and P53 by activating the P53 signalig pathway, leading to an increase in the proportion of apoptosis and senescence of PC cells [34]. Recent reports suggest that CASP3 can not only mediate apoptosis, but also promote tumor recurrence and angiogenesis. Knockout of CASP3 in colon cancer cells resulted in cancer cells being more sensitive to radiation in *vitro* and in *vivo*, and most importantly their invasiveness and metastasis were significantly reduced [35]. These phenomena suggest that the reduction of CASP3 may improve the sensitivity of cancer cells to radiotherapy and chemotherapy and inhibit cancer cell invasion and metastasis. JUN is a subunit of the AP-1 complex, which is known to be a widely studied protein in the AP-1 family. The locus of JUN is located in the chromosomal region closely associated with human malignant tumors, and JUN is involved in various biological activities including cell growth, proliferation and apoptosis [36]. A study related to the invasiveness of PC cells found that, β2-adrenergic antagonists can reduce the invasion and proliferation of PC cells by inhibiting AP-1 and its associated signaling pathways [37]. However, the function of AP-1 appears to be cell-specific, and its biological function depends on the expression of members of the AP-1 complex. As reported, gemcitabine significantly increased the expression of JUN in Panc-1 cells [38].

Molecular docking results show that Astragaloside IV, Ginsenoside Rb1, and Formononetin have good affinity with core targets P53, VEGFA, CASP3, and JUN, respectively. They are considered as potential therapeutic compounds of ADI in the treatment of PC. Astragaloside IV, the main pharmacological components in the Astragali Radix, has anti-tumor effects, improves immune function, and has protective effects on the cardiovascular system, brain, lung, kidney, and other organs [39, 40]. In this study, Astragaloside IV showed high affinity for P53. Zhao et al. indicated that Astragaloside IV regulates the cell cycle of vulvar squamous cell carcinoma cells by upregulating the expression of P53 and P21 and downregulating the expression of cyclin D1 [41]. Formononetin is the main isoflavone component from Astragali Radix. Several studies have shown that formononetin can suppress the proliferation and metastasis of human bladder cancer cells and ovarian cancer cells [42, 43]. Furthermore, Formononetin can inhibit the growth of human colon cancer cells, promote apoptosis, and inhibit tumor invasion by downregulating the expression of pro-angiogenic factors in the tumor xenograft [44]. In addition, Formononetin has also shown a protective effect on the cytotoxicity of chemotherapy drugs. Thus, Formononetin treatment reduced cisplatin-induced cytotoxicity in LLC-PK1 pig kidney epithelial cells, which may be a potential therapeutic agent for cervical cancer to prevent cisplatin induced nephrotoxicity [45]. Ginsenoside Rb1, a major bioactive ingredient of Ginseng Radix et Rhizoma, could enhance the sensitivity to anti-tumor drugs and regulate the body’s immune function against cancer cells [46, 47]. Ginsenoside Rb1 can significantly reduce the inhibitory effect of tumor cells on NK cell exocytosis and cytotoxicity. And the antagonistic effect was significantly strongest when combined with Astragali Radix [48]. In a cancer cachexia mouse model injected with colon cancer cells, Ginsenoside Rb1 was able to reduce TNF-α and IL-6 cytokine levels, ameliorating the symptoms of cancer cachexia.

## Conclusion

ADI could reduce the growth rate of tumor tissue and alleviate the structural abnormalities in tumor tissue. ADI is predicted to act on VEGFA, P53, CASP3, and JUN in ADI-mediated treatment of PC.

## Supplementary Information

**Additional file 1: Table S1.** Information about the Aidi injection.

**Additional file 2: Table S2.** The CAS of reference standards.

**Additional file 3: Table S3.** Compounds in Aidi Injection.

**Additional file 4: Table S4.** The information of GO enrichment analysis of ADI-pancreatic cancer PPI network.

**Additional file 5: Table S5.** The information of KEGG enrichment analysis of ADI-pancreatic cancer PPI network.

## Data Availability

The data used to support the current study are available from the corresponding author on reasonable request.

## Abbreviations

ADI: Aidi injection
BP: Biological process
CASP3: Caspase 3
CC: Cellular component
DMEM: Dulbecco’s modified Eagle’s medium
ESI: Electrospray ionization
FBS: Fetal bovine serum
FGF1: Fibroblast growth factor 1
FGF2: Fibroblast growth factor 2
GO: Gene ontology
HE: Hematoxylin–eosin
HSP90AA1: Heat shock protein 90 alpha family class a member 1
JUN: Jun proto-oncogene
AP-1: Transcription factor subunit
KEGG: Kyoto encyclopedia of genes and genomes
LGALS3: Galectin 3
MAPK14: Mitogen-activated protein kinase 14
MF: Molecular function
P53: Tumor protein P53
PC: Pancreatic cancer
SPF: Specific pathogen-free
TCM: Traditional Chinese medicine
TNF: Tumor necrosis factor
VEGFA: Vascular endothelial growth factor A
