# Supplementary material for: Identification of key pharmacological components and targets for Aidi injection in the treatment of pancreatic cancer by UPLC-MS, network pharmacology, and in vivo experiments
Source: Chin Med. 2023 Jan 14;18:7. doi: 10.1186/s13020-023-00710-2 (PMC9840244; doi:10.1186/s13020-023-00710-2)
Supplement: Supplementary file 1 — Additional file 1: Table S1. Information about the Aidi injection. [file 13020_2023_710_MOESM1_ESM.docx]

**Table S1.** **Information about the Aidi injection**

| **injection** | Aidi injection |
| --- | --- |
| **Source** | Guizhou Yibai Pharmaceutical Co., Ltd. |
| **Species /Raw materials** | Mylabris 1.5g (animal drug), Ginseng Radix Et Rhizoma 50g, Astmgali Radix 100g, Acanthopanacis Senticosi Radix Et Rhizoma Seu Caulis 150g, Astmgali Radix 20g |
| **Botanical plant names** | *Panax ginseng* C.A.Mey.; *Astragalus mongholicus* Bunge; *Eleutherococcus senticosus* (Rupr. & Maxim.) Maxim |
| **Function** | Clearing heat and detoxifying, dissipating blood stasis and removing knots |
| **Indication** | Primary liver cancer, lung cancer, rectal cancer, malignant lymphoma, gynecological malignant tumors, etc. |
| **Quality control reported? (Y/N)** | Y-National Pharmaceutical Standard Z52020236; Standard number: WS3-B-3809-99-2002 |
