# Supplementary material for: Identification of key pharmacological components and targets for Aidi injection in the treatment of pancreatic cancer by UPLC-MS, network pharmacology, and in vivo experiments
Source: Chin Med. 2023 Jan 14;18:7. doi: 10.1186/s13020-023-00710-2 (PMC9840244; doi:10.1186/s13020-023-00710-2)
Supplement: Supplementary file 2 — Additional file 2: Table S2. The CAS of reference standards. [file 13020_2023_710_MOESM2_ESM.docx]

**Table S1 The CAS of reference standards**

| Compounds | CAS | Compounds | CAS |
| --- | --- | --- | --- |
| Cantharidin | 56-25-7 | Ginsenoside Rg1 | 22427-39-0 |
| Isofraxidin | 486-21-5 | Ginsenoside Rf | 52286-58-5 |
| Formononetin | 485-72-3 | Ginsenoside Rd | 52705-93-8 |
| Chlorogenic acid | 327-97-9 | Ginsenoside Rc, | 11021-14-0 |
| Calycosin-7-glucoside | 20633-67-4 | Ginsenoside Rb1 | 41753-43-9 |
| Calycosin 7-O-β-D-glucospyranoside | 20633-67-4 | Ginsenoside Rb2 | 11021-13-9 |
| Astragaloside І | 84680-75-1 | Ginsenoside Rb3 | 68406-26-8 |
| Astragaloside II | 84676-89-1 | Ginsenoside Re | 52286-59-6 |
| Astragaloside Ⅳ | 84687-43-4 | Notoginsenoside R4 | 87741-77-3 |
