# Supplementary material for: Identification of key pharmacological components and targets for Aidi injection in the treatment of pancreatic cancer by UPLC-MS, network pharmacology, and in vivo experiments
Source: Chin Med. 2023 Jan 14;18:7. doi: 10.1186/s13020-023-00710-2 (PMC9840244; doi:10.1186/s13020-023-00710-2)
Supplement: Supplementary file 3 — Additional file 3: Table S3. Compounds in Aidi Injection. [file 13020_2023_710_MOESM3_ESM.docx]

**Table S2 Compounds in Aidi Injection**

| Compound name | Compound name |
| --- | --- |
| cantharidin | ginsenoside-Re |
| isofraxidin | ginsenoside-Rb1 |
| syringin B | ginsenoside-Rf |
| Eleutheroside E | ginsenoside-Rb3 |
| formononetin | notoginsenoside-R4 |
| astragaloside IV | ginsenoside-Rc |
| chlorogenic acid | ginsenoside-Rb2 |
| astragaloside Ⅱ | ginsenoside-Rd |
| astragaloside I | Lucyoside H |
| isoastragaloside I | coniferin |
| acetylastragaloside I | calycosin-7-O-β-d-glucoside |
| ginsenoside-Rg1 |  |
