# Supplementary material for: Identification of key pharmacological components and targets for Aidi injection in the treatment of pancreatic cancer by UPLC-MS, network pharmacology, and in vivo experiments
Source: Chin Med. 2023 Jan 14;18:7. doi: 10.1186/s13020-023-00710-2 (PMC9840244; doi:10.1186/s13020-023-00710-2)
Supplement: Supplementary file 4 — Additional file 4: Table S4. The information of GO enrichment analysis of ADI-pancreatic cancer PPI network. [file 13020_2023_710_MOESM4_ESM.docx]

# Table S3 The information of GO enrichment analysis of ADI-pancreatic cancer PPI network.

| **Ontology** | **ID** | **Description** | **pvalue** | **p.adjust** | **qvalue** | **Count** |
| --- | --- | --- | --- | --- | --- | --- |
| BP | GO:2001233 | regulation of apoptotic signaling pathway | 9.97E-22 | 3.32E-18 | 1.33E-18 | 22 |
| BP | GO:0097191 | extrinsic apoptotic signaling pathway | 2.94E-19 | 4.90E-16 | 1.96E-16 | 17 |
| BP | GO:0010632 | regulation of epithelial cell migration | 8.94E-19 | 7.98E-16 | 3.19E-16 | 18 |
| BP | GO:0062197 | cellular response to chemical stress | 9.58E-19 | 7.98E-16 | 3.19E-16 | 19 |
| BP | GO:0038034 | signal transduction in absence of ligand | 4.99E-18 | 2.78E-15 | 1.11E-15 | 12 |
| BP | GO:0097192 | extrinsic apoptotic signaling pathway in absence of ligand | 4.99E-18 | 2.78E-15 | 1.11E-15 | 12 |
| BP | GO:0043491 | protein kinase B signaling | 6.66E-18 | 3.17E-15 | 1.27E-15 | 17 |
| BP | GO:2001234 | negative regulation of apoptotic signaling pathway | 1.54E-17 | 6.42E-15 | 2.56E-15 | 16 |
| BP | GO:0010631 | epithelial cell migration | 2.65E-17 | 9.32E-15 | 3.72E-15 | 18 |
| BP | GO:0033002 | muscle cell proliferation | 2.85E-17 | 9.32E-15 | 3.72E-15 | 16 |
| BP | GO:0090132 | epithelium migration | 3.08E-17 | 9.32E-15 | 3.72E-15 | 18 |
| BP | GO:0090130 | tissue migration | 4.14E-17 | 1.15E-14 | 4.59E-15 | 18 |
| BP | GO:0050673 | epithelial cell proliferation | 5.26E-17 | 1.35E-14 | 5.39E-15 | 19 |
| BP | GO:0050678 | regulation of epithelial cell proliferation | 9.31E-17 | 2.22E-14 | 8.85E-15 | 18 |
| BP | GO:0018209 | peptidyl-serine modification | 1.37E-16 | 3.05E-14 | 1.22E-14 | 17 |
| BP | GO:0001667 | ameboidal-type cell migration | 1.67E-16 | 3.47E-14 | 1.39E-14 | 19 |
| BP | GO:0010634 | positive regulation of epithelial cell migration | 2.05E-16 | 3.82E-14 | 1.52E-14 | 14 |
| BP | GO:0071214 | cellular response to abiotic stimulus | 2.18E-16 | 3.82E-14 | 1.52E-14 | 17 |
| BP | GO:0104004 | cellular response to environmental stimulus | 2.18E-16 | 3.82E-14 | 1.52E-14 | 17 |
| BP | GO:0030099 | myeloid cell differentiation | 4.99E-16 | 8.32E-14 | 3.32E-14 | 18 |
| BP | GO:0018105 | peptidyl-serine phosphorylation | 9.95E-16 | 1.58E-13 | 6.31E-14 | 16 |
| BP | GO:0051896 | regulation of protein kinase B signaling | 1.14E-15 | 1.69E-13 | 6.74E-14 | 15 |
| BP | GO:0034599 | cellular response to oxidative stress | 1.16E-15 | 1.69E-13 | 6.74E-14 | 16 |
| BP | GO:0006979 | response to oxidative stress | 2.04E-15 | 2.83E-13 | 1.13E-13 | 18 |
| BP | GO:0043281 | regulation of cysteine-type endopeptidase activity involved in apoptotic process | 5.13E-15 | 6.84E-13 | 2.73E-13 | 14 |
| BP | GO:0048660 | regulation of smooth muscle cell proliferation | 6.14E-15 | 7.87E-13 | 3.14E-13 | 13 |
| BP | GO:0048659 | smooth muscle cell proliferation | 7.16E-15 | 8.84E-13 | 3.53E-13 | 13 |
| BP | GO:0070997 | neuron death | 1.07E-14 | 1.28E-12 | 5.09E-13 | 16 |
| BP | GO:0097193 | intrinsic apoptotic signaling pathway | 1.39E-14 | 1.60E-12 | 6.38E-13 | 15 |
| BP | GO:0048608 | reproductive structure development | 1.71E-14 | 1.90E-12 | 7.59E-13 | 17 |
| BP | GO:0048661 | positive regulation of smooth muscle cell proliferation | 1.83E-14 | 1.96E-12 | 7.84E-13 | 11 |
| BP | GO:0061458 | reproductive system development | 1.92E-14 | 2.00E-12 | 7.97E-13 | 17 |
| BP | GO:2000116 | regulation of cysteine-type endopeptidase activity | 2.23E-14 | 2.25E-12 | 8.99E-13 | 14 |
| BP | GO:0052547 | regulation of peptidase activity | 3.72E-14 | 3.65E-12 | 1.46E-12 | 17 |
| BP | GO:1901214 | regulation of neuron death | 4.48E-14 | 4.27E-12 | 1.70E-12 | 15 |
| BP | GO:2001236 | regulation of extrinsic apoptotic signaling pathway | 6.92E-14 | 6.41E-12 | 2.56E-12 | 12 |
| BP | GO:1903829 | positive regulation of cellular protein localization | 7.42E-14 | 6.69E-12 | 2.67E-12 | 15 |
| BP | GO:0009612 | response to mechanical stimulus | 1.03E-13 | 9.05E-12 | 3.61E-12 | 13 |
| BP | GO:0052548 | regulation of endopeptidase activity | 2.35E-13 | 2.01E-11 | 8.02E-12 | 16 |
| BP | GO:0045862 | positive regulation of proteolysis | 3.17E-13 | 2.59E-11 | 1.03E-11 | 15 |
| BP | GO:0051897 | positive regulation of protein kinase B signaling | 3.19E-13 | 2.59E-11 | 1.03E-11 | 12 |
| BP | GO:1901216 | positive regulation of neuron death | 3.93E-13 | 3.12E-11 | 1.24E-11 | 10 |
| BP | GO:0048511 | rhythmic process | 4.02E-13 | 3.12E-11 | 1.24E-11 | 14 |
| BP | GO:0009411 | response to UV | 7.67E-13 | 5.81E-11 | 2.32E-11 | 11 |
| BP | GO:2001237 | negative regulation of extrinsic apoptotic signaling pathway | 1.11E-12 | 8.19E-11 | 3.27E-11 | 10 |
| BP | GO:0010506 | regulation of autophagy | 1.70E-12 | 1.23E-10 | 4.90E-11 | 14 |
| BP | GO:0002573 | myeloid leukocyte differentiation | 1.85E-12 | 1.31E-10 | 5.24E-11 | 12 |
| BP | GO:0032147 | activation of protein kinase activity | 2.08E-12 | 1.44E-10 | 5.76E-11 | 14 |
| BP | GO:0006914 | autophagy | 2.47E-12 | 1.65E-10 | 6.58E-11 | 16 |
| BP | GO:0061919 | process utilizing autophagic mechanism | 2.47E-12 | 1.65E-10 | 6.58E-11 | 16 |
| BP | GO:0043542 | endothelial cell migration | 3.08E-12 | 1.99E-10 | 7.93E-11 | 13 |
| BP | GO:0001101 | response to acid chemical | 3.10E-12 | 1.99E-10 | 7.93E-11 | 14 |
| BP | GO:0051052 | regulation of DNA metabolic process | 4.23E-12 | 2.66E-10 | 1.06E-10 | 14 |
| BP | GO:2001242 | regulation of intrinsic apoptotic signaling pathway | 4.33E-12 | 2.67E-10 | 1.07E-10 | 11 |
| BP | GO:0048732 | gland development | 5.03E-12 | 3.05E-10 | 1.22E-10 | 15 |
| BP | GO:0034614 | cellular response to reactive oxygen species | 5.27E-12 | 3.14E-10 | 1.25E-10 | 11 |
| BP | GO:0018108 | peptidyl-tyrosine phosphorylation | 6.64E-12 | 3.76E-10 | 1.50E-10 | 14 |
| BP | GO:1904705 | regulation of vascular smooth muscle cell proliferation | 6.66E-12 | 3.76E-10 | 1.50E-10 | 9 |
| BP | GO:1990874 | vascular smooth muscle cell proliferation | 6.66E-12 | 3.76E-10 | 1.50E-10 | 9 |
| BP | GO:0010594 | regulation of endothelial cell migration | 7.24E-12 | 4.02E-10 | 1.60E-10 | 12 |
| BP | GO:0018212 | peptidyl-tyrosine modification | 7.41E-12 | 4.05E-10 | 1.62E-10 | 14 |
| BP | GO:0009314 | response to radiation | 7.90E-12 | 4.25E-10 | 1.70E-10 | 15 |
| BP | GO:0000302 | response to reactive oxygen species | 8.43E-12 | 4.46E-10 | 1.78E-10 | 12 |
| BP | GO:0001936 | regulation of endothelial cell proliferation | 8.76E-12 | 4.56E-10 | 1.82E-10 | 11 |
| BP | GO:0010595 | positive regulation of endothelial cell migration | 9.06E-12 | 4.65E-10 | 1.86E-10 | 10 |
| BP | GO:0051098 | regulation of binding | 9.55E-12 | 4.83E-10 | 1.93E-10 | 14 |
| BP | GO:0051402 | neuron apoptotic process | 1.19E-11 | 5.95E-10 | 2.37E-10 | 12 |
| BP | GO:1903708 | positive regulation of hemopoiesis | 1.51E-11 | 7.39E-10 | 2.95E-10 | 11 |
| BP | GO:0070482 | response to oxygen levels | 1.98E-11 | 9.59E-10 | 3.83E-10 | 14 |
| BP | GO:0001935 | endothelial cell proliferation | 2.13E-11 | 1.00E-09 | 3.99E-10 | 11 |
| BP | GO:0051054 | positive regulation of DNA metabolic process | 2.13E-11 | 1.00E-09 | 3.99E-10 | 11 |
| BP | GO:0062013 | positive regulation of small molecule metabolic process | 2.39E-11 | 1.11E-09 | 4.42E-10 | 10 |
| BP | GO:0022407 | regulation of cell-cell adhesion | 2.59E-11 | 1.18E-09 | 4.73E-10 | 14 |
| BP | GO:0046677 | response to antibiotic | 2.84E-11 | 1.28E-09 | 5.10E-10 | 13 |
| BP | GO:0032496 | response to lipopolysaccharide | 3.18E-11 | 1.41E-09 | 5.64E-10 | 13 |
| BP | GO:0071887 | leukocyte apoptotic process | 4.24E-11 | 1.86E-09 | 7.42E-10 | 9 |
| BP | GO:0050679 | positive regulation of epithelial cell proliferation | 4.82E-11 | 2.09E-09 | 8.34E-10 | 11 |
| BP | GO:0002237 | response to molecule of bacterial origin | 5.14E-11 | 2.20E-09 | 8.77E-10 | 13 |
| BP | GO:0071229 | cellular response to acid chemical | 5.64E-11 | 2.38E-09 | 9.49E-10 | 11 |
| BP | GO:0043523 | regulation of neuron apoptotic process | 5.93E-11 | 2.47E-09 | 9.87E-10 | 11 |
| BP | GO:0001938 | positive regulation of endothelial cell proliferation | 8.31E-11 | 3.42E-09 | 1.37E-09 | 9 |
| BP | GO:0001666 | response to hypoxia | 9.05E-11 | 3.68E-09 | 1.47E-09 | 13 |
| BP | GO:0010038 | response to metal ion | 1.00E-10 | 3.98E-09 | 1.59E-09 | 13 |
| BP | GO:0031331 | positive regulation of cellular catabolic process | 1.00E-10 | 3.98E-09 | 1.59E-09 | 13 |
| BP | GO:0036293 | response to decreased oxygen levels | 1.31E-10 | 5.15E-09 | 2.06E-09 | 13 |
| BP | GO:2001239 | regulation of extrinsic apoptotic signaling pathway in absence of ligand | 1.40E-10 | 5.44E-09 | 2.17E-09 | 7 |
| BP | GO:0043536 | positive regulation of blood vessel endothelial cell migration | 1.53E-10 | 5.80E-09 | 2.31E-09 | 8 |
| BP | GO:0071260 | cellular response to mechanical stimulus | 1.53E-10 | 5.80E-09 | 2.31E-09 | 8 |
| BP | GO:0042110 | T cell activation | 1.72E-10 | 6.45E-09 | 2.58E-09 | 14 |
| BP | GO:0071453 | cellular response to oxygen levels | 1.89E-10 | 6.99E-09 | 2.79E-09 | 11 |
| BP | GO:1904707 | positive regulation of vascular smooth muscle cell proliferation | 1.91E-10 | 6.99E-09 | 2.79E-09 | 7 |
| BP | GO:0008637 | apoptotic mitochondrial changes | 2.08E-10 | 7.55E-09 | 3.01E-09 | 9 |
| BP | GO:0010821 | regulation of mitochondrion organization | 2.54E-10 | 8.98E-09 | 3.58E-09 | 10 |
| BP | GO:2001235 | positive regulation of apoptotic signaling pathway | 2.54E-10 | 8.98E-09 | 3.58E-09 | 10 |
| BP | GO:0050999 | regulation of nitric-oxide synthase activity | 2.56E-10 | 8.98E-09 | 3.58E-09 | 7 |
| BP | GO:0009416 | response to light stimulus | 2.82E-10 | 9.81E-09 | 3.92E-09 | 12 |
| BP | GO:0048871 | multicellular organismal homeostasis | 3.16E-10 | 1.09E-08 | 4.33E-09 | 14 |
| BP | GO:0007568 | aging | 3.64E-10 | 1.24E-08 | 4.94E-09 | 12 |
| BP | GO:0045785 | positive regulation of cell adhesion | 3.75E-10 | 1.26E-08 | 5.05E-09 | 13 |
| BP | GO:0045637 | regulation of myeloid cell differentiation | 3.99E-10 | 1.33E-08 | 5.31E-09 | 11 |
| CC | GO:0005667 | transcription regulator complex | 4.51E-07 | 9.21E-05 | 6.75E-05 | 10 |
| CC | GO:0045121 | membrane raft | 4.83E-06 | 0.000331 | 0.000243 | 8 |
| CC | GO:0098857 | membrane microdomain | 4.94E-06 | 0.000331 | 0.000243 | 8 |
| CC | GO:0098589 | membrane region | 6.49E-06 | 0.000331 | 0.000243 | 8 |
| CC | GO:0090575 | RNA polymerase II transcription regulator complex | 1.12E-05 | 0.000456 | 0.000334 | 6 |
| CC | GO:0000790 | nuclear chromatin | 1.75E-05 | 0.000594 | 0.000436 | 8 |
| CC | GO:0101002 | ficolin-1-rich granule | 3.76E-05 | 0.000958 | 0.000702 | 5 |
| CC | GO:1904813 | ficolin-1-rich granule lumen | 3.76E-05 | 0.000958 | 0.000702 | 5 |
| CC | GO:0061695 | transferase complex, transferring phosphorus-containing groups | 0.000134 | 0.003038 | 0.002226 | 6 |
| CC | GO:0005741 | mitochondrial outer membrane | 0.000207 | 0.004226 | 0.003097 | 5 |
| CC | GO:0016605 | PML body | 0.000235 | 0.004359 | 0.003194 | 4 |
| CC | GO:0000307 | cyclin-dependent protein kinase holoenzyme complex | 0.000283 | 0.004805 | 0.003521 | 3 |
| CC | GO:1902911 | protein kinase complex | 0.00034 | 0.005166 | 0.003785 | 4 |
| CC | GO:0031968 | organelle outer membrane | 0.000363 | 0.005166 | 0.003785 | 5 |
| CC | GO:0019867 | outer membrane | 0.00038 | 0.005166 | 0.003785 | 5 |
| CC | GO:0031983 | vesicle lumen | 0.000469 | 0.005974 | 0.004377 | 6 |
| CC | GO:0098687 | chromosomal region | 0.00064 | 0.007681 | 0.005628 | 6 |
| MF | GO:0070851 | growth factor receptor binding | 6.66E-10 | 1.53E-07 | 8.70E-08 | 9 |
| MF | GO:0019903 | protein phosphatase binding | 9.84E-10 | 1.53E-07 | 8.70E-08 | 9 |
| MF | GO:0140297 | DNA-binding transcription factor binding | 1.83E-09 | 1.54E-07 | 8.71E-08 | 12 |
| MF | GO:0061629 | RNA polymerase II-specific DNA-binding transcription factor binding | 1.97E-09 | 1.54E-07 | 8.71E-08 | 11 |
| MF | GO:0019902 | phosphatase binding | 1.15E-08 | 7.16E-07 | 4.06E-07 | 9 |
| MF | GO:0005178 | integrin binding | 1.43E-08 | 7.44E-07 | 4.21E-07 | 8 |
| MF | GO:0004674 | protein serine/threonine kinase activity | 2.23E-08 | 9.93E-07 | 5.63E-07 | 12 |
| MF | GO:0004713 | protein tyrosine kinase activity | 3.37E-07 | 1.32E-05 | 7.45E-06 | 7 |
| MF | GO:0031625 | ubiquitin protein ligase binding | 5.35E-07 | 1.86E-05 | 1.05E-05 | 9 |
| MF | GO:0035258 | steroid hormone receptor binding | 6.69E-07 | 2.09E-05 | 1.18E-05 | 6 |
| MF | GO:0035257 | nuclear hormone receptor binding | 7.92E-07 | 2.25E-05 | 1.27E-05 | 7 |
| MF | GO:0044389 | ubiquitin-like protein ligase binding | 8.85E-07 | 2.30E-05 | 1.30E-05 | 9 |
| MF | GO:0016922 | nuclear receptor binding | 1.92E-06 | 4.39E-05 | 2.49E-05 | 6 |
| MF | GO:0042826 | histone deacetylase binding | 2.02E-06 | 4.39E-05 | 2.49E-05 | 6 |
| MF | GO:0004714 | transmembrane receptor protein tyrosine kinase activity | 2.11E-06 | 4.39E-05 | 2.49E-05 | 5 |
| MF | GO:0002039 | p53 binding | 2.88E-06 | 5.41E-05 | 3.07E-05 | 5 |
| MF | GO:0051427 | hormone receptor binding | 2.95E-06 | 5.41E-05 | 3.07E-05 | 7 |
| MF | GO:0051400 | BH domain binding | 4.37E-06 | 7.10E-05 | 4.03E-05 | 3 |
| MF | GO:0070513 | death domain binding | 4.37E-06 | 7.10E-05 | 4.03E-05 | 3 |
| MF | GO:0048018 | receptor ligand activity | 4.55E-06 | 7.10E-05 | 4.03E-05 | 10 |
| MF | GO:0030546 | signaling receptor activator activity | 4.99E-06 | 7.39E-05 | 4.19E-05 | 10 |
| MF | GO:0005126 | cytokine receptor binding | 5.21E-06 | 7.39E-05 | 4.19E-05 | 8 |
| MF | GO:0019199 | transmembrane receptor protein kinase activity | 7.03E-06 | 9.54E-05 | 5.41E-05 | 5 |
| MF | GO:0042056 | chemoattractant activity | 8.09E-06 | 0.000105 | 5.96E-05 | 4 |
| MF | GO:0005125 | cytokine activity | 9.21E-06 | 0.000115 | 6.52E-05 | 7 |
| MF | GO:0030331 | estrogen receptor binding | 1.21E-05 | 0.000146 | 8.25E-05 | 4 |
| MF | GO:0097153 | cysteine-type endopeptidase activity involved in apoptotic process | 1.64E-05 | 0.000189 | 0.000107 | 3 |
| MF | GO:0008083 | growth factor activity | 1.85E-05 | 0.000206 | 0.000117 | 6 |
| MF | GO:0035173 | histone kinase activity | 2.44E-05 | 0.000262 | 0.000149 | 3 |
| MF | GO:0002020 | protease binding | 7.27E-05 | 0.000756 | 0.000429 | 5 |
| MF | GO:0004709 | MAP kinase kinase kinase activity | 9.11E-05 | 0.000917 | 0.00052 | 3 |
| MF | GO:0070491 | repressing transcription factor binding | 9.79E-05 | 0.000955 | 0.000541 | 4 |
| MF | GO:0097718 | disordered domain specific binding | 0.000188 | 0.001778 | 0.001008 | 3 |
| MF | GO:0033613 | activating transcription factor binding | 0.000197 | 0.001807 | 0.001024 | 4 |
| MF | GO:0001103 | RNA polymerase II repressing transcription factor binding | 0.000225 | 0.002001 | 0.001134 | 3 |
| MF | GO:0050839 | cell adhesion molecule binding | 0.000267 | 0.002256 | 0.001279 | 8 |
| MF | GO:0008201 | heparin binding | 0.000268 | 0.002256 | 0.001279 | 5 |
| MF | GO:0051087 | chaperone binding | 0.000396 | 0.003251 | 0.001843 | 4 |
| MF | GO:0032813 | tumor necrosis factor receptor superfamily binding | 0.000507 | 0.003973 | 0.002252 | 3 |
| MF | GO:0047485 | protein N-terminus binding | 0.000509 | 0.003973 | 0.002252 | 4 |
| MF | GO:0004879 | nuclear receptor activity | 0.00054 | 0.004014 | 0.002275 | 3 |
| MF | GO:0098531 | ligand-activated transcription factor activity | 0.00054 | 0.004014 | 0.002275 | 3 |
| MF | GO:1990841 | promoter-specific chromatin binding | 0.000575 | 0.004122 | 0.002336 | 3 |
| MF | GO:0004175 | endopeptidase activity | 0.000581 | 0.004122 | 0.002336 | 7 |
| MF | GO:0070888 | E-box binding | 0.000648 | 0.004496 | 0.002548 | 3 |
| MF | GO:0031072 | heat shock protein binding | 0.000709 | 0.004809 | 0.002726 | 4 |
| MF | GO:0004707 | MAP kinase activity | 0.001002 | 0.006653 | 0.003771 | 2 |
| MF | GO:0097110 | scaffold protein binding | 0.001053 | 0.006772 | 0.003838 | 3 |
| MF | GO:0005539 | glycosaminoglycan binding | 0.001064 | 0.006772 | 0.003838 | 5 |
| MF | GO:0005161 | platelet-derived growth factor receptor binding | 0.001154 | 0.007094 | 0.004021 | 2 |
| MF | GO:0001085 | RNA polymerase II transcription factor binding | 0.00116 | 0.007094 | 0.004021 | 3 |
| MF | GO:0005123 | death receptor binding | 0.001488 | 0.008928 | 0.00506 | 2 |
| MF | GO:1901681 | sulfur compound binding | 0.001568 | 0.009232 | 0.005233 | 5 |

Note: The first hundred results of BP were showed.

**The information of GO enrichment analysis for module 1.**

| **Ontology** | **ID** | **Description** | **pvalue** | **p.adjust** | **qvalue** | **Count** |
| --- | --- | --- | --- | --- | --- | --- |
| BP | GO:0033002 | muscle cell proliferation | 1.77E-13 | 4.08E-10 | 1.23E-10 | 9 |
| BP | GO:2000377 | regulation of reactive oxygen species metabolic process | 2.75E-12 | 3.18E-09 | 9.58E-10 | 8 |
| BP | GO:0062197 | cellular response to chemical stress | 5.50E-12 | 4.24E-09 | 1.28E-09 | 9 |
| BP | GO:0051896 | regulation of protein kinase B signaling | 1.67E-11 | 9.63E-09 | 2.90E-09 | 8 |
| BP | GO:0043491 | protein kinase B signaling | 3.64E-11 | 1.68E-08 | 5.07E-09 | 8 |
| BP | GO:0006979 | response to oxidative stress | 5.30E-11 | 1.85E-08 | 5.58E-09 | 9 |
| BP | GO:0072593 | reactive oxygen species metabolic process | 5.61E-11 | 1.85E-08 | 5.58E-09 | 8 |
| BP | GO:0048660 | regulation of smooth muscle cell proliferation | 7.92E-11 | 2.12E-08 | 6.38E-09 | 7 |
| BP | GO:0048659 | smooth muscle cell proliferation | 8.61E-11 | 2.12E-08 | 6.38E-09 | 7 |
| BP | GO:0034599 | cellular response to oxidative stress | 9.15E-11 | 2.12E-08 | 6.38E-09 | 8 |
| BP | GO:0048661 | positive regulation of smooth muscle cell proliferation | 2.54E-10 | 5.20E-08 | 1.57E-08 | 6 |
| BP | GO:2000379 | positive regulation of reactive oxygen species metabolic process | 2.70E-10 | 5.20E-08 | 1.57E-08 | 6 |
| BP | GO:0010632 | regulation of epithelial cell migration | 3.54E-09 | 5.87E-07 | 1.77E-07 | 7 |
| BP | GO:0070227 | lymphocyte apoptotic process | 3.56E-09 | 5.87E-07 | 1.77E-07 | 5 |
| BP | GO:0010634 | positive regulation of epithelial cell migration | 6.15E-09 | 9.48E-07 | 2.86E-07 | 6 |
| BP | GO:0051897 | positive regulation of protein kinase B signaling | 7.32E-09 | 1.06E-06 | 3.19E-07 | 6 |
| BP | GO:1904705 | regulation of vascular smooth muscle cell proliferation | 1.03E-08 | 1.32E-06 | 3.98E-07 | 5 |
| BP | GO:1990874 | vascular smooth muscle cell proliferation | 1.03E-08 | 1.32E-06 | 3.98E-07 | 5 |
| BP | GO:0017038 | protein import | 1.23E-08 | 1.50E-06 | 4.52E-07 | 6 |
| BP | GO:0010631 | epithelial cell migration | 1.32E-08 | 1.52E-06 | 4.60E-07 | 7 |
| BP | GO:0090132 | epithelium migration | 1.40E-08 | 1.54E-06 | 4.64E-07 | 7 |
| BP | GO:0090130 | tissue migration | 1.57E-08 | 1.65E-06 | 4.97E-07 | 7 |
| BP | GO:1903426 | regulation of reactive oxygen species biosynthetic process | 2.46E-08 | 2.48E-06 | 7.47E-07 | 5 |
| BP | GO:0071887 | leukocyte apoptotic process | 2.86E-08 | 2.75E-06 | 8.29E-07 | 5 |
| BP | GO:2001233 | regulation of apoptotic signaling pathway | 3.52E-08 | 3.25E-06 | 9.81E-07 | 7 |
| BP | GO:0050673 | epithelial cell proliferation | 5.56E-08 | 4.86E-06 | 1.47E-06 | 7 |
| BP | GO:0045429 | positive regulation of nitric oxide biosynthetic process | 5.68E-08 | 4.86E-06 | 1.47E-06 | 4 |
| BP | GO:1904407 | positive regulation of nitric oxide metabolic process | 6.24E-08 | 5.08E-06 | 1.53E-06 | 4 |
| BP | GO:1903409 | reactive oxygen species biosynthetic process | 6.38E-08 | 5.08E-06 | 1.53E-06 | 5 |
| BP | GO:0052547 | regulation of peptidase activity | 7.34E-08 | 5.65E-06 | 1.70E-06 | 7 |
| BP | GO:0010595 | positive regulation of endothelial cell migration | 8.11E-08 | 6.05E-06 | 1.82E-06 | 5 |
| BP | GO:0001667 | ameboidal-type cell migration | 8.52E-08 | 6.15E-06 | 1.86E-06 | 7 |
| BP | GO:1904707 | positive regulation of vascular smooth muscle cell proliferation | 9.72E-08 | 6.80E-06 | 2.05E-06 | 4 |
| BP | GO:0043542 | endothelial cell migration | 1.02E-07 | 6.95E-06 | 2.10E-06 | 6 |
| BP | GO:0050999 | regulation of nitric-oxide synthase activity | 1.14E-07 | 7.56E-06 | 2.28E-06 | 4 |
| BP | GO:0062013 | positive regulation of small molecule metabolic process | 1.32E-07 | 8.37E-06 | 2.52E-06 | 5 |
| BP | GO:0070228 | regulation of lymphocyte apoptotic process | 1.34E-07 | 8.37E-06 | 2.52E-06 | 4 |
| BP | GO:0097193 | intrinsic apoptotic signaling pathway | 1.40E-07 | 8.37E-06 | 2.52E-06 | 6 |
| BP | GO:0006606 | protein import into nucleus | 1.41E-07 | 8.37E-06 | 2.52E-06 | 5 |
| BP | GO:1903428 | positive regulation of reactive oxygen species biosynthetic process | 1.80E-07 | 1.04E-05 | 3.14E-06 | 4 |
| BP | GO:0009895 | negative regulation of catabolic process | 2.04E-07 | 1.15E-05 | 3.47E-06 | 6 |
| BP | GO:0007568 | aging | 2.60E-07 | 1.43E-05 | 4.32E-06 | 6 |
| BP | GO:0051170 | import into nucleus | 2.71E-07 | 1.46E-05 | 4.39E-06 | 5 |
| BP | GO:2001242 | regulation of intrinsic apoptotic signaling pathway | 2.88E-07 | 1.49E-05 | 4.49E-06 | 5 |
| BP | GO:0046677 | response to antibiotic | 2.90E-07 | 1.49E-05 | 4.49E-06 | 6 |
| BP | GO:0032768 | regulation of monooxygenase activity | 3.08E-07 | 1.55E-05 | 4.66E-06 | 4 |
| BP | GO:0034614 | cellular response to reactive oxygen species | 3.15E-07 | 1.55E-05 | 4.67E-06 | 5 |
| BP | GO:0045428 | regulation of nitric oxide biosynthetic process | 3.27E-07 | 1.58E-05 | 4.75E-06 | 4 |
| BP | GO:0001936 | regulation of endothelial cell proliferation | 3.97E-07 | 1.87E-05 | 5.65E-06 | 5 |
| BP | GO:0045862 | positive regulation of proteolysis | 4.94E-07 | 2.26E-05 | 6.81E-06 | 6 |
| BP | GO:0001666 | response to hypoxia | 5.02E-07 | 2.26E-05 | 6.81E-06 | 6 |
| BP | GO:1903708 | positive regulation of hemopoiesis | 5.08E-07 | 2.26E-05 | 6.81E-06 | 5 |
| BP | GO:0018108 | peptidyl-tyrosine phosphorylation | 5.35E-07 | 2.33E-05 | 7.04E-06 | 6 |
| BP | GO:0018212 | peptidyl-tyrosine modification | 5.62E-07 | 2.40E-05 | 7.25E-06 | 6 |
| BP | GO:0001935 | endothelial cell proliferation | 5.96E-07 | 2.43E-05 | 7.34E-06 | 5 |
| BP | GO:0036293 | response to decreased oxygen levels | 5.99E-07 | 2.43E-05 | 7.34E-06 | 6 |
| BP | GO:0006809 | nitric oxide biosynthetic process | 6.11E-07 | 2.43E-05 | 7.34E-06 | 4 |
| BP | GO:0007565 | female pregnancy | 6.11E-07 | 2.43E-05 | 7.34E-06 | 5 |
| BP | GO:0050727 | regulation of inflammatory response | 6.38E-07 | 2.50E-05 | 7.53E-06 | 6 |
| BP | GO:0043536 | positive regulation of blood vessel endothelial cell migration | 6.78E-07 | 2.57E-05 | 7.75E-06 | 4 |
| BP | GO:0050678 | regulation of epithelial cell proliferation | 6.78E-07 | 2.57E-05 | 7.75E-06 | 6 |
| BP | GO:0014855 | striated muscle cell proliferation | 7.50E-07 | 2.79E-05 | 8.42E-06 | 4 |
| BP | GO:0046209 | nitric oxide metabolic process | 7.88E-07 | 2.89E-05 | 8.71E-06 | 4 |
| BP | GO:0035265 | organ growth | 8.25E-07 | 2.94E-05 | 8.87E-06 | 5 |
| BP | GO:2000106 | regulation of leukocyte apoptotic process | 8.27E-07 | 2.94E-05 | 8.87E-06 | 4 |
| BP | GO:0070482 | response to oxygen levels | 8.64E-07 | 2.99E-05 | 9.03E-06 | 6 |
| BP | GO:0010507 | negative regulation of autophagy | 8.68E-07 | 2.99E-05 | 9.03E-06 | 4 |
| BP | GO:0071456 | cellular response to hypoxia | 8.86E-07 | 3.01E-05 | 9.08E-06 | 5 |
| BP | GO:2001057 | reactive nitrogen species metabolic process | 9.10E-07 | 3.03E-05 | 9.13E-06 | 4 |
| BP | GO:0001503 | ossification | 9.17E-07 | 3.03E-05 | 9.13E-06 | 6 |
| BP | GO:0051000 | positive regulation of nitric-oxide synthase activity | 9.55E-07 | 3.11E-05 | 9.38E-06 | 3 |
| BP | GO:1900407 | regulation of cellular response to oxidative stress | 1.05E-06 | 3.36E-05 | 1.01E-05 | 4 |
| BP | GO:0043281 | regulation of cysteine-type endopeptidase activity involved in apoptotic process | 1.07E-06 | 3.38E-05 | 1.02E-05 | 5 |
| BP | GO:0036294 | cellular response to decreased oxygen levels | 1.12E-06 | 3.49E-05 | 1.05E-05 | 5 |
| BP | GO:0045639 | positive regulation of myeloid cell differentiation | 1.20E-06 | 3.69E-05 | 1.11E-05 | 4 |
| BP | GO:0044706 | multi-multicellular organism process | 1.25E-06 | 3.81E-05 | 1.15E-05 | 5 |
| BP | GO:0052548 | regulation of endopeptidase activity | 1.34E-06 | 4.03E-05 | 1.22E-05 | 6 |
| BP | GO:0062012 | regulation of small molecule metabolic process | 1.42E-06 | 4.13E-05 | 1.24E-05 | 6 |
| BP | GO:0009299 | mRNA transcription | 1.42E-06 | 4.13E-05 | 1.24E-05 | 3 |
| BP | GO:0048608 | reproductive structure development | 1.46E-06 | 4.13E-05 | 1.24E-05 | 6 |
| BP | GO:0010594 | regulation of endothelial cell migration | 1.46E-06 | 4.13E-05 | 1.24E-05 | 5 |
| BP | GO:0051090 | regulation of DNA-binding transcription factor activity | 1.48E-06 | 4.13E-05 | 1.24E-05 | 6 |
| BP | GO:2001234 | negative regulation of apoptotic signaling pathway | 1.49E-06 | 4.13E-05 | 1.24E-05 | 5 |
| BP | GO:0048732 | gland development | 1.52E-06 | 4.13E-05 | 1.24E-05 | 6 |
| BP | GO:0061458 | reproductive system development | 1.52E-06 | 4.13E-05 | 1.24E-05 | 6 |
| BP | GO:1902882 | regulation of response to oxidative stress | 1.55E-06 | 4.13E-05 | 1.25E-05 | 4 |
| BP | GO:0000302 | response to reactive oxygen species | 1.55E-06 | 4.13E-05 | 1.25E-05 | 5 |
| BP | GO:2001243 | negative regulation of intrinsic apoptotic signaling pathway | 1.61E-06 | 4.21E-05 | 1.27E-05 | 4 |
| BP | GO:0071453 | cellular response to oxygen levels | 1.62E-06 | 4.21E-05 | 1.27E-05 | 5 |
| BP | GO:0051222 | positive regulation of protein transport | 1.64E-06 | 4.22E-05 | 1.27E-05 | 6 |
| BP | GO:2000116 | regulation of cysteine-type endopeptidase activity | 1.80E-06 | 4.57E-05 | 1.38E-05 | 5 |
| BP | GO:0031334 | positive regulation of protein-containing complex assembly | 1.99E-06 | 5.00E-05 | 1.51E-05 | 5 |
| BP | GO:1904951 | positive regulation of establishment of protein localization | 2.02E-06 | 5.03E-05 | 1.52E-05 | 6 |
| BP | GO:0048872 | homeostasis of number of cells | 2.07E-06 | 5.10E-05 | 1.54E-05 | 5 |
| BP | GO:0033138 | positive regulation of peptidyl-serine phosphorylation | 2.12E-06 | 5.17E-05 | 1.56E-05 | 4 |
| BP | GO:0042136 | neurotransmitter biosynthetic process | 2.21E-06 | 5.31E-05 | 1.60E-05 | 4 |
| BP | GO:0001819 | positive regulation of cytokine production | 2.24E-06 | 5.33E-05 | 1.61E-05 | 6 |
| BP | GO:0051341 | regulation of oxidoreductase activity | 2.29E-06 | 5.40E-05 | 1.63E-05 | 4 |
| BP | GO:0031330 | negative regulation of cellular catabolic process | 2.38E-06 | 5.55E-05 | 1.67E-05 | 5 |
| BP | GO:0001938 | positive regulation of endothelial cell proliferation | 2.75E-06 | 6.17E-05 | 1.86E-05 | 4 |
| CC | GO:0101002 | ficolin-1-rich granule | 0.000155 | 0.00712 | 0.004562 | 3 |
| CC | GO:1904813 | ficolin-1-rich granule lumen | 0.000155 | 0.00712 | 0.004562 | 3 |
| CC | GO:0000790 | nuclear chromatin | 0.000254 | 0.007803 | 0.004999 | 4 |
| CC | GO:0090575 | RNA polymerase II transcription regulator complex | 0.000365 | 0.008404 | 0.005385 | 3 |
| MF | GO:0019903 | protein phosphatase binding | 1.65E-07 | 2.43E-05 | 8.18E-06 | 5 |
| MF | GO:0019902 | phosphatase binding | 6.61E-07 | 4.86E-05 | 1.64E-05 | 5 |
| MF | GO:1990782 | protein tyrosine kinase binding | 9.06E-05 | 0.004003 | 0.001347 | 3 |
| MF | GO:0061629 | RNA polymerase II-specific DNA-binding transcription factor binding | 0.000119 | 0.004003 | 0.001347 | 4 |
| MF | GO:0042826 | histone deacetylase binding | 0.000153 | 0.004003 | 0.001347 | 3 |
| MF | GO:0001091 | RNA polymerase II general transcription initiation factor binding | 0.000163 | 0.004003 | 0.001347 | 2 |
| MF | GO:0002020 | protease binding | 0.000233 | 0.004821 | 0.001622 | 3 |
| MF | GO:0070851 | growth factor receptor binding | 0.000267 | 0.004821 | 0.001622 | 3 |
| MF | GO:0140297 | DNA-binding transcription factor binding | 0.000295 | 0.004821 | 0.001622 | 4 |
| MF | GO:0035035 | histone acetyltransferase binding | 0.000347 | 0.005096 | 0.001715 | 2 |
| MF | GO:0035257 | nuclear hormone receptor binding | 0.000387 | 0.005096 | 0.001715 | 3 |
| MF | GO:0051721 | protein phosphatase 2A binding | 0.000424 | 0.005096 | 0.001715 | 2 |
| MF | GO:0097718 | disordered domain specific binding | 0.000451 | 0.005096 | 0.001715 | 2 |
| MF | GO:0051427 | hormone receptor binding | 0.000686 | 0.006259 | 0.002107 | 3 |
| MF | GO:0001228 | DNA-binding transcription activator activity, RNA polymerase II-specific | 0.000688 | 0.006259 | 0.002107 | 4 |
| MF | GO:0001216 | DNA-binding transcription activator activity | 0.000694 | 0.006259 | 0.002107 | 4 |
| MF | GO:0140296 | general transcription initiation factor binding | 0.000731 | 0.006259 | 0.002107 | 2 |
| MF | GO:0004712 | protein serine/threonine/tyrosine kinase activity | 0.000766 | 0.006259 | 0.002107 | 2 |
| MF | GO:0004879 | nuclear receptor activity | 0.000915 | 0.006728 | 0.002264 | 2 |
| MF | GO:0098531 | ligand-activated transcription factor activity | 0.000915 | 0.006728 | 0.002264 | 2 |
| MF | GO:0005125 | cytokine activity | 0.001133 | 0.007932 | 0.00267 | 3 |

Note: The first hundred results of BP were showed.

**The information of GO enrichment analysis for module 2.**

| **Ontology** | **ID** | **Description** | **pvalue** | **p.adjust** | **qvalue** | **Count** |
| --- | --- | --- | --- | --- | --- | --- |
| BP | GO:0038034 | signal transduction in absence of ligand | 5.90E-14 | 5.33E-11 | 1.84E-11 | 7 |
| BP | GO:0097192 | extrinsic apoptotic signaling pathway in absence of ligand | 5.90E-14 | 5.33E-11 | 1.84E-11 | 7 |
| BP | GO:0097191 | extrinsic apoptotic signaling pathway | 2.27E-12 | 1.36E-09 | 4.71E-10 | 8 |
| BP | GO:1901216 | positive regulation of neuron death | 6.69E-11 | 3.02E-08 | 1.04E-08 | 6 |
| BP | GO:2001233 | regulation of apoptotic signaling pathway | 2.63E-10 | 9.49E-08 | 3.28E-08 | 8 |
| BP | GO:1901214 | regulation of neuron death | 2.00E-09 | 6.01E-07 | 2.07E-07 | 7 |
| BP | GO:2001235 | positive regulation of apoptotic signaling pathway | 3.33E-09 | 8.58E-07 | 2.96E-07 | 6 |
| BP | GO:0070997 | neuron death | 4.17E-09 | 9.40E-07 | 3.25E-07 | 7 |
| BP | GO:2001234 | negative regulation of apoptotic signaling pathway | 1.49E-08 | 3.00E-06 | 1.03E-06 | 6 |
| BP | GO:0008637 | apoptotic mitochondrial changes | 3.39E-08 | 6.12E-06 | 2.11E-06 | 5 |
| BP | GO:2001239 | regulation of extrinsic apoptotic signaling pathway in absence of ligand | 4.71E-08 | 7.74E-06 | 2.67E-06 | 4 |
| BP | GO:0018105 | peptidyl-serine phosphorylation | 7.11E-08 | 1.07E-05 | 3.69E-06 | 6 |
| BP | GO:2001236 | regulation of extrinsic apoptotic signaling pathway | 1.04E-07 | 1.23E-05 | 4.26E-06 | 5 |
| BP | GO:0043525 | positive regulation of neuron apoptotic process | 1.04E-07 | 1.23E-05 | 4.26E-06 | 4 |
| BP | GO:0043010 | camera-type eye development | 1.04E-07 | 1.23E-05 | 4.26E-06 | 6 |
| BP | GO:0018209 | peptidyl-serine modification | 1.10E-07 | 1.23E-05 | 4.26E-06 | 6 |
| BP | GO:0061029 | eyelid development in camera-type eye | 1.19E-07 | 1.23E-05 | 4.26E-06 | 3 |
| BP | GO:0071214 | cellular response to abiotic stimulus | 1.30E-07 | 1.23E-05 | 4.26E-06 | 6 |
| BP | GO:0104004 | cellular response to environmental stimulus | 1.30E-07 | 1.23E-05 | 4.26E-06 | 6 |
| BP | GO:0045862 | positive regulation of proteolysis | 2.06E-07 | 1.86E-05 | 6.42E-06 | 6 |
| BP | GO:0001654 | eye development | 2.20E-07 | 1.89E-05 | 6.53E-06 | 6 |
| BP | GO:0150063 | visual system development | 2.35E-07 | 1.93E-05 | 6.65E-06 | 6 |
| BP | GO:0048880 | sensory system development | 2.54E-07 | 2.00E-05 | 6.89E-06 | 6 |
| BP | GO:0150076 | neuroinflammatory response | 3.17E-07 | 2.38E-05 | 8.23E-06 | 4 |
| BP | GO:0046902 | regulation of mitochondrial membrane permeability | 3.34E-07 | 2.41E-05 | 8.34E-06 | 4 |
| BP | GO:0043523 | regulation of neuron apoptotic process | 4.70E-07 | 3.27E-05 | 1.13E-05 | 5 |
| BP | GO:0030099 | myeloid cell differentiation | 4.98E-07 | 3.33E-05 | 1.15E-05 | 6 |
| BP | GO:0090559 | regulation of membrane permeability | 5.51E-07 | 3.55E-05 | 1.23E-05 | 4 |
| BP | GO:0048608 | reproductive structure development | 6.13E-07 | 3.82E-05 | 1.32E-05 | 6 |
| BP | GO:0061458 | reproductive system development | 6.39E-07 | 3.84E-05 | 1.33E-05 | 6 |
| BP | GO:0046777 | protein autophosphorylation | 8.20E-07 | 4.78E-05 | 1.65E-05 | 5 |
| BP | GO:0051402 | neuron apoptotic process | 8.92E-07 | 5.03E-05 | 1.74E-05 | 5 |
| BP | GO:0051817 | modulation of process of other organism involved in symbiotic interaction | 9.71E-07 | 5.31E-05 | 1.83E-05 | 4 |
| BP | GO:0008630 | intrinsic apoptotic signaling pathway in response to DNA damage | 1.18E-06 | 6.10E-05 | 2.11E-05 | 4 |
| BP | GO:2001237 | negative regulation of extrinsic apoptotic signaling pathway | 1.18E-06 | 6.10E-05 | 2.11E-05 | 4 |
| BP | GO:0035821 | modulation of process of other organism | 1.65E-06 | 8.27E-05 | 2.86E-05 | 4 |
| BP | GO:0097193 | intrinsic apoptotic signaling pathway | 2.27E-06 | 0.000111 | 3.82E-05 | 5 |
| BP | GO:1901030 | positive regulation of mitochondrial outer membrane permeabilization involved in apoptotic signaling pathway | 2.70E-06 | 0.000128 | 4.43E-05 | 3 |
| BP | GO:0045740 | positive regulation of DNA replication | 2.95E-06 | 0.00013 | 4.48E-05 | 3 |
| BP | GO:1901099 | negative regulation of signal transduction in absence of ligand | 2.95E-06 | 0.00013 | 4.48E-05 | 3 |
| BP | GO:2001240 | negative regulation of extrinsic apoptotic signaling pathway in absence of ligand | 2.95E-06 | 0.00013 | 4.48E-05 | 3 |
| BP | GO:0038083 | peptidyl-tyrosine autophosphorylation | 3.21E-06 | 0.000137 | 4.72E-05 | 3 |
| BP | GO:0007006 | mitochondrial membrane organization | 3.26E-06 | 0.000137 | 4.72E-05 | 4 |
| BP | GO:0033135 | regulation of peptidyl-serine phosphorylation | 3.77E-06 | 0.000155 | 5.34E-05 | 4 |
| BP | GO:0032147 | activation of protein kinase activity | 4.54E-06 | 0.000182 | 6.29E-05 | 5 |
| BP | GO:1901028 | regulation of mitochondrial outer membrane permeabilization involved in apoptotic signaling pathway | 5.45E-06 | 0.000214 | 7.38E-05 | 3 |
| BP | GO:0031331 | positive regulation of cellular catabolic process | 6.82E-06 | 0.00026 | 8.98E-05 | 5 |
| BP | GO:0018108 | peptidyl-tyrosine phosphorylation | 6.91E-06 | 0.00026 | 8.98E-05 | 5 |
| BP | GO:0018212 | peptidyl-tyrosine modification | 7.20E-06 | 0.000265 | 9.15E-05 | 5 |
| BP | GO:2001242 | regulation of intrinsic apoptotic signaling pathway | 7.44E-06 | 0.000269 | 9.28E-05 | 4 |
| BP | GO:0021543 | pallium development | 8.18E-06 | 0.00029 | 1.00E-04 | 4 |
| BP | GO:0097345 | mitochondrial outer membrane permeabilization | 1.02E-05 | 0.00035 | 0.000121 | 3 |
| BP | GO:0010821 | regulation of mitochondrion organization | 1.03E-05 | 0.00035 | 0.000121 | 4 |
| BP | GO:0001541 | ovarian follicle development | 1.07E-05 | 0.000359 | 0.000124 | 3 |
| BP | GO:0050770 | regulation of axonogenesis | 1.12E-05 | 0.000368 | 0.000127 | 4 |
| BP | GO:0045785 | positive regulation of cell adhesion | 1.15E-05 | 0.00037 | 0.000128 | 5 |
| BP | GO:0061900 | glial cell activation | 1.26E-05 | 0.000399 | 0.000138 | 3 |
| BP | GO:0051054 | positive regulation of DNA metabolic process | 1.33E-05 | 0.000406 | 0.00014 | 4 |
| BP | GO:0001836 | release of cytochrome c from mitochondria | 1.33E-05 | 0.000406 | 0.00014 | 3 |
| BP | GO:1902110 | positive regulation of mitochondrial membrane permeability involved in apoptotic process | 1.40E-05 | 0.00042 | 0.000145 | 3 |
| BP | GO:2001244 | positive regulation of intrinsic apoptotic signaling pathway | 1.47E-05 | 0.000425 | 0.000147 | 3 |
| BP | GO:0009896 | positive regulation of catabolic process | 1.49E-05 | 0.000425 | 0.000147 | 5 |
| BP | GO:0052548 | regulation of endopeptidase activity | 1.49E-05 | 0.000425 | 0.000147 | 5 |
| BP | GO:0051205 | protein insertion into membrane | 1.54E-05 | 0.000428 | 0.000148 | 3 |
| BP | GO:1902686 | mitochondrial outer membrane permeabilization involved in programmed cell death | 1.54E-05 | 0.000428 | 0.000148 | 3 |
| BP | GO:0035690 | cellular response to drug | 1.62E-05 | 0.000444 | 0.000153 | 5 |
| BP | GO:0035794 | positive regulation of mitochondrial membrane permeability | 1.70E-05 | 0.000456 | 0.000158 | 3 |
| BP | GO:0002573 | myeloid leukocyte differentiation | 1.72E-05 | 0.000456 | 0.000158 | 4 |
| BP | GO:0007623 | circadian rhythm | 1.86E-05 | 0.000464 | 0.00016 | 4 |
| BP | GO:1901215 | negative regulation of neuron death | 1.86E-05 | 0.000464 | 0.00016 | 4 |
| BP | GO:1902108 | regulation of mitochondrial membrane permeability involved in apoptotic process | 1.86E-05 | 0.000464 | 0.00016 | 3 |
| BP | GO:1905710 | positive regulation of membrane permeability | 1.86E-05 | 0.000464 | 0.00016 | 3 |
| BP | GO:0071229 | cellular response to acid chemical | 1.89E-05 | 0.000464 | 0.00016 | 4 |
| BP | GO:0009314 | response to radiation | 1.92E-05 | 0.000464 | 0.00016 | 5 |
| BP | GO:0009612 | response to mechanical stimulus | 1.93E-05 | 0.000464 | 0.00016 | 4 |
| BP | GO:0052547 | regulation of peptidase activity | 2.00E-05 | 0.000475 | 0.000164 | 5 |
| BP | GO:2001020 | regulation of response to DNA damage stimulus | 2.07E-05 | 0.000486 | 0.000168 | 4 |
| BP | GO:0043281 | regulation of cysteine-type endopeptidase activity involved in apoptotic process | 2.11E-05 | 0.000489 | 0.000169 | 4 |
| BP | GO:0001667 | ameboidal-type cell migration | 2.22E-05 | 0.000507 | 0.000175 | 5 |
| BP | GO:0010749 | regulation of nitric oxide mediated signal transduction | 2.70E-05 | 0.000602 | 0.000208 | 2 |
| BP | GO:0032025 | response to cobalt ion | 2.70E-05 | 0.000602 | 0.000208 | 2 |
| BP | GO:0048871 | multicellular organismal homeostasis | 2.83E-05 | 0.000624 | 0.000215 | 5 |
| BP | GO:0071260 | cellular response to mechanical stimulus | 3.20E-05 | 0.000687 | 0.000237 | 3 |
| BP | GO:2000116 | regulation of cysteine-type endopeptidase activity | 3.20E-05 | 0.000687 | 0.000237 | 4 |
| BP | GO:2001269 | positive regulation of cysteine-type endopeptidase activity involved in apoptotic signaling pathway | 3.30E-05 | 0.000701 | 0.000242 | 2 |
| BP | GO:0048145 | regulation of fibroblast proliferation | 3.71E-05 | 0.000778 | 0.000269 | 3 |
| BP | GO:0021537 | telencephalon development | 3.76E-05 | 0.000779 | 0.000269 | 4 |
| BP | GO:0048144 | fibroblast proliferation | 3.84E-05 | 0.000786 | 0.000271 | 3 |
| BP | GO:0045637 | regulation of myeloid cell differentiation | 3.87E-05 | 0.000786 | 0.000271 | 4 |
| BP | GO:0010623 | programmed cell death involved in cell development | 3.96E-05 | 0.000794 | 0.000274 | 2 |
| BP | GO:0006919 | activation of cysteine-type endopeptidase activity involved in apoptotic process | 4.13E-05 | 0.000809 | 0.000279 | 3 |
| BP | GO:0008625 | extrinsic apoptotic signaling pathway via death domain receptors | 4.13E-05 | 0.000809 | 0.000279 | 3 |
| BP | GO:0006839 | mitochondrial transport | 4.31E-05 | 0.000828 | 0.000286 | 4 |
| BP | GO:0043406 | positive regulation of MAP kinase activity | 4.31E-05 | 0.000828 | 0.000286 | 4 |
| BP | GO:0097194 | execution phase of apoptosis | 4.42E-05 | 0.00084 | 0.00029 | 3 |
| BP | GO:0007494 | midgut development | 4.68E-05 | 0.000861 | 0.000297 | 2 |
| BP | GO:1901550 | regulation of endothelial cell development | 4.68E-05 | 0.000861 | 0.000297 | 2 |
| BP | GO:1903140 | regulation of establishment of endothelial barrier | 4.68E-05 | 0.000861 | 0.000297 | 2 |
| BP | GO:0043491 | protein kinase B signaling | 5.08E-05 | 0.000925 | 0.00032 | 4 |
| BP | GO:0043922 | negative regulation by host of viral transcription | 5.45E-05 | 0.000984 | 0.00034 | 2 |
| CC | GO:0005741 | mitochondrial outer membrane | 8.11E-06 | 0.000363 | 0.000215 | 4 |
| CC | GO:0031968 | organelle outer membrane | 1.31E-05 | 0.000363 | 0.000215 | 4 |
| CC | GO:0019867 | outer membrane | 1.36E-05 | 0.000363 | 0.000215 | 4 |
| MF | GO:0051400 | BH domain binding | 5.89E-08 | 3.15E-06 | 1.09E-06 | 3 |
| MF | GO:0070513 | death domain binding | 5.89E-08 | 3.15E-06 | 1.09E-06 | 3 |
| MF | GO:0097153 | cysteine-type endopeptidase activity involved in apoptotic process | 7.00E-05 | 0.00179 | 0.000616 | 2 |
| MF | GO:0005126 | cytokine receptor binding | 7.92E-05 | 0.00179 | 0.000616 | 4 |
| MF | GO:0031625 | ubiquitin protein ligase binding | 8.36E-05 | 0.00179 | 0.000616 | 4 |
| MF | GO:0044389 | ubiquitin-like protein ligase binding | 0.000106 | 0.001883 | 0.000648 | 4 |
| MF | GO:0004713 | protein tyrosine kinase activity | 0.000181 | 0.002417 | 0.000832 | 3 |
| MF | GO:0070851 | growth factor receptor binding | 0.000181 | 0.002417 | 0.000832 | 3 |
| MF | GO:0051059 | NF-kappaB binding | 0.000269 | 0.003195 | 0.0011 | 2 |
| MF | GO:0005125 | cytokine activity | 0.000772 | 0.008263 | 0.002845 | 3 |

Note: The first hundred results of BP were showed.

**The information of GO enrichment analysis for module 3.**

| **Ontology** | **ID** | **Description** | **pvalue** | **p.adjust** | **qvalue** | **Count** |
| --- | --- | --- | --- | --- | --- | --- |
| BP | GO:0062197 | cellular response to chemical stress | 8.20E-09 | 7.27E-06 | 2.41E-06 | 6 |
| BP | GO:0051098 | regulation of binding | 1.20E-08 | 7.27E-06 | 2.41E-06 | 6 |
| BP | GO:0030099 | myeloid cell differentiation | 2.30E-08 | 9.29E-06 | 3.08E-06 | 6 |
| BP | GO:0002573 | myeloid leukocyte differentiation | 3.57E-08 | 1.08E-05 | 3.59E-06 | 5 |
| BP | GO:0034349 | glial cell apoptotic process | 5.02E-08 | 1.22E-05 | 4.04E-06 | 3 |
| BP | GO:0051101 | regulation of DNA binding | 3.77E-07 | 7.62E-05 | 2.53E-05 | 4 |
| BP | GO:0033044 | regulation of chromosome organization | 4.68E-07 | 8.11E-05 | 2.69E-05 | 5 |
| BP | GO:0048545 | response to steroid hormone | 8.20E-07 | 0.000124 | 4.12E-05 | 5 |
| BP | GO:0070482 | response to oxygen levels | 9.43E-07 | 0.000127 | 4.21E-05 | 5 |
| BP | GO:2001233 | regulation of apoptotic signaling pathway | 1.09E-06 | 0.000132 | 4.39E-05 | 5 |
| BP | GO:0030225 | macrophage differentiation | 1.45E-06 | 0.000148 | 4.92E-05 | 3 |
| BP | GO:0060249 | anatomical structure homeostasis | 1.61E-06 | 0.000148 | 4.92E-05 | 5 |
| BP | GO:0051099 | positive regulation of binding | 1.64E-06 | 0.000148 | 4.92E-05 | 4 |
| BP | GO:0043401 | steroid hormone mediated signaling pathway | 1.75E-06 | 0.000148 | 4.92E-05 | 4 |
| BP | GO:0006979 | response to oxidative stress | 1.83E-06 | 0.000148 | 4.92E-05 | 5 |
| BP | GO:0043388 | positive regulation of DNA binding | 3.54E-06 | 0.000268 | 8.90E-05 | 3 |
| BP | GO:0000302 | response to reactive oxygen species | 4.60E-06 | 0.000304 | 0.000101 | 4 |
| BP | GO:0009755 | hormone-mediated signaling pathway | 4.68E-06 | 0.000304 | 0.000101 | 4 |
| BP | GO:0071453 | cellular response to oxygen levels | 4.76E-06 | 0.000304 | 0.000101 | 4 |
| BP | GO:0051402 | neuron apoptotic process | 5.18E-06 | 0.000314 | 0.000104 | 4 |
| BP | GO:0071383 | cellular response to steroid hormone stimulus | 5.99E-06 | 0.000346 | 0.000115 | 4 |
| BP | GO:1900182 | positive regulation of protein localization to nucleus | 7.03E-06 | 0.000387 | 0.000129 | 3 |
| BP | GO:0030522 | intracellular receptor signaling pathway | 9.41E-06 | 0.000479 | 0.000159 | 4 |
| BP | GO:0010833 | telomere maintenance via telomere lengthening | 9.58E-06 | 0.000479 | 0.000159 | 3 |
| BP | GO:0051348 | negative regulation of transferase activity | 1.04E-05 | 0.000479 | 0.000159 | 4 |
| BP | GO:1904705 | regulation of vascular smooth muscle cell proliferation | 1.07E-05 | 0.000479 | 0.000159 | 3 |
| BP | GO:1990874 | vascular smooth muscle cell proliferation | 1.07E-05 | 0.000479 | 0.000159 | 3 |
| BP | GO:0048511 | rhythmic process | 1.19E-05 | 0.000515 | 0.000171 | 4 |
| BP | GO:0034599 | cellular response to oxidative stress | 1.31E-05 | 0.000546 | 0.000181 | 4 |
| BP | GO:0051974 | negative regulation of telomerase activity | 1.42E-05 | 0.000572 | 0.00019 | 2 |
| BP | GO:0071375 | cellular response to peptide hormone stimulus | 1.66E-05 | 0.000632 | 0.00021 | 4 |
| BP | GO:0018209 | peptidyl-serine modification | 1.68E-05 | 0.000632 | 0.00021 | 4 |
| BP | GO:1903829 | positive regulation of cellular protein localization | 1.72E-05 | 0.000632 | 0.00021 | 4 |
| BP | GO:0000079 | regulation of cyclin-dependent protein serine/threonine kinase activity | 1.85E-05 | 0.000641 | 0.000213 | 3 |
| BP | GO:0032496 | response to lipopolysaccharide | 1.85E-05 | 0.000641 | 0.000213 | 4 |
| BP | GO:1904029 | regulation of cyclin-dependent protein kinase activity | 2.07E-05 | 0.000687 | 0.000228 | 3 |
| BP | GO:0001101 | response to acid chemical | 2.15E-05 | 0.000687 | 0.000228 | 4 |
| BP | GO:0002237 | response to molecule of bacterial origin | 2.15E-05 | 0.000687 | 0.000228 | 4 |
| BP | GO:0070997 | neuron death | 2.28E-05 | 0.000709 | 0.000235 | 4 |
| BP | GO:0045651 | positive regulation of macrophage differentiation | 2.34E-05 | 0.00071 | 0.000235 | 2 |
| BP | GO:0043279 | response to alkaloid | 2.45E-05 | 0.000723 | 0.00024 | 3 |
| BP | GO:0001666 | response to hypoxia | 2.58E-05 | 0.000743 | 0.000247 | 4 |
| BP | GO:0010038 | response to metal ion | 2.66E-05 | 0.000748 | 0.000248 | 4 |
| BP | GO:1900180 | regulation of protein localization to nucleus | 2.72E-05 | 0.000748 | 0.000248 | 3 |
| BP | GO:0036293 | response to decreased oxygen levels | 2.90E-05 | 0.000781 | 0.000259 | 4 |
| BP | GO:0051053 | negative regulation of DNA metabolic process | 3.24E-05 | 0.000853 | 0.000283 | 3 |
| BP | GO:1901653 | cellular response to peptide | 3.39E-05 | 0.000874 | 0.00029 | 4 |
| BP | GO:0045787 | positive regulation of cell cycle | 3.53E-05 | 0.000891 | 0.000296 | 4 |
| BP | GO:0030518 | intracellular steroid hormone receptor signaling pathway | 3.82E-05 | 0.000945 | 0.000313 | 3 |
| BP | GO:0060149 | negative regulation of posttranscriptional gene silencing | 4.39E-05 | 0.001005 | 0.000333 | 2 |
| BP | GO:0060967 | negative regulation of gene silencing by RNA | 4.39E-05 | 0.001005 | 0.000333 | 2 |
| BP | GO:1905288 | vascular associated smooth muscle cell apoptotic process | 4.39E-05 | 0.001005 | 0.000333 | 2 |
| BP | GO:1905459 | regulation of vascular associated smooth muscle cell apoptotic process | 4.39E-05 | 0.001005 | 0.000333 | 2 |
| BP | GO:1901342 | regulation of vasculature development | 4.85E-05 | 0.001073 | 0.000356 | 4 |
| BP | GO:0009411 | response to UV | 4.87E-05 | 0.001073 | 0.000356 | 3 |
| BP | GO:0036120 | cellular response to platelet-derived growth factor stimulus | 5.39E-05 | 0.001128 | 0.000374 | 2 |
| BP | GO:0051900 | regulation of mitochondrial depolarization | 5.39E-05 | 0.001128 | 0.000374 | 2 |
| BP | GO:2001251 | negative regulation of chromosome organization | 5.40E-05 | 0.001128 | 0.000374 | 3 |
| BP | GO:0043434 | response to peptide hormone | 5.51E-05 | 0.001132 | 0.000376 | 4 |
| BP | GO:0036119 | response to platelet-derived growth factor | 5.93E-05 | 0.001178 | 0.000391 | 2 |
| BP | GO:0045649 | regulation of macrophage differentiation | 5.93E-05 | 0.001178 | 0.000391 | 2 |
| BP | GO:0016570 | histone modification | 6.45E-05 | 0.001249 | 0.000414 | 4 |
| BP | GO:0051882 | mitochondrial depolarization | 6.49E-05 | 0.001249 | 0.000414 | 2 |
| BP | GO:0000723 | telomere maintenance | 7.36E-05 | 0.001372 | 0.000455 | 3 |
| BP | GO:0031960 | response to corticosteroid | 7.36E-05 | 0.001372 | 0.000455 | 3 |
| BP | GO:0045931 | positive regulation of mitotic cell cycle | 7.49E-05 | 0.001372 | 0.000455 | 3 |
| BP | GO:0016569 | covalent chromatin modification | 7.63E-05 | 0.001372 | 0.000455 | 4 |
| BP | GO:2000679 | positive regulation of transcription regulatory region DNA binding | 7.70E-05 | 0.001372 | 0.000455 | 2 |
| BP | GO:0034614 | cellular response to reactive oxygen species | 8.20E-05 | 0.00144 | 0.000478 | 3 |
| BP | GO:0048660 | regulation of smooth muscle cell proliferation | 8.34E-05 | 0.001445 | 0.000479 | 3 |
| BP | GO:0048659 | smooth muscle cell proliferation | 8.64E-05 | 0.001475 | 0.000489 | 3 |
| BP | GO:1904357 | negative regulation of telomere maintenance via telomere lengthening | 9.00E-05 | 0.001515 | 0.000503 | 2 |
| BP | GO:0032200 | telomere organization | 9.26E-05 | 0.001537 | 0.00051 | 3 |
| BP | GO:1901654 | response to ketone | 0.000124 | 0.002025 | 0.000672 | 3 |
| BP | GO:0038128 | ERBB2 signaling pathway | 0.000127 | 0.002025 | 0.000672 | 2 |
| BP | GO:0051385 | response to mineralocorticoid | 0.000127 | 0.002025 | 0.000672 | 2 |
| BP | GO:0071897 | DNA biosynthetic process | 0.00013 | 0.002038 | 0.000676 | 3 |
| BP | GO:0048011 | neurotrophin TRK receptor signaling pathway | 0.000135 | 0.0021 | 0.000697 | 2 |
| BP | GO:0060969 | negative regulation of gene silencing | 0.000144 | 0.002202 | 0.000731 | 2 |
| BP | GO:0071222 | cellular response to lipopolysaccharide | 0.000148 | 0.002219 | 0.000736 | 3 |
| BP | GO:0071312 | cellular response to alkaloid | 0.000152 | 0.002219 | 0.000736 | 2 |
| BP | GO:0071456 | cellular response to hypoxia | 0.000152 | 0.002219 | 0.000736 | 3 |
| BP | GO:0071229 | cellular response to acid chemical | 0.000157 | 0.002219 | 0.000736 | 3 |
| BP | GO:0009612 | response to mechanical stimulus | 0.000159 | 0.002219 | 0.000736 | 3 |
| BP | GO:0043523 | regulation of neuron apoptotic process | 0.000159 | 0.002219 | 0.000736 | 3 |
| BP | GO:0032205 | negative regulation of telomere maintenance | 0.000161 | 0.002219 | 0.000736 | 2 |
| BP | GO:0034390 | smooth muscle cell apoptotic process | 0.000161 | 0.002219 | 0.000736 | 2 |
| BP | GO:0034391 | regulation of smooth muscle cell apoptotic process | 0.000161 | 0.002219 | 0.000736 | 2 |
| BP | GO:0071219 | cellular response to molecule of bacterial origin | 0.000163 | 0.002224 | 0.000738 | 3 |
| BP | GO:0071241 | cellular response to inorganic substance | 0.00017 | 0.002256 | 0.000748 | 3 |
| BP | GO:0032869 | cellular response to insulin stimulus | 0.000173 | 0.002256 | 0.000748 | 3 |
| BP | GO:0016485 | protein processing | 0.000175 | 0.002256 | 0.000748 | 3 |
| BP | GO:0036294 | cellular response to decreased oxygen levels | 0.000175 | 0.002256 | 0.000748 | 3 |
| BP | GO:0043393 | regulation of protein binding | 0.000175 | 0.002256 | 0.000748 | 3 |
| BP | GO:0016572 | histone phosphorylation | 0.00018 | 0.002268 | 0.000753 | 2 |
| BP | GO:2000279 | negative regulation of DNA biosynthetic process | 0.00018 | 0.002268 | 0.000753 | 2 |
| BP | GO:0033146 | regulation of intracellular estrogen receptor signaling pathway | 0.000189 | 0.002341 | 0.000777 | 2 |
| BP | GO:0038179 | neurotrophin signaling pathway | 0.000189 | 0.002341 | 0.000777 | 2 |
| BP | GO:0097191 | extrinsic apoptotic signaling pathway | 0.000192 | 0.002352 | 0.00078 | 3 |
| BP | GO:0045124 | regulation of bone resorption | 0.000199 | 0.002415 | 0.000801 | 2 |
| CC | GO:0000307 | cyclin-dependent protein kinase holoenzyme complex | 0.000197 | 0.00791 | 0.003304 | 2 |
| CC | GO:0061695 | transferase complex, transferring phosphorus-containing groups | 0.000251 | 0.00791 | 0.003304 | 3 |
| MF | GO:0035258 | steroid hormone receptor binding | 1.40E-07 | 1.61E-05 | 4.43E-06 | 4 |
| MF | GO:0016922 | nuclear receptor binding | 2.88E-07 | 1.66E-05 | 4.55E-06 | 4 |
| MF | GO:0035257 | nuclear hormone receptor binding | 1.06E-06 | 4.05E-05 | 1.11E-05 | 4 |
| MF | GO:0030331 | estrogen receptor binding | 1.47E-06 | 4.24E-05 | 1.16E-05 | 3 |
| MF | GO:0051427 | hormone receptor binding | 2.31E-06 | 5.32E-05 | 1.46E-05 | 4 |
| MF | GO:0061629 | RNA polymerase II-specific DNA-binding transcription factor binding | 1.15E-05 | 0.00022 | 6.03E-05 | 4 |
| MF | GO:0140297 | DNA-binding transcription factor binding | 2.91E-05 | 0.000478 | 0.000131 | 4 |
| MF | GO:0008022 | protein C-terminus binding | 0.000132 | 0.001897 | 0.000521 | 3 |
| MF | GO:0016538 | cyclin-dependent protein serine/threonine kinase regulator activity | 0.000333 | 0.004258 | 0.001169 | 2 |

Note: The first hundred results of BP were showed.
