# Supplementary material for: Identification of key pharmacological components and targets for Aidi injection in the treatment of pancreatic cancer by UPLC-MS, network pharmacology, and in vivo experiments
Source: Chin Med. 2023 Jan 14;18:7. doi: 10.1186/s13020-023-00710-2 (PMC9840244; doi:10.1186/s13020-023-00710-2)
Supplement: Supplementary file 5 — Additional file 5: Table S5. The information of KEGG enrichment analysis of ADI-pancreatic cancer PPI network. [file 13020_2023_710_MOESM5_ESM.docx]

# Table S4 The information of KEGG enrichment analysis of ADI-pancreatic cancer PPI network

| **ID** | **Description** | **pvalue** | **p.adjust** | **qvalue** | **Count** |
| --- | --- | --- | --- | --- | --- |
| hsa05161 | Hepatitis B | 3.12E-20 | 6.56E-18 | 1.84E-18 | 20 |
| hsa05205 | Proteoglycans in cancer | 1.58E-19 | 1.65E-17 | 4.64E-18 | 21 |
| hsa05417 | Lipid and atherosclerosis | 4.31E-19 | 3.02E-17 | 8.47E-18 | 21 |
| hsa01522 | Endocrine resistance | 3.17E-18 | 1.55E-16 | 4.34E-17 | 16 |
| hsa01521 | EGFR tyrosine kinase inhibitor resistance | 3.68E-18 | 1.55E-16 | 4.34E-17 | 15 |
| hsa05163 | Human cytomegalovirus infection | 2.38E-17 | 8.32E-16 | 2.33E-16 | 20 |
| hsa05167 | Kaposi sarcoma-associated herpesvirus infection | 2.83E-17 | 8.48E-16 | 2.38E-16 | 19 |
| hsa04151 | PI3K-Akt signaling pathway | 8.80E-16 | 2.31E-14 | 6.49E-15 | 22 |
| hsa04210 | Apoptosis | 1.77E-14 | 4.13E-13 | 1.16E-13 | 15 |
| hsa05162 | Measles | 2.46E-14 | 5.17E-13 | 1.45E-13 | 15 |
| hsa05169 | Epstein-Barr virus infection | 3.98E-13 | 7.61E-12 | 2.14E-12 | 16 |
| hsa05418 | Fluid shear stress and atherosclerosis | 5.34E-13 | 9.11E-12 | 2.56E-12 | 14 |
| hsa05210 | Colorectal cancer | 5.64E-13 | 9.11E-12 | 2.56E-12 | 12 |
| hsa04010 | MAPK signaling pathway | 8.77E-13 | 1.32E-11 | 3.69E-12 | 18 |
| hsa05222 | Small cell lung cancer | 1.30E-12 | 1.82E-11 | 5.10E-12 | 12 |
| hsa04657 | IL-17 signaling pathway | 1.69E-12 | 2.22E-11 | 6.22E-12 | 12 |
| hsa05218 | Melanoma | 2.04E-12 | 2.39E-11 | 6.70E-12 | 11 |
| hsa05223 | Non-small cell lung cancer | 2.04E-12 | 2.39E-11 | 6.70E-12 | 11 |
| hsa05215 | Prostate cancer | 2.48E-12 | 2.74E-11 | 7.70E-12 | 12 |
| hsa05160 | Hepatitis C | 2.90E-12 | 3.04E-11 | 8.54E-12 | 14 |
| hsa04933 | AGE-RAGE signaling pathway in diabetic complications | 3.60E-12 | 3.60E-11 | 1.01E-11 | 12 |
| hsa05212 | Pancreatic cancer | 3.79E-12 | 3.62E-11 | 1.02E-11 | 11 |
| hsa05165 | Human papillomavirus infection | 6.58E-12 | 6.00E-11 | 1.69E-11 | 18 |
| hsa04370 | VEGF signaling pathway | 7.61E-12 | 6.44E-11 | 1.81E-11 | 10 |
| hsa05219 | Bladder cancer | 7.66E-12 | 6.44E-11 | 1.81E-11 | 9 |
| hsa05170 | Human immunodeficiency virus 1 infection | 1.26E-11 | 1.02E-10 | 2.86E-11 | 15 |
| hsa05224 | Breast cancer | 2.15E-11 | 1.67E-10 | 4.69E-11 | 13 |
| hsa05166 | Human T-cell leukemia virus 1 infection | 2.45E-11 | 1.83E-10 | 5.15E-11 | 15 |
| hsa04215 | Apoptosis - multiple species | 3.88E-11 | 2.81E-10 | 7.89E-11 | 8 |
| hsa04115 | p53 signaling pathway | 6.97E-11 | 4.88E-10 | 1.37E-10 | 10 |
| hsa04510 | Focal adhesion | 8.39E-11 | 5.68E-10 | 1.59E-10 | 14 |
| hsa05225 | Hepatocellular carcinoma | 1.17E-10 | 7.69E-10 | 2.16E-10 | 13 |
| hsa04625 | C-type lectin receptor signaling pathway | 1.27E-10 | 8.09E-10 | 2.27E-10 | 11 |
| hsa04668 | TNF signaling pathway | 2.86E-10 | 1.72E-09 | 4.82E-10 | 11 |
| hsa05145 | Toxoplasmosis | 2.86E-10 | 1.72E-09 | 4.82E-10 | 11 |
| hsa04012 | ErbB signaling pathway | 3.29E-10 | 1.92E-09 | 5.39E-10 | 10 |
| hsa05226 | Gastric cancer | 4.19E-10 | 2.38E-09 | 6.67E-10 | 12 |
| hsa04932 | Non-alcoholic fatty liver disease | 6.63E-10 | 3.67E-09 | 1.03E-09 | 12 |
| hsa05131 | Shigellosis | 1.29E-09 | 6.95E-09 | 1.95E-09 | 14 |
| hsa04926 | Relaxin signaling pathway | 1.32E-09 | 6.95E-09 | 1.95E-09 | 11 |
| hsa01524 | Platinum drug resistance | 1.78E-09 | 9.13E-09 | 2.56E-09 | 9 |
| hsa05207 | Chemical carcinogenesis - receptor activation | 2.11E-09 | 1.06E-08 | 2.97E-09 | 13 |
| hsa05214 | Glioma | 2.28E-09 | 1.11E-08 | 3.13E-09 | 9 |
| hsa04660 | T cell receptor signaling pathway | 2.47E-09 | 1.18E-08 | 3.32E-09 | 10 |
| hsa05152 | Tuberculosis | 3.72E-09 | 1.73E-08 | 4.87E-09 | 12 |
| hsa05213 | Endometrial cancer | 6.13E-09 | 2.80E-08 | 7.86E-09 | 8 |
| hsa04919 | Thyroid hormone signaling pathway | 1.09E-08 | 4.89E-08 | 1.37E-08 | 10 |
| hsa05203 | Viral carcinogenesis | 1.53E-08 | 6.71E-08 | 1.88E-08 | 12 |
| hsa04912 | GnRH signaling pathway | 1.58E-08 | 6.78E-08 | 1.90E-08 | 9 |
| hsa04015 | Rap1 signaling pathway | 2.13E-08 | 8.93E-08 | 2.51E-08 | 12 |
| hsa04664 | Fc epsilon RI signaling pathway | 2.24E-08 | 9.21E-08 | 2.59E-08 | 8 |
| hsa04068 | FoxO signaling pathway | 2.36E-08 | 9.53E-08 | 2.68E-08 | 10 |
| hsa05206 | MicroRNAs in cancer | 2.42E-08 | 9.57E-08 | 2.69E-08 | 14 |
| hsa05164 | Influenza A | 2.78E-08 | 1.04E-07 | 2.92E-08 | 11 |
| hsa04917 | Prolactin signaling pathway | 2.83E-08 | 1.04E-07 | 2.92E-08 | 8 |
| hsa05120 | Epithelial cell signaling in Helicobacter pylori infection | 2.83E-08 | 1.04E-07 | 2.92E-08 | 8 |
| hsa05230 | Central carbon metabolism in cancer | 2.83E-08 | 1.04E-07 | 2.92E-08 | 8 |
| hsa05135 | Yersinia infection | 3.63E-08 | 1.32E-07 | 3.69E-08 | 10 |
| hsa04915 | Estrogen signaling pathway | 3.89E-08 | 1.39E-07 | 3.89E-08 | 10 |
| hsa05208 | Chemical carcinogenesis - reactive oxygen species | 4.15E-08 | 1.45E-07 | 4.08E-08 | 12 |
| hsa04659 | Th17 cell differentiation | 5.92E-08 | 2.04E-07 | 5.72E-08 | 9 |
| hsa04014 | Ras signaling pathway | 6.44E-08 | 2.18E-07 | 6.12E-08 | 12 |
| hsa05130 | Pathogenic Escherichia coli infection | 1.13E-07 | 3.76E-07 | 1.05E-07 | 11 |
| hsa04071 | Sphingolipid signaling pathway | 1.38E-07 | 4.52E-07 | 1.27E-07 | 9 |
| hsa05132 | Salmonella infection | 1.40E-07 | 4.53E-07 | 1.27E-07 | 12 |
| hsa05235 | PD-L1 expression and PD-1 checkpoint pathway in cancer | 1.90E-07 | 6.06E-07 | 1.70E-07 | 8 |
| hsa04914 | Progesterone-mediated oocyte maturation | 5.50E-07 | 1.70E-06 | 4.77E-07 | 8 |
| hsa05142 | Chagas disease | 5.50E-07 | 1.70E-06 | 4.77E-07 | 8 |
| hsa04140 | Autophagy - animal | 5.91E-07 | 1.80E-06 | 5.05E-07 | 9 |
| hsa04620 | Toll-like receptor signaling pathway | 6.39E-07 | 1.92E-06 | 5.38E-07 | 8 |
| hsa05202 | Transcriptional misregulation in cancer | 8.67E-07 | 2.56E-06 | 7.20E-07 | 10 |
| hsa05220 | Chronic myeloid leukemia | 9.97E-07 | 2.91E-06 | 8.16E-07 | 7 |
| hsa04218 | Cellular senescence | 1.39E-06 | 3.99E-06 | 1.12E-06 | 9 |
| hsa04217 | Necroptosis | 1.63E-06 | 4.61E-06 | 1.29E-06 | 9 |
| hsa04722 | Neurotrophin signaling pathway | 1.79E-06 | 4.95E-06 | 1.39E-06 | 8 |
| hsa04935 | Growth hormone synthesis, secretion and action | 1.79E-06 | 4.95E-06 | 1.39E-06 | 8 |
| hsa05134 | Legionellosis | 2.84E-06 | 7.76E-06 | 2.18E-06 | 6 |
| hsa05022 | Pathways of neurodegeneration - multiple diseases | 4.53E-06 | 1.22E-05 | 3.42E-06 | 14 |
| hsa05171 | Coronavirus disease - COVID-19 | 4.82E-06 | 1.28E-05 | 3.59E-06 | 10 |
| hsa05231 | Choline metabolism in cancer | 5.58E-06 | 1.47E-05 | 4.11E-06 | 7 |
| hsa05321 | Inflammatory bowel disease | 6.20E-06 | 1.61E-05 | 4.51E-06 | 6 |
| hsa05211 | Renal cell carcinoma | 8.80E-06 | 2.25E-05 | 6.32E-06 | 6 |
| hsa04137 | Mitophagy - animal | 1.13E-05 | 2.83E-05 | 7.94E-06 | 6 |
| hsa04066 | HIF-1 signaling pathway | 1.13E-05 | 2.83E-05 | 7.94E-06 | 7 |
| hsa05415 | Diabetic cardiomyopathy | 1.21E-05 | 2.99E-05 | 8.38E-06 | 9 |
| hsa04150 | mTOR signaling pathway | 1.29E-05 | 3.14E-05 | 8.81E-06 | 8 |
| hsa05133 | Pertussis | 1.54E-05 | 3.72E-05 | 1.05E-05 | 6 |
| hsa05140 | Leishmaniasis | 1.66E-05 | 3.97E-05 | 1.11E-05 | 6 |
| hsa04630 | JAK-STAT signaling pathway | 1.78E-05 | 4.19E-05 | 1.18E-05 | 8 |
| hsa04110 | Cell cycle | 2.92E-05 | 6.81E-05 | 1.91E-05 | 7 |
| hsa04380 | Osteoclast differentiation | 3.23E-05 | 7.46E-05 | 2.09E-05 | 7 |
| hsa04211 | Longevity regulating pathway | 3.82E-05 | 8.71E-05 | 2.45E-05 | 6 |
| hsa04621 | NOD-like receptor signaling pathway | 4.45E-05 | 0.000100406 | 2.82E-05 | 8 |
| hsa01523 | Antifolate resistance | 6.47E-05 | 0.000144533 | 4.06E-05 | 4 |
| hsa04550 | Signaling pathways regulating pluripotency of stem cells | 6.57E-05 | 0.000145314 | 4.08E-05 | 7 |
| hsa05010 | Alzheimer disease | 7.22E-05 | 0.00015786 | 4.43E-05 | 11 |
| hsa04931 | Insulin resistance | 0.000113201 | 0.000245074 | 6.88E-05 | 6 |
| hsa05020 | Prion disease | 0.000123708 | 0.000265088 | 7.44E-05 | 9 |
| hsa04920 | Adipocytokine signaling pathway | 0.000125608 | 0.000266441 | 7.48E-05 | 5 |
| hsa05216 | Thyroid cancer | 0.000131518 | 0.000276188 | 7.75E-05 | 4 |
| hsa04020 | Calcium signaling pathway | 0.000283142 | 0.000588711 | 0.000165252 | 8 |
| hsa05016 | Huntington disease | 0.000291928 | 0.000601027 | 0.000168709 | 9 |
| hsa04613 | Neutrophil extracellular trap formation | 0.000384386 | 0.000783699 | 0.000219986 | 7 |
| hsa04658 | Th1 and Th2 cell differentiation | 0.000484868 | 0.00097906 | 0.000274824 | 5 |
| hsa04750 | Inflammatory mediator regulation of TRP channels | 0.000648053 | 0.001296106 | 0.000363819 | 5 |
| hsa05168 | Herpes simplex virus 1 infection | 0.00068366 | 0.001354421 | 0.000380188 | 11 |
| hsa04921 | Oxytocin signaling pathway | 0.000770594 | 0.001512211 | 0.00042448 | 6 |
| hsa05146 | Amoebiasis | 0.000777708 | 0.001512211 | 0.00042448 | 5 |
| hsa04064 | NF-kappa B signaling pathway | 0.000849375 | 0.00163641 | 0.000459343 | 5 |
| hsa05416 | Viral myocarditis | 0.000859124 | 0.001640145 | 0.000460392 | 4 |
| hsa05217 | Basal cell carcinoma | 0.001032853 | 0.001954045 | 0.000548504 | 4 |
| hsa04310 | Wnt signaling pathway | 0.001139644 | 0.002136832 | 0.000599813 | 6 |
| hsa04670 | Leukocyte transendothelial migration | 0.001284106 | 0.002386391 | 0.000669864 | 5 |
| hsa05221 | Acute myeloid leukemia | 0.001301095 | 0.002396754 | 0.000672773 | 4 |
| hsa04726 | Serotonergic synapse | 0.001335195 | 0.002438182 | 0.000684402 | 5 |
| hsa04622 | RIG-I-like receptor signaling pathway | 0.001531828 | 0.002773138 | 0.000778425 | 4 |
| hsa04152 | AMPK signaling pathway | 0.001613514 | 0.002873597 | 0.000806624 | 5 |
| hsa04520 | Adherens junction | 0.001614688 | 0.002873597 | 0.000806624 | 4 |
| hsa04360 | Axon guidance | 0.001827311 | 0.003224666 | 0.000905169 | 6 |
| hsa05143 | African trypanosomiasis | 0.002283347 | 0.003995858 | 0.001121644 | 3 |
| hsa04062 | Chemokine signaling pathway | 0.002394376 | 0.004155528 | 0.001166464 | 6 |
| hsa04728 | Dopaminergic synapse | 0.002453566 | 0.00421115 | 0.001182077 | 5 |
| hsa05330 | Allograft rejection | 0.002466531 | 0.00421115 | 0.001182077 | 3 |
| hsa04662 | B cell receptor signaling pathway | 0.002741925 | 0.004613104 | 0.001294907 | 4 |
| hsa05012 | Parkinson disease | 0.002745896 | 0.004613104 | 0.001294907 | 7 |
| hsa05332 | Graft-versus-host disease | 0.003289225 | 0.005482041 | 0.001538819 | 3 |
| hsa04940 | Type I diabetes mellitus | 0.003518066 | 0.005809879 | 0.001630843 | 3 |
| hsa04540 | Gap junction | 0.003541259 | 0.005809879 | 0.001630843 | 4 |
| hsa05014 | Amyotrophic lateral sclerosis | 0.004212919 | 0.00685824 | 0.00192512 | 8 |
| hsa05323 | Rheumatoid arthritis | 0.004318191 | 0.00697554 | 0.001958046 | 4 |
| hsa04810 | Regulation of actin cytoskeleton | 0.004489018 | 0.007196136 | 0.002019968 | 6 |
| hsa05144 | Malaria | 0.005393112 | 0.008579951 | 0.002408407 | 3 |
| hsa04913 | Ovarian steroidogenesis | 0.005701315 | 0.009002076 | 0.002526899 | 3 |
| hsa04928 | Parathyroid hormone synthesis, secretion and action | 0.006859271 | 0.010749604 | 0.003017433 | 4 |
| hsa04730 | Long-term depression | 0.008952757 | 0.013926511 | 0.003909196 | 3 |
| hsa04213 | Longevity regulating pathway - multiple species | 0.009795447 | 0.015125323 | 0.004245705 | 3 |
| hsa04929 | GnRH secretion | 0.010682972 | 0.016375359 | 0.004596592 | 3 |
| hsa05031 | Amphetamine addiction | 0.013100699 | 0.019935846 | 0.005596027 | 3 |
| hsa04650 | Natural killer cell mediated cytotoxicity | 0.014181737 | 0.021425646 | 0.006014216 | 4 |
| hsa04910 | Insulin signaling pathway | 0.016471074 | 0.024706611 | 0.006935189 | 4 |
| hsa05100 | Bacterial invasion of epithelial cells | 0.017570752 | 0.026169205 | 0.007345742 | 3 |
| hsa05017 | Spinocerebellar ataxia | 0.018980977 | 0.028070459 | 0.007879427 | 4 |
| hsa04024 | cAMP signaling pathway | 0.019868086 | 0.029176909 | 0.00819001 | 5 |
| hsa05310 | Asthma | 0.02054689 | 0.029964215 | 0.008411008 | 2 |
| hsa04072 | Phospholipase D signaling pathway | 0.021245195 | 0.030558158 | 0.008577728 | 4 |
| hsa04723 | Retrograde endocannabinoid signaling | 0.021245195 | 0.030558158 | 0.008577728 | 4 |
| hsa03410 | Base excision repair | 0.023119003 | 0.033027147 | 0.009270778 | 2 |
| hsa04934 | Cushing syndrome | 0.024684527 | 0.035025343 | 0.009831675 | 4 |
| hsa04666 | Fc gamma R-mediated phagocytosis | 0.032046233 | 0.045165831 | 0.012678128 | 3 |
| hsa04530 | Tight junction | 0.03252915 | 0.04554081 | 0.012783385 | 4 |
| hsa04640 | Hematopoietic cell lineage | 0.033753299 | 0.046941674 | 0.01317661 | 3 |
| hsa04916 | Melanogenesis | 0.035507102 | 0.049055865 | 0.013770067 | 3 |
| hsa04930 | Type II diabetes mellitus | 0.042721786 | 0.058637746 | 0.016459718 | 2 |
| hsa04672 | Intestinal immune network for IgA production | 0.047888104 | 0.06530196 | 0.018330375 | 2 |
| hsa04961 | Endocrine and other factor-regulated calcium reabsorption | 0.055110278 | 0.074186913 | 0.020824397 | 2 |
| hsa05320 | Autoimmune thyroid disease | 0.055110278 | 0.074186913 | 0.020824397 | 2 |
| hsa04611 | Platelet activation | 0.058952911 | 0.078854212 | 0.022134516 | 3 |
| hsa04340 | Hedgehog signaling pathway | 0.06076276 | 0.080760631 | 0.022669651 | 2 |
| hsa04923 | Regulation of lipolysis in adipocytes | 0.062689707 | 0.082797727 | 0.023241467 | 2 |
| hsa04114 | Oocyte meiosis | 0.067238382 | 0.088250377 | 0.024772036 | 3 |
| hsa00590 | Arachidonic acid metabolism | 0.070601091 | 0.09208838 | 0.02584937 | 2 |
| hsa04720 | Long-term potentiation | 0.083037946 | 0.107641782 | 0.030215237 | 2 |
| hsa04261 | Adrenergic signaling in cardiomyocytes | 0.092213822 | 0.118803084 | 0.033348234 | 3 |
| hsa04390 | Hippo signaling pathway | 0.102267179 | 0.130951876 | 0.036758421 | 3 |
| hsa04612 | Antigen processing and presentation | 0.107385241 | 0.136672125 | 0.038364105 | 2 |
| hsa00910 | Nitrogen metabolism | 0.115200153 | 0.145735133 | 0.040908108 | 1 |
| hsa04141 | Protein processing in endoplasmic reticulum | 0.123613839 | 0.155442553 | 0.043632997 | 3 |
| hsa04727 | GABAergic synapse | 0.133372286 | 0.166669414 | 0.046784397 | 2 |
| hsa00670 | One carbon pool by folate | 0.134129195 | 0.166669414 | 0.046784397 | 1 |
| hsa04070 | Phosphatidylinositol signaling system | 0.153082769 | 0.189102245 | 0.053081332 | 2 |
| hsa04060 | Cytokine-cytokine receptor interaction | 0.160228647 | 0.196104126 | 0.055046772 | 4 |
| hsa04061 | Viral protein interaction with cytokine and cytokine receptor | 0.160618618 | 0.196104126 | 0.055046772 | 2 |
| hsa04972 | Pancreatic secretion | 0.165681016 | 0.201115685 | 0.056453525 | 2 |
| hsa04725 | Cholinergic synapse | 0.193988658 | 0.234124243 | 0.065719086 | 2 |
| hsa04960 | Aldosterone-regulated sodium reabsorption | 0.234110982 | 0.280933179 | 0.078858436 | 1 |
| hsa04270 | Vascular smooth muscle contraction | 0.246781519 | 0.294455221 | 0.082654097 | 2 |
| hsa04216 | Ferroptosis | 0.255934335 | 0.303650905 | 0.085235342 | 1 |
| hsa04371 | Apelin signaling pathway | 0.260112414 | 0.306874196 | 0.086140125 | 2 |
| hsa04144 | Endocytosis | 0.269912385 | 0.316656988 | 0.088886172 | 3 |
| hsa02010 | ABC transporters | 0.277146269 | 0.323337314 | 0.090761351 | 1 |
| hsa04973 | Carbohydrate digestion and absorption | 0.287528235 | 0.333596294 | 0.093641065 | 1 |
| hsa05030 | Cocaine addiction | 0.297763624 | 0.343573412 | 0.09644166 | 1 |
| hsa00270 | Cysteine and methionine metabolism | 0.302826989 | 0.345617759 | 0.097015511 | 1 |
| hsa05110 | Vibrio cholerae infection | 0.302826989 | 0.345617759 | 0.097015511 | 1 |
| hsa00240 | Pyrimidine metabolism | 0.332462311 | 0.37738965 | 0.105933937 | 1 |
| hsa04022 | cGMP-PKG signaling pathway | 0.337194823 | 0.380703832 | 0.106864234 | 2 |
| hsa04330 | Notch signaling pathway | 0.346812143 | 0.389468182 | 0.109324402 | 1 |
| hsa04623 | Cytosolic DNA-sensing pathway | 0.365474595 | 0.408242899 | 0.114594498 | 1 |
| hsa05034 | Alcoholism | 0.389135991 | 0.432373324 | 0.12136795 | 2 |
| hsa05204 | Chemical carcinogenesis - DNA adducts | 0.392489794 | 0.433804509 | 0.121769687 | 1 |
| hsa00562 | Inositol phosphate metabolism | 0.409868575 | 0.450640842 | 0.126495675 | 1 |
| hsa04918 | Thyroid hormone synthesis | 0.418373806 | 0.457433182 | 0.128402297 | 1 |
| hsa03320 | PPAR signaling pathway | 0.42258113 | 0.457433182 | 0.128402297 | 1 |
| hsa04971 | Gastric acid secretion | 0.42258113 | 0.457433182 | 0.128402297 | 1 |
| hsa04911 | Insulin secretion | 0.463043128 | 0.49866183 | 0.13997525 | 1 |
| hsa04976 | Bile secretion | 0.474628847 | 0.508530907 | 0.142745518 | 1 |
| hsa05410 | Hypertrophic cardiomyopathy | 0.478435884 | 0.510007795 | 0.143160083 | 1 |
| hsa05032 | Morphine addiction | 0.482215807 | 0.511441007 | 0.143562388 | 1 |
| hsa04970 | Salivary secretion | 0.489695064 | 0.516763635 | 0.145056459 | 1 |
| hsa04350 | TGF-beta signaling pathway | 0.493394775 | 0.518064514 | 0.145421618 | 1 |
| hsa04714 | Thermogenesis | 0.49884247 | 0.520545598 | 0.146118063 | 2 |
| hsa05414 | Dilated cardiomyopathy | 0.50071529 | 0.520545598 | 0.146118063 | 1 |
| hsa04713 | Circadian entrainment | 0.504336462 | 0.521727374 | 0.146449789 | 1 |
| hsa04925 | Aldosterone synthesis and secretion | 0.50793182 | 0.522870991 | 0.146770805 | 1 |
| hsa04974 | Protein digestion and absorption | 0.525527691 | 0.538345439 | 0.151114509 | 1 |
| hsa04922 | Glucagon signaling pathway | 0.539157853 | 0.549626938 | 0.154281246 | 1 |
| hsa04724 | Glutamatergic synapse | 0.562090687 | 0.570236929 | 0.160066506 | 1 |
| hsa00190 | Oxidative phosphorylation | 0.621610308 | 0.62758733 | 0.176164865 | 1 |
| hsa05322 | Systemic lupus erythematosus | 0.627105472 | 0.630105977 | 0.176871853 | 1 |
| hsa04080 | Neuroactive ligand-receptor interaction | 0.918393647 | 0.918393647 | 0.257794708 | 1 |

**The information of KEGG enrichment analysis for module 1.**

| **ID** | **Description** | **pvalue** | **p.adjust** | **qvalue** | **Count** |
| --- | --- | --- | --- | --- | --- |
| hsa05205 | Proteoglycans in cancer | 2.80E-16 | 4.88E-14 | 1.65E-14 | 12 |
| hsa01522 | Endocrine resistance | 5.42E-10 | 4.71E-08 | 1.60E-08 | 7 |
| hsa05167 | Kaposi sarcoma-associated herpesvirus infection | 1.91E-09 | 1.11E-07 | 3.75E-08 | 8 |
| hsa05417 | Lipid and atherosclerosis | 4.31E-09 | 1.60E-07 | 5.42E-08 | 8 |
| hsa05218 | Melanoma | 4.60E-09 | 1.60E-07 | 5.42E-08 | 6 |
| hsa05418 | Fluid shear stress and atherosclerosis | 6.39E-09 | 1.85E-07 | 6.27E-08 | 7 |
| hsa01521 | EGFR tyrosine kinase inhibitor resistance | 8.11E-09 | 2.02E-07 | 6.83E-08 | 6 |
| hsa05161 | Hepatitis B | 1.86E-08 | 4.04E-07 | 1.37E-07 | 7 |
| hsa05215 | Prostate cancer | 2.82E-08 | 5.46E-07 | 1.85E-07 | 6 |
| hsa05163 | Human cytomegalovirus infection | 1.79E-07 | 3.12E-06 | 1.06E-06 | 7 |
| hsa04151 | PI3K-Akt signaling pathway | 2.13E-07 | 3.37E-06 | 1.14E-06 | 8 |
| hsa05230 | Central carbon metabolism in cancer | 2.39E-07 | 3.47E-06 | 1.17E-06 | 5 |
| hsa05224 | Breast cancer | 3.40E-07 | 4.56E-06 | 1.54E-06 | 6 |
| hsa05235 | PD-L1 expression and PD-1 checkpoint pathway in cancer | 8.01E-07 | 9.95E-06 | 3.37E-06 | 5 |
| hsa05222 | Small cell lung cancer | 9.45E-07 | 1.10E-05 | 3.72E-06 | 5 |
| hsa04657 | IL-17 signaling pathway | 1.05E-06 | 1.13E-05 | 3.83E-06 | 5 |
| hsa04010 | MAPK signaling pathway | 1.11E-06 | 1.13E-05 | 3.83E-06 | 7 |
| hsa04933 | AGE-RAGE signaling pathway in diabetic complications | 1.43E-06 | 1.39E-05 | 4.69E-06 | 5 |
| hsa04625 | C-type lectin receptor signaling pathway | 1.74E-06 | 1.59E-05 | 5.40E-06 | 5 |
| hsa04659 | Th17 cell differentiation | 2.10E-06 | 1.83E-05 | 6.19E-06 | 5 |
| hsa05169 | Epstein-Barr virus infection | 2.21E-06 | 1.83E-05 | 6.20E-06 | 6 |
| hsa04668 | TNF signaling pathway | 2.52E-06 | 1.90E-05 | 6.45E-06 | 5 |
| hsa05145 | Toxoplasmosis | 2.52E-06 | 1.90E-05 | 6.45E-06 | 5 |
| hsa04071 | Sphingolipid signaling pathway | 3.39E-06 | 2.46E-05 | 8.34E-06 | 5 |
| hsa04068 | FoxO signaling pathway | 5.45E-06 | 3.79E-05 | 1.28E-05 | 5 |
| hsa04915 | Estrogen signaling pathway | 7.04E-06 | 4.70E-05 | 1.59E-05 | 5 |
| hsa05162 | Measles | 7.29E-06 | 4.70E-05 | 1.59E-05 | 5 |
| hsa04550 | Signaling pathways regulating pluripotency of stem cells | 8.38E-06 | 5.20E-05 | 1.76E-05 | 5 |
| hsa04917 | Prolactin signaling pathway | 1.12E-05 | 6.73E-05 | 2.28E-05 | 4 |
| hsa05223 | Non-small cell lung cancer | 1.26E-05 | 7.28E-05 | 2.47E-05 | 4 |
| hsa05160 | Hepatitis C | 1.32E-05 | 7.42E-05 | 2.51E-05 | 5 |
| hsa05214 | Glioma | 1.48E-05 | 8.04E-05 | 2.72E-05 | 4 |
| hsa05225 | Hepatocellular carcinoma | 1.84E-05 | 9.68E-05 | 3.28E-05 | 5 |
| hsa05206 | MicroRNAs in cancer | 2.60E-05 | 0.000133304 | 4.52E-05 | 6 |
| hsa05415 | Diabetic cardiomyopathy | 4.57E-05 | 0.000227419 | 7.70E-05 | 5 |
| hsa04914 | Progesterone-mediated oocyte maturation | 4.99E-05 | 0.000234682 | 7.95E-05 | 4 |
| hsa05142 | Chagas disease | 4.99E-05 | 0.000234682 | 7.95E-05 | 4 |
| hsa04015 | Rap1 signaling pathway | 5.38E-05 | 0.000240287 | 8.14E-05 | 5 |
| hsa04660 | T cell receptor signaling pathway | 5.39E-05 | 0.000240287 | 8.14E-05 | 4 |
| hsa05207 | Chemical carcinogenesis - receptor activation | 5.63E-05 | 0.000244935 | 8.30E-05 | 5 |
| hsa04931 | Insulin resistance | 6.24E-05 | 0.000265018 | 8.98E-05 | 4 |
| hsa04066 | HIF-1 signaling pathway | 6.47E-05 | 0.000268217 | 9.09E-05 | 4 |
| hsa05166 | Human T-cell leukemia virus 1 infection | 7.02E-05 | 0.000283485 | 9.60E-05 | 5 |
| hsa05208 | Chemical carcinogenesis - reactive oxygen species | 7.17E-05 | 0.000283485 | 9.60E-05 | 5 |
| hsa05219 | Bladder cancer | 7.81E-05 | 0.000302098 | 0.000102344 | 3 |
| hsa04919 | Thyroid hormone signaling pathway | 9.73E-05 | 0.000368207 | 0.000124741 | 4 |
| hsa05131 | Shigellosis | 0.000116469 | 0.000431183 | 0.000146075 | 5 |
| hsa05132 | Salmonella infection | 0.000120996 | 0.000438611 | 0.000148592 | 5 |
| hsa04926 | Relaxin signaling pathway | 0.000124849 | 0.00044334 | 0.000150194 | 4 |
| hsa04210 | Apoptosis | 0.000153216 | 0.000533191 | 0.000180633 | 4 |
| hsa05135 | Yersinia infection | 0.00015762 | 0.000537761 | 0.000182182 | 4 |
| hsa04140 | Autophagy - animal | 0.000176159 | 0.000589454 | 0.000199694 | 4 |
| hsa05226 | Gastric cancer | 0.000217912 | 0.000712781 | 0.000241475 | 4 |
| hsa05213 | Endometrial cancer | 0.000221208 | 0.000712781 | 0.000241475 | 3 |
| hsa04370 | VEGF signaling pathway | 0.000232756 | 0.000736354 | 0.000249461 | 3 |
| hsa04150 | mTOR signaling pathway | 0.000253615 | 0.000774192 | 0.000262279 | 4 |
| hsa04932 | Non-alcoholic fatty liver disease | 0.000253615 | 0.000774192 | 0.000262279 | 4 |
| hsa04218 | Cellular senescence | 0.000259952 | 0.000779855 | 0.000264198 | 4 |
| hsa05321 | Inflammatory bowel disease | 0.000310308 | 0.000915145 | 0.000310031 | 3 |
| hsa04664 | Fc epsilon RI signaling pathway | 0.000354645 | 0.00102847 | 0.000348423 | 3 |
| hsa04920 | Adipocytokine signaling pathway | 0.000370283 | 0.001039182 | 0.000352052 | 3 |
| hsa05211 | Renal cell carcinoma | 0.000370283 | 0.001039182 | 0.000352052 | 3 |
| hsa01524 | Platinum drug resistance | 0.000437278 | 0.001188849 | 0.000402756 | 3 |
| hsa04115 | p53 signaling pathway | 0.000437278 | 0.001188849 | 0.000402756 | 3 |
| hsa05152 | Tuberculosis | 0.000448982 | 0.001201891 | 0.000407174 | 4 |
| hsa05165 | Human papillomavirus infection | 0.000457458 | 0.001206025 | 0.000408575 | 5 |
| hsa05212 | Pancreatic cancer | 0.000492331 | 0.00127859 | 0.000433158 | 3 |
| hsa05140 | Leishmaniasis | 0.000511624 | 0.001309157 | 0.000443513 | 3 |
| hsa05202 | Transcriptional misregulation in cancer | 0.000573453 | 0.0014461 | 0.000489907 | 4 |
| hsa04510 | Focal adhesion | 0.000681739 | 0.001694609 | 0.000574096 | 4 |
| hsa05210 | Colorectal cancer | 0.00070746 | 0.001733776 | 0.000587365 | 3 |
| hsa04211 | Longevity regulating pathway | 0.000781994 | 0.00188982 | 0.000640229 | 3 |
| hsa05170 | Human immunodeficiency virus 1 infection | 0.000833016 | 0.001985546 | 0.000672659 | 4 |
| hsa04014 | Ras signaling pathway | 0.001166929 | 0.002707275 | 0.000917165 | 4 |
| hsa05171 | Coronavirus disease - COVID-19 | 0.001166929 | 0.002707275 | 0.000917165 | 4 |
| hsa04620 | Toll-like receptor signaling pathway | 0.001229742 | 0.002815463 | 0.000953817 | 3 |
| hsa04670 | Leukocyte transendothelial migration | 0.001602871 | 0.003622072 | 0.001227078 | 3 |
| hsa04722 | Neurotrophin signaling pathway | 0.00181338 | 0.003994027 | 0.001353088 | 3 |
| hsa04935 | Growth hormone synthesis, secretion and action | 0.00181338 | 0.003994027 | 0.001353088 | 3 |
| hsa04380 | Osteoclast differentiation | 0.002234479 | 0.004859992 | 0.001646458 | 3 |
| hsa05330 | Allograft rejection | 0.002792832 | 0.005999416 | 0.00203247 | 2 |
| hsa05332 | Graft-versus-host disease | 0.003403685 | 0.007222453 | 0.002446808 | 2 |
| hsa04940 | Type I diabetes mellitus | 0.003565318 | 0.00747428 | 0.002532122 | 2 |
| hsa04217 | Necroptosis | 0.004132751 | 0.008560699 | 0.002900176 | 3 |
| hsa04630 | JAK-STAT signaling pathway | 0.00435564 | 0.00891625 | 0.003020629 | 3 |
| hsa05144 | Malaria | 0.004795098 | 0.00970171 | 0.003286726 | 2 |
| hsa04913 | Ovarian steroidogenesis | 0.004984667 | 0.009969334 | 0.003377391 | 2 |
| hsa05164 | Influenza A | 0.005151297 | 0.010185519 | 0.00345063 | 3 |
| hsa05014 | Amyotrophic lateral sclerosis | 0.006068458 | 0.011756203 | 0.003982743 | 4 |
| hsa04923 | Regulation of lipolysis in adipocytes | 0.006193654 | 0.011756203 | 0.003982743 | 2 |
| hsa05134 | Legionellosis | 0.006193654 | 0.011756203 | 0.003982743 | 2 |
| hsa04621 | NOD-like receptor signaling pathway | 0.006215923 | 0.011756203 | 0.003982743 | 3 |
| hsa05010 | Alzheimer disease | 0.007257636 | 0.013500548 | 0.004573688 | 4 |
| hsa04213 | Longevity regulating pathway - multiple species | 0.0072934 | 0.013500548 | 0.004573688 | 2 |
| hsa05130 | Pathogenic Escherichia coli infection | 0.007508953 | 0.01375324 | 0.004659295 | 3 |
| hsa05221 | Acute myeloid leukemia | 0.00847525 | 0.015361391 | 0.005204101 | 2 |
| hsa04622 | RIG-I-like receptor signaling pathway | 0.009223092 | 0.016375695 | 0.005547725 | 2 |
| hsa05120 | Epithelial cell signaling in Helicobacter pylori infection | 0.009223092 | 0.016375695 | 0.005547725 | 2 |
| hsa04520 | Adherens junction | 0.009478751 | 0.016659622 | 0.005643913 | 2 |
| hsa04137 | Mitophagy - animal | 0.009737576 | 0.016943383 | 0.005740045 | 2 |
| hsa05133 | Pertussis | 0.010804297 | 0.01843086 | 0.00624397 | 2 |
| hsa05220 | Chronic myeloid leukemia | 0.010804297 | 0.01843086 | 0.00624397 | 2 |
| hsa04612 | Antigen processing and presentation | 0.011356331 | 0.019184481 | 0.00649928 | 2 |
| hsa05022 | Pathways of neurodegeneration - multiple diseases | 0.01523528 | 0.025489796 | 0.008635381 | 4 |
| hsa04658 | Th1 and Th2 cell differentiation | 0.015559778 | 0.025784774 | 0.008735314 | 2 |
| hsa04912 | GnRH signaling pathway | 0.015882214 | 0.026070804 | 0.008832214 | 2 |
| hsa05231 | Choline metabolism in cancer | 0.017537559 | 0.028394111 | 0.0096193 | 2 |
| hsa05168 | Herpes simplex virus 1 infection | 0.017623931 | 0.028394111 | 0.0096193 | 4 |
| hsa05020 | Prion disease | 0.018179979 | 0.028820119 | 0.009763622 | 3 |
| hsa04061 | Viral protein interaction with cytokine and cytokine receptor | 0.018219616 | 0.028820119 | 0.009763622 | 2 |
| hsa04064 | NF-kappa B signaling pathway | 0.019617353 | 0.030751527 | 0.01041794 | 2 |
| hsa04152 | AMPK signaling pathway | 0.025642925 | 0.039838115 | 0.013496276 | 2 |
| hsa04611 | Platelet activation | 0.027254303 | 0.041966802 | 0.014217428 | 2 |
| hsa04114 | Oocyte meiosis | 0.030171372 | 0.046051042 | 0.015601079 | 2 |
| hsa04728 | Dopaminergic synapse | 0.030598014 | 0.046296126 | 0.015684108 | 2 |
| hsa05017 | Spinocerebellar ataxia | 0.0354502 | 0.0531753 | 0.018014621 | 2 |
| hsa04723 | Retrograde endocannabinoid signaling | 0.037749759 | 0.056140667 | 0.019019222 | 2 |
| hsa04261 | Adrenergic signaling in cardiomyocytes | 0.038685594 | 0.057044859 | 0.019325542 | 2 |
| hsa04613 | Neutrophil extracellular trap formation | 0.059190768 | 0.086547845 | 0.029320504 | 2 |
| hsa04062 | Chemokine signaling pathway | 0.060299438 | 0.087434185 | 0.029620777 | 2 |
| hsa01523 | Antifolate resistance | 0.063220869 | 0.090167469 | 0.030546753 | 1 |
| hsa05310 | Asthma | 0.063220869 | 0.090167469 | 0.030546753 | 1 |
| hsa04215 | Apoptosis - multiple species | 0.065196216 | 0.092228794 | 0.031245084 | 1 |
| hsa05203 | Viral carcinogenesis | 0.067104102 | 0.094162208 | 0.031900083 | 2 |
| hsa05143 | African trypanosomiasis | 0.075014283 | 0.103591153 | 0.035094402 | 1 |
| hsa05216 | Thyroid cancer | 0.075014283 | 0.103591153 | 0.035094402 | 1 |
| hsa04216 | Ferroptosis | 0.082798774 | 0.113440839 | 0.038431258 | 1 |
| hsa04020 | Calcium signaling pathway | 0.088961541 | 0.120932095 | 0.040969131 | 2 |
| hsa04930 | Type II diabetes mellitus | 0.092442689 | 0.124690139 | 0.042242273 | 1 |
| hsa04973 | Carbohydrate digestion and absorption | 0.094359984 | 0.12629721 | 0.042786714 | 1 |
| hsa04672 | Intestinal immune network for IgA production | 0.098183144 | 0.130411199 | 0.044180442 | 1 |
| hsa04961 | Endocrine and other factor-regulated calcium reabsorption | 0.105783938 | 0.137549055 | 0.046598591 | 1 |
| hsa05320 | Autoimmune thyroid disease | 0.105783938 | 0.137549055 | 0.046598591 | 1 |
| hsa05012 | Parkinson disease | 0.105928583 | 0.137549055 | 0.046598591 | 2 |
| hsa04730 | Long-term depression | 0.118940444 | 0.152173804 | 0.051553134 | 1 |
| hsa05416 | Viral myocarditis | 0.118940444 | 0.152173804 | 0.051553134 | 1 |
| hsa00590 | Arachidonic acid metabolism | 0.120805004 | 0.153431173 | 0.051979103 | 1 |
| hsa05217 | Basal cell carcinoma | 0.124522989 | 0.157007247 | 0.053190598 | 1 |
| hsa04060 | Cytokine-cytokine receptor interaction | 0.125835417 | 0.157067845 | 0.053211127 | 2 |
| hsa04929 | GnRH secretion | 0.126376427 | 0.157067845 | 0.053211127 | 1 |
| hsa05016 | Huntington disease | 0.133622623 | 0.164896002 | 0.055863134 | 2 |
| hsa05204 | Chemical carcinogenesis - DNA adducts | 0.135588354 | 0.166143476 | 0.056285751 | 1 |
| hsa00562 | Inositol phosphate metabolism | 0.14289199 | 0.173868576 | 0.058902845 | 1 |
| hsa05100 | Bacterial invasion of epithelial cells | 0.150137517 | 0.181416167 | 0.061459802 | 1 |
| hsa04662 | B cell receptor signaling pathway | 0.15911337 | 0.190936044 | 0.064684927 | 1 |
| hsa04012 | ErbB signaling pathway | 0.164455975 | 0.195995477 | 0.066398952 | 1 |
| hsa05410 | Hypertrophic cardiomyopathy | 0.173289384 | 0.205118046 | 0.069489477 | 1 |
| hsa05323 | Rheumatoid arthritis | 0.178547161 | 0.209913554 | 0.07111409 | 1 |
| hsa04350 | TGF-beta signaling pathway | 0.180292748 | 0.21054321 | 0.071327403 | 1 |
| hsa05414 | Dilated cardiomyopathy | 0.183773455 | 0.212358514 | 0.071942388 | 1 |
| hsa04070 | Phosphatidylinositol signaling system | 0.185508587 | 0.212358514 | 0.071942388 | 1 |
| hsa04666 | Fc gamma R-mediated phagocytosis | 0.185508587 | 0.212358514 | 0.071942388 | 1 |
| hsa04750 | Inflammatory mediator regulation of TRP channels | 0.187240247 | 0.212939889 | 0.072139345 | 1 |
| hsa04640 | Hematopoietic cell lineage | 0.188968441 | 0.213509797 | 0.072332418 | 1 |
| hsa05146 | Amoebiasis | 0.194132297 | 0.21792916 | 0.0738296 | 1 |
| hsa04922 | Glucagon signaling pathway | 0.202670019 | 0.226055021 | 0.076582463 | 1 |
| hsa04725 | Cholinergic synapse | 0.212802911 | 0.235845264 | 0.079899182 | 1 |
| hsa04726 | Serotonergic synapse | 0.21615352 | 0.238042484 | 0.080643552 | 1 |
| hsa04110 | Cell cycle | 0.234343234 | 0.256451086 | 0.086879981 | 1 |
| hsa04650 | Natural killer cell mediated cytotoxicity | 0.242479281 | 0.263696218 | 0.089334472 | 1 |
| hsa00190 | Oxidative phosphorylation | 0.247321778 | 0.267291859 | 0.090552596 | 1 |
| hsa05322 | Systemic lupus erythematosus | 0.250533906 | 0.269091974 | 0.091162435 | 1 |
| hsa04910 | Insulin signaling pathway | 0.252135128 | 0.269150382 | 0.091182223 | 1 |
| hsa04371 | Apelin signaling pathway | 0.25373313 | 0.269204663 | 0.091200612 | 1 |
| hsa04072 | Phospholipase D signaling pathway | 0.269537371 | 0.284239409 | 0.096294053 | 1 |
| hsa04921 | Oxytocin signaling pathway | 0.278868141 | 0.29230757 | 0.099027368 | 1 |
| hsa04310 | Wnt signaling pathway | 0.297194161 | 0.309369283 | 0.104807501 | 1 |
| hsa04022 | cGMP-PKG signaling pathway | 0.298701377 | 0.309369283 | 0.104807501 | 1 |
| hsa04141 | Protein processing in endoplasmic reticulum | 0.304699872 | 0.313714661 | 0.106279619 | 1 |
| hsa04360 | Axon guidance | 0.320947711 | 0.328499422 | 0.11128837 | 1 |
| hsa04810 | Regulation of actin cytoskeleton | 0.371659484 | 0.377353295 | 0.127838987 | 1 |
| hsa04024 | cAMP signaling pathway | 0.373015901 | 0.377353295 | 0.127838987 | 1 |
| hsa04714 | Thermogenesis | 0.390400414 | 0.392657064 | 0.133023567 | 1 |
| hsa04144 | Endocytosis | 0.416262424 | 0.416262424 | 0.141020543 | 1 |

**The information of KEGG enrichment analysis for module 2.**

| **ID** | **Description** | **pvalue** | **p.adjust** | **qvalue** | **Count** |
| --- | --- | --- | --- | --- | --- |
| hsa05162 | Measles | 3.60E-11 | 5.47E-09 | 1.78E-09 | 8 |
| hsa05417 | Lipid and atherosclerosis | 1.20E-09 | 7.99E-08 | 2.60E-08 | 8 |
| hsa05163 | Human cytomegalovirus infection | 1.72E-09 | 7.99E-08 | 2.60E-08 | 8 |
| hsa04151 | PI3K-Akt signaling pathway | 2.10E-09 | 7.99E-08 | 2.60E-08 | 9 |
| hsa05165 | Human papillomavirus infection | 3.61E-08 | 1.10E-06 | 3.57E-07 | 8 |
| hsa04210 | Apoptosis | 8.91E-08 | 2.26E-06 | 7.35E-07 | 6 |
| hsa05131 | Shigellosis | 1.18E-07 | 2.57E-06 | 8.37E-07 | 7 |
| hsa04115 | p53 signaling pathway | 1.46E-07 | 2.76E-06 | 9.00E-07 | 5 |
| hsa05212 | Pancreatic cancer | 1.78E-07 | 3.00E-06 | 9.76E-07 | 5 |
| hsa05160 | Hepatitis C | 2.10E-07 | 3.00E-06 | 9.76E-07 | 6 |
| hsa01521 | EGFR tyrosine kinase inhibitor resistance | 2.17E-07 | 3.00E-06 | 9.76E-07 | 5 |
| hsa04215 | Apoptosis - multiple species | 2.67E-07 | 3.38E-06 | 1.10E-06 | 4 |
| hsa05210 | Colorectal cancer | 3.33E-07 | 3.89E-06 | 1.27E-06 | 5 |
| hsa05222 | Small cell lung cancer | 4.67E-07 | 5.07E-06 | 1.65E-06 | 5 |
| hsa04657 | IL-17 signaling pathway | 5.20E-07 | 5.27E-06 | 1.72E-06 | 5 |
| hsa05167 | Kaposi sarcoma-associated herpesvirus infection | 7.34E-07 | 6.98E-06 | 2.27E-06 | 6 |
| hsa05169 | Epstein-Barr virus infection | 9.31E-07 | 8.33E-06 | 2.71E-06 | 6 |
| hsa05170 | Human immunodeficiency virus 1 infection | 1.24E-06 | 1.04E-05 | 3.40E-06 | 6 |
| hsa05213 | Endometrial cancer | 3.06E-06 | 2.45E-05 | 7.96E-06 | 4 |
| hsa04932 | Non-alcoholic fatty liver disease | 6.21E-06 | 4.72E-05 | 1.54E-05 | 5 |
| hsa05223 | Non-small cell lung cancer | 7.30E-06 | 5.10E-05 | 1.66E-05 | 4 |
| hsa01524 | Platinum drug resistance | 7.72E-06 | 5.10E-05 | 1.66E-05 | 4 |
| hsa05161 | Hepatitis B | 7.72E-06 | 5.10E-05 | 1.66E-05 | 5 |
| hsa04012 | ErbB signaling pathway | 1.42E-05 | 8.97E-05 | 2.92E-05 | 4 |
| hsa05130 | Pathogenic Escherichia coli infection | 2.00E-05 | 0.000121612 | 3.96E-05 | 5 |
| hsa04510 | Focal adhesion | 2.20E-05 | 0.000128891 | 4.20E-05 | 5 |
| hsa05203 | Viral carcinogenesis | 2.37E-05 | 0.000129873 | 4.23E-05 | 5 |
| hsa05215 | Prostate cancer | 2.39E-05 | 0.000129873 | 4.23E-05 | 4 |
| hsa01522 | Endocrine resistance | 2.49E-05 | 0.000130587 | 4.25E-05 | 4 |
| hsa04933 | AGE-RAGE signaling pathway in diabetic complications | 2.70E-05 | 0.000136731 | 4.45E-05 | 4 |
| hsa05135 | Yersinia infection | 9.28E-05 | 0.000455079 | 0.000148121 | 4 |
| hsa05418 | Fluid shear stress and atherosclerosis | 9.82E-05 | 0.000466446 | 0.000151821 | 4 |
| hsa05224 | Breast cancer | 0.000122052 | 0.000558578 | 0.000181809 | 4 |
| hsa05022 | Pathways of neurodegeneration - multiple diseases | 0.00012722 | 0.000558578 | 0.000181809 | 6 |
| hsa05226 | Gastric cancer | 0.00012862 | 0.000558578 | 0.000181809 | 4 |
| hsa05134 | Legionellosis | 0.000141954 | 0.000599362 | 0.000195083 | 3 |
| hsa04370 | VEGF signaling pathway | 0.000157366 | 0.000646475 | 0.000210418 | 3 |
| hsa04630 | JAK-STAT signaling pathway | 0.000177767 | 0.000711066 | 0.000231441 | 4 |
| hsa05225 | Hepatocellular carcinoma | 0.000204526 | 0.000797125 | 0.000259452 | 4 |
| hsa05321 | Inflammatory bowel disease | 0.000210031 | 0.000798119 | 0.000259776 | 3 |
| hsa05164 | Influenza A | 0.000223914 | 0.000830121 | 0.000270192 | 4 |
| hsa05152 | Tuberculosis | 0.000266638 | 0.000964975 | 0.000314085 | 4 |
| hsa04621 | NOD-like receptor signaling pathway | 0.000290076 | 0.001025385 | 0.000333747 | 4 |
| hsa05220 | Chronic myeloid leukemia | 0.000333911 | 0.00114681 | 0.000373269 | 3 |
| hsa05202 | Transcriptional misregulation in cancer | 0.000341367 | 0.00114681 | 0.000373269 | 4 |
| hsa05140 | Leishmaniasis | 0.000347061 | 0.00114681 | 0.000373269 | 3 |
| hsa05166 | Human T-cell leukemia virus 1 infection | 0.000592845 | 0.001876076 | 0.000610634 | 4 |
| hsa05208 | Chemical carcinogenesis - reactive oxygen species | 0.000603008 | 0.001876076 | 0.000610634 | 4 |
| hsa05323 | Rheumatoid arthritis | 0.000604788 | 0.001876076 | 0.000610634 | 3 |
| hsa05142 | Chagas disease | 0.000792232 | 0.002404363 | 0.000782584 | 3 |
| hsa04620 | Toll-like receptor signaling pathway | 0.000838363 | 0.002404363 | 0.000782584 | 3 |
| hsa04625 | C-type lectin receptor signaling pathway | 0.000838363 | 0.002404363 | 0.000782584 | 3 |
| hsa04660 | T cell receptor signaling pathway | 0.000838363 | 0.002404363 | 0.000782584 | 3 |
| hsa05132 | Salmonella infection | 0.000913447 | 0.002571183 | 0.000836881 | 4 |
| hsa04659 | Th17 cell differentiation | 0.0009357 | 0.002585936 | 0.000841683 | 3 |
| hsa04668 | TNF signaling pathway | 0.001039965 | 0.00277324 | 0.000902647 | 3 |
| hsa05145 | Toxoplasmosis | 0.001039965 | 0.00277324 | 0.000902647 | 3 |
| hsa04722 | Neurotrophin signaling pathway | 0.001239674 | 0.0032488 | 0.001057435 | 3 |
| hsa05020 | Prion disease | 0.001288122 | 0.003295559 | 0.001072654 | 4 |
| hsa04919 | Thyroid hormone signaling pathway | 0.001300879 | 0.003295559 | 0.001072654 | 3 |
| hsa04110 | Cell cycle | 0.001462183 | 0.003643474 | 0.001185895 | 3 |
| hsa05168 | Herpes simplex virus 1 infection | 0.001535187 | 0.003763684 | 0.001225022 | 5 |
| hsa04926 | Relaxin signaling pathway | 0.001564764 | 0.003775303 | 0.001228804 | 3 |
| hsa04010 | MAPK signaling pathway | 0.001695515 | 0.004026849 | 0.001310678 | 4 |
| hsa05016 | Huntington disease | 0.001965091 | 0.00459529 | 0.001495697 | 4 |
| hsa05216 | Thyroid cancer | 0.002057071 | 0.00467628 | 0.001522058 | 2 |
| hsa05206 | MicroRNAs in cancer | 0.002061255 | 0.00467628 | 0.001522058 | 4 |
| hsa05219 | Bladder cancer | 0.002521913 | 0.005637216 | 0.001834828 | 2 |
| hsa04934 | Cushing syndrome | 0.00264717 | 0.005831447 | 0.001898047 | 3 |
| hsa04217 | Necroptosis | 0.00284611 | 0.006180125 | 0.002011537 | 3 |
| hsa05010 | Alzheimer disease | 0.004487443 | 0.00960692 | 0.003126906 | 4 |
| hsa05416 | Viral myocarditis | 0.005334139 | 0.011260959 | 0.003665271 | 2 |
| hsa05205 | Proteoglycans in cancer | 0.005819102 | 0.012050703 | 0.00392232 | 3 |
| hsa05217 | Basal cell carcinoma | 0.00586679 | 0.012050703 | 0.00392232 | 2 |
| hsa05207 | Chemical carcinogenesis - receptor activation | 0.006388739 | 0.012947844 | 0.004214326 | 3 |
| hsa05031 | Amphetamine addiction | 0.00700231 | 0.013822743 | 0.004499092 | 2 |
| hsa05211 | Renal cell carcinoma | 0.00700231 | 0.013822743 | 0.004499092 | 2 |
| hsa05120 | Epithelial cell signaling in Helicobacter pylori infection | 0.007200562 | 0.013854246 | 0.004509346 | 2 |
| hsa05230 | Central carbon metabolism in cancer | 0.007200562 | 0.013854246 | 0.004509346 | 2 |
| hsa04137 | Mitophagy - animal | 0.007604699 | 0.014270546 | 0.004644845 | 2 |
| hsa05218 | Melanoma | 0.007604699 | 0.014270546 | 0.004644845 | 2 |
| hsa04014 | Ras signaling pathway | 0.008197727 | 0.014892119 | 0.004847158 | 3 |
| hsa05171 | Coronavirus disease - COVID-19 | 0.008197727 | 0.014892119 | 0.004847158 | 3 |
| hsa05214 | Glioma | 0.008229855 | 0.014892119 | 0.004847158 | 2 |
| hsa05133 | Pertussis | 0.008443259 | 0.015098534 | 0.004914343 | 2 |
| hsa04020 | Calcium signaling pathway | 0.008998418 | 0.01590418 | 0.005176568 | 3 |
| hsa04662 | B cell receptor signaling pathway | 0.009775768 | 0.017079502 | 0.005559118 | 2 |
| hsa05235 | PD-L1 expression and PD-1 checkpoint pathway in cancer | 0.011441384 | 0.019762391 | 0.006432357 | 2 |
| hsa05012 | Parkinson disease | 0.011914612 | 0.020348551 | 0.006623143 | 3 |
| hsa04658 | Th1 and Th2 cell differentiation | 0.012191138 | 0.020589477 | 0.006701561 | 2 |
| hsa04912 | GnRH signaling pathway | 0.012445783 | 0.02078856 | 0.00676636 | 2 |
| hsa05231 | Choline metabolism in cancer | 0.013754085 | 0.02272414 | 0.007396361 | 2 |
| hsa04640 | Hematopoietic cell lineage | 0.014022702 | 0.022918826 | 0.007459729 | 2 |
| hsa05146 | Amoebiasis | 0.01484232 | 0.024000348 | 0.007811748 | 2 |
| hsa04064 | NF-kappa B signaling pathway | 0.015400121 | 0.024640194 | 0.008020008 | 2 |
| hsa04066 | HIF-1 signaling pathway | 0.016834007 | 0.026653845 | 0.00867542 | 2 |
| hsa04935 | Growth hormone synthesis, secretion and action | 0.019867139 | 0.031132011 | 0.010132995 | 2 |
| hsa04380 | Osteoclast differentiation | 0.022780025 | 0.035332284 | 0.01150012 | 2 |
| hsa04068 | FoxO signaling pathway | 0.023788495 | 0.03652375 | 0.011887924 | 2 |
| hsa04915 | Estrogen signaling pathway | 0.026212816 | 0.039843481 | 0.012968446 | 2 |
| hsa05014 | Amyotrophic lateral sclerosis | 0.027638333 | 0.041594323 | 0.013538319 | 3 |
| hsa04921 | Oxytocin signaling pathway | 0.032116541 | 0.047859944 | 0.015577683 | 2 |
| hsa04310 | Wnt signaling pathway | 0.036860793 | 0.05439651 | 0.017705235 | 2 |
| hsa04360 | Axon guidance | 0.043584914 | 0.063701028 | 0.020733714 | 2 |
| hsa04062 | Chemokine signaling pathway | 0.048007335 | 0.069496332 | 0.022619997 | 2 |
| hsa01523 | Antifolate resistance | 0.055988882 | 0.079334317 | 0.025822112 | 1 |
| hsa05310 | Asthma | 0.055988882 | 0.079334317 | 0.025822112 | 1 |
| hsa04015 | Rap1 signaling pathway | 0.05636912 | 0.079334317 | 0.025822112 | 2 |
| hsa04810 | Regulation of actin cytoskeleton | 0.060243046 | 0.084008652 | 0.027343536 | 2 |
| hsa05143 | African trypanosomiasis | 0.066481676 | 0.091865588 | 0.029900849 | 1 |
| hsa05330 | Allograft rejection | 0.068219855 | 0.09341818 | 0.030406194 | 1 |
| hsa05332 | Graft-versus-host disease | 0.075142412 | 0.101978987 | 0.033192607 | 1 |
| hsa04940 | Type I diabetes mellitus | 0.076865535 | 0.103394348 | 0.033653285 | 1 |
| hsa04672 | Intestinal immune network for IgA production | 0.087141511 | 0.115178345 | 0.037488796 | 1 |
| hsa05030 | Cocaine addiction | 0.087141511 | 0.115178345 | 0.037488796 | 1 |
| hsa05144 | Malaria | 0.088843758 | 0.116415959 | 0.037891621 | 1 |
| hsa05320 | Autoimmune thyroid disease | 0.093932741 | 0.122032279 | 0.039719648 | 1 |
| hsa04340 | Hedgehog signaling pathway | 0.098995186 | 0.127519223 | 0.041505564 | 1 |
| hsa04060 | Cytokine-cytokine receptor interaction | 0.101807954 | 0.130040411 | 0.042326173 | 2 |
| hsa04330 | Notch signaling pathway | 0.104031222 | 0.131772881 | 0.042890065 | 1 |
| hsa04213 | Longevity regulating pathway - multiple species | 0.109040976 | 0.136977094 | 0.044583957 | 1 |
| hsa04623 | Cytosolic DNA-sensing pathway | 0.110705076 | 0.137927636 | 0.044893344 | 1 |
| hsa04664 | Fc epsilon RI signaling pathway | 0.118982149 | 0.147034851 | 0.047857604 | 1 |
| hsa04622 | RIG-I-like receptor signaling pathway | 0.122272801 | 0.148683726 | 0.048394287 | 1 |
| hsa04917 | Prolactin signaling pathway | 0.122272801 | 0.148683726 | 0.048394287 | 1 |
| hsa04520 | Adherens junction | 0.123913821 | 0.14948334 | 0.04865455 | 1 |
| hsa05100 | Bacterial invasion of epithelial cells | 0.133699965 | 0.160018856 | 0.0520837 | 1 |
| hsa04540 | Gap junction | 0.151376759 | 0.179759901 | 0.058509109 | 1 |
| hsa04211 | Longevity regulating pathway | 0.152966934 | 0.180240108 | 0.058665409 | 1 |
| hsa04750 | Inflammatory mediator regulation of TRP channels | 0.167153883 | 0.195441464 | 0.063613219 | 1 |
| hsa04916 | Melanogenesis | 0.171833367 | 0.199379174 | 0.064894884 | 1 |
| hsa04914 | Progesterone-mediated oocyte maturation | 0.173387734 | 0.199658603 | 0.064985833 | 1 |
| hsa04928 | Parathyroid hormone synthesis, secretion and action | 0.179578014 | 0.205232016 | 0.066799894 | 1 |
| hsa04931 | Insulin resistance | 0.182656903 | 0.207192904 | 0.067438134 | 1 |
| hsa04670 | Leukocyte transendothelial migration | 0.191828988 | 0.215985231 | 0.070299902 | 1 |
| hsa04071 | Sphingolipid signaling pathway | 0.199398915 | 0.22285761 | 0.072536757 | 1 |
| hsa04114 | Oocyte meiosis | 0.217297585 | 0.240966433 | 0.078430903 | 1 |
| hsa04728 | Dopaminergic synapse | 0.218772156 | 0.240966433 | 0.078430903 | 1 |
| hsa04910 | Insulin signaling pathway | 0.2261062 | 0.247252823 | 0.080477027 | 1 |
| hsa04140 | Autophagy - animal | 0.231927113 | 0.25180658 | 0.081959205 | 1 |
| hsa04550 | Signaling pathways regulating pluripotency of stem cells | 0.234822221 | 0.253141685 | 0.082393762 | 1 |
| hsa04072 | Phospholipase D signaling pathway | 0.242015493 | 0.259058838 | 0.084319705 | 1 |
| hsa04150 | mTOR signaling pathway | 0.251980092 | 0.267105493 | 0.086938769 | 1 |
| hsa04218 | Cellular senescence | 0.253393584 | 0.267105493 | 0.086938769 | 1 |
| hsa04390 | Hippo signaling pathway | 0.254804582 | 0.267105493 | 0.086938769 | 1 |
| hsa04530 | Tight junction | 0.271543574 | 0.282702898 | 0.092015486 | 1 |
| hsa04141 | Protein processing in endoplasmic reticulum | 0.274299049 | 0.283628948 | 0.092316901 | 1 |
| hsa05034 | Alcoholism | 0.29599522 | 0.303995091 | 0.098945771 | 1 |
| hsa04613 | Neutrophil extracellular trap formation | 0.299995238 | 0.30603541 | 0.099609863 | 1 |
| hsa05415 | Diabetic cardiomyopathy | 0.317084775 | 0.321312572 | 0.104582347 | 1 |
| hsa04024 | cAMP signaling pathway | 0.337583495 | 0.339819147 | 0.110605955 | 1 |
| hsa04144 | Endocytosis | 0.378061571 | 0.378061571 | 0.123053281 | 1 |

**The information of KEGG enrichment analysis for module 3.**

| **ID** | **Description** | **pvalue** | **p.adjust** | **qvalue** | **Count** |
| --- | --- | --- | --- | --- | --- |
| hsa05161 | Hepatitis B | 1.15E-08 | 1.81E-06 | 8.37E-07 | 6 |
| hsa05417 | Lipid and atherosclerosis | 2.86E-06 | 0.000224213 | 0.000103726 | 5 |
| hsa05167 | Kaposi sarcoma-associated herpesvirus infection | 6.00E-05 | 0.001912337 | 0.000884688 | 4 |
| hsa05169 | Epstein-Barr virus infection | 7.03E-05 | 0.001912337 | 0.000884688 | 4 |
| hsa05120 | Epithelial cell signaling in Helicobacter pylori infection | 7.12E-05 | 0.001912337 | 0.000884688 | 3 |
| hsa05203 | Viral carcinogenesis | 7.31E-05 | 0.001912337 | 0.000884688 | 4 |
| hsa05166 | Human T-cell leukemia virus 1 infection | 0.000101634 | 0.002101538 | 0.000972217 | 4 |
| hsa05163 | Human cytomegalovirus infection | 0.000107085 | 0.002101538 | 0.000972217 | 4 |
| hsa04012 | ErbB signaling pathway | 0.000127239 | 0.002219613 | 0.001026841 | 3 |
| hsa04912 | GnRH signaling pathway | 0.000166299 | 0.002491217 | 0.001152491 | 3 |
| hsa01522 | Endocrine resistance | 0.000194281 | 0.002491217 | 0.001152491 | 3 |
| hsa04750 | Inflammatory mediator regulation of TRP channels | 0.000194281 | 0.002491217 | 0.001152491 | 3 |
| hsa04933 | AGE-RAGE signaling pathway in diabetic complications | 0.000206279 | 0.002491217 | 0.001152491 | 3 |
| hsa04926 | Relaxin signaling pathway | 0.000437493 | 0.004906176 | 0.002269703 | 3 |
| hsa05165 | Human papillomavirus infection | 0.000474389 | 0.004965276 | 0.002297043 | 4 |
| hsa04210 | Apoptosis | 0.000510926 | 0.005013462 | 0.002319336 | 3 |
| hsa04215 | Apoptosis - multiple species | 0.000668292 | 0.006171877 | 0.002855243 | 2 |
| hsa04932 | Non-alcoholic fatty liver disease | 0.000749076 | 0.006533609 | 0.003022588 | 3 |
| hsa05225 | Hepatocellular carcinoma | 0.000947151 | 0.007826456 | 0.003620687 | 3 |
| hsa05219 | Bladder cancer | 0.001098299 | 0.008621646 | 0.003988559 | 2 |
| hsa05152 | Tuberculosis | 0.00115722 | 0.008651598 | 0.004002415 | 3 |
| hsa05130 | Pathogenic Escherichia coli infection | 0.001502401 | 0.010582515 | 0.004895699 | 3 |
| hsa04510 | Focal adhesion | 0.001592093 | 0.010582515 | 0.004895699 | 3 |
| hsa05415 | Diabetic cardiomyopathy | 0.001638184 | 0.010582515 | 0.004895699 | 3 |
| hsa05205 | Proteoglycans in cancer | 0.001685114 | 0.010582515 | 0.004895699 | 3 |
| hsa05170 | Human immunodeficiency virus 1 infection | 0.001856056 | 0.010792623 | 0.0049929 | 3 |
| hsa05207 | Chemical carcinogenesis - receptor activation | 0.001856056 | 0.010792623 | 0.0049929 | 3 |
| hsa04370 | VEGF signaling pathway | 0.002264643 | 0.012698176 | 0.005874449 | 2 |
| hsa04664 | Fc epsilon RI signaling pathway | 0.002997262 | 0.016226554 | 0.007506753 | 2 |
| hsa04917 | Prolactin signaling pathway | 0.003173328 | 0.016456416 | 0.007613092 | 2 |
| hsa04137 | Mitophagy - animal | 0.003354174 | 0.016456416 | 0.007613092 | 2 |
| hsa05223 | Non-small cell lung cancer | 0.003354174 | 0.016456416 | 0.007613092 | 2 |
| hsa05214 | Glioma | 0.003634361 | 0.016732275 | 0.00774071 | 2 |
| hsa05133 | Pertussis | 0.003730125 | 0.016732275 | 0.00774071 | 2 |
| hsa05212 | Pancreatic cancer | 0.003730125 | 0.016732275 | 0.00774071 | 2 |
| hsa01521 | EGFR tyrosine kinase inhibitor resistance | 0.004024487 | 0.017551235 | 0.008119579 | 2 |
| hsa04010 | MAPK signaling pathway | 0.00470889 | 0.019634337 | 0.009083267 | 3 |
| hsa05210 | Colorectal cancer | 0.00475226 | 0.019634337 | 0.009083267 | 2 |
| hsa04540 | Gap junction | 0.004970631 | 0.019945062 | 0.009227015 | 2 |
| hsa04727 | GABAergic synapse | 0.005081544 | 0.019945062 | 0.009227015 | 2 |
| hsa05016 | Huntington disease | 0.005269603 | 0.019954403 | 0.009231337 | 3 |
| hsa05222 | Small cell lung cancer | 0.005421163 | 0.019954403 | 0.009231337 | 2 |
| hsa05206 | MicroRNAs in cancer | 0.005465219 | 0.019954403 | 0.009231337 | 3 |
| hsa04657 | IL-17 signaling pathway | 0.005653285 | 0.020171949 | 0.009331978 | 2 |
| hsa05231 | Choline metabolism in cancer | 0.006131143 | 0.021390878 | 0.00989588 | 2 |
| hsa04914 | Progesterone-mediated oocyte maturation | 0.006627027 | 0.022137091 | 0.010241095 | 2 |
| hsa05146 | Amoebiasis | 0.006627027 | 0.022137091 | 0.010241095 | 2 |
| hsa04625 | C-type lectin receptor signaling pathway | 0.006881681 | 0.022508831 | 0.01041307 | 2 |
| hsa04668 | TNF signaling pathway | 0.007944598 | 0.024946038 | 0.011540574 | 2 |
| hsa05145 | Toxoplasmosis | 0.007944598 | 0.024946038 | 0.011540574 | 2 |
| hsa04726 | Serotonergic synapse | 0.008361298 | 0.025739682 | 0.011907731 | 2 |
| hsa04071 | Sphingolipid signaling pathway | 0.008932096 | 0.026330232 | 0.012180932 | 2 |
| hsa04935 | Growth hormone synthesis, secretion and action | 0.008932096 | 0.026330232 | 0.012180932 | 2 |
| hsa04152 | AMPK signaling pathway | 0.009077494 | 0.026330232 | 0.012180932 | 2 |
| hsa04919 | Thyroid hormone signaling pathway | 0.009223967 | 0.026330232 | 0.012180932 | 2 |
| hsa04110 | Cell cycle | 0.00997237 | 0.02795825 | 0.012934088 | 2 |
| hsa04380 | Osteoclast differentiation | 0.010279173 | 0.028312809 | 0.013098115 | 2 |
| hsa04650 | Natural killer cell mediated cytotoxicity | 0.010747293 | 0.029019549 | 0.013425068 | 2 |
| hsa04728 | Dopaminergic synapse | 0.010905435 | 0.029019549 | 0.013425068 | 2 |
| hsa05135 | Yersinia infection | 0.011711806 | 0.030492488 | 0.014106481 | 2 |
| hsa05162 | Measles | 0.012041619 | 0.030492488 | 0.014106481 | 2 |
| hsa05418 | Fluid shear stress and atherosclerosis | 0.012041619 | 0.030492488 | 0.014106481 | 2 |
| hsa05017 | Spinocerebellar ataxia | 0.012713602 | 0.031683104 | 0.014657286 | 2 |
| hsa04723 | Retrograde endocannabinoid signaling | 0.013576562 | 0.033216841 | 0.015366825 | 2 |
| hsa05226 | Gastric cancer | 0.013752195 | 0.033216841 | 0.015366825 | 2 |
| hsa04921 | Oxytocin signaling pathway | 0.014645448 | 0.034838413 | 0.016117 | 2 |
| hsa04218 | Cellular senescence | 0.015009747 | 0.035078849 | 0.016228231 | 2 |
| hsa05160 | Hepatitis C | 0.015193387 | 0.035078849 | 0.016228231 | 2 |
| hsa04217 | Necroptosis | 0.015563638 | 0.035412915 | 0.016382777 | 2 |
| hsa04310 | Wnt signaling pathway | 0.016890475 | 0.037882922 | 0.017525455 | 2 |
| hsa04530 | Tight junction | 0.017473729 | 0.038639092 | 0.017875275 | 2 |
| hsa05022 | Pathways of neurodegeneration - multiple diseases | 0.017784156 | 0.038779341 | 0.017940158 | 3 |
| hsa05164 | Influenza A | 0.018065665 | 0.038853554 | 0.01797449 | 2 |
| hsa04360 | Axon guidance | 0.020100732 | 0.042646149 | 0.019729026 | 2 |
| hsa04613 | Neutrophil extracellular trap formation | 0.021796323 | 0.04562697 | 0.021108018 | 2 |
| hsa05202 | Transcriptional misregulation in cancer | 0.022229459 | 0.045921383 | 0.02124422 | 2 |
| hsa04015 | Rap1 signaling pathway | 0.02629044 | 0.053605183 | 0.024798912 | 2 |
| hsa05208 | Chemical carcinogenesis - reactive oxygen species | 0.029400977 | 0.05917889 | 0.027377428 | 2 |
| hsa04014 | Ras signaling pathway | 0.031638991 | 0.06209152 | 0.028724873 | 2 |
| hsa05171 | Coronavirus disease - COVID-19 | 0.031638991 | 0.06209152 | 0.028724873 | 2 |
| hsa05131 | Shigellosis | 0.035518323 | 0.068844157 | 0.031848789 | 2 |
| hsa05132 | Salmonella infection | 0.036049378 | 0.069021369 | 0.031930771 | 2 |
| hsa03410 | Base excision repair | 0.040057722 | 0.075771836 | 0.035053682 | 1 |
| hsa05012 | Parkinson disease | 0.040690978 | 0.076053375 | 0.035183928 | 2 |
| hsa05020 | Prion disease | 0.042667215 | 0.078808856 | 0.036458673 | 2 |
| hsa04960 | Aldosterone-regulated sodium reabsorption | 0.044813729 | 0.079951766 | 0.036987408 | 1 |
| hsa05143 | African trypanosomiasis | 0.044813729 | 0.079951766 | 0.036987408 | 1 |
| hsa05216 | Thyroid cancer | 0.044813729 | 0.079951766 | 0.036987408 | 1 |
| hsa04930 | Type II diabetes mellitus | 0.055437294 | 0.097793879 | 0.045241553 | 1 |
| hsa00270 | Cysteine and methionine metabolism | 0.060124651 | 0.10373154 | 0.047988443 | 1 |
| hsa05110 | Vibrio cholerae infection | 0.060124651 | 0.10373154 | 0.047988443 | 1 |
| hsa04961 | Endocrine and other factor-regulated calcium reabsorption | 0.063626418 | 0.108579866 | 0.050231383 | 1 |
| hsa05134 | Legionellosis | 0.068277175 | 0.115263618 | 0.05332343 | 1 |
| hsa04730 | Long-term depression | 0.071751589 | 0.118578941 | 0.05485717 | 1 |
| hsa05416 | Viral myocarditis | 0.071751589 | 0.118578941 | 0.05485717 | 1 |
| hsa04929 | GnRH secretion | 0.076366 | 0.12489023 | 0.057776908 | 1 |
| hsa05010 | Alzheimer disease | 0.078533073 | 0.126572528 | 0.058555175 | 2 |
| hsa04720 | Long-term potentiation | 0.07981325 | 0.126572528 | 0.058555175 | 1 |
| hsa05221 | Acute myeloid leukemia | 0.07981325 | 0.126572528 | 0.058555175 | 1 |
| hsa04920 | Adipocytokine signaling pathway | 0.082104979 | 0.127628531 | 0.059043705 | 1 |
| hsa05031 | Amphetamine addiction | 0.082104979 | 0.127628531 | 0.059043705 | 1 |
| hsa04622 | RIG-I-like receptor signaling pathway | 0.083248916 | 0.128138037 | 0.059279414 | 1 |
| hsa04520 | Adherens junction | 0.084391569 | 0.128374208 | 0.059388672 | 1 |
| hsa05218 | Melanoma | 0.085532941 | 0.128374208 | 0.059388672 | 1 |
| hsa01524 | Platinum drug resistance | 0.086673032 | 0.128374208 | 0.059388672 | 1 |
| hsa04115 | p53 signaling pathway | 0.086673032 | 0.128374208 | 0.059388672 | 1 |
| hsa04918 | Thyroid hormone synthesis | 0.088949378 | 0.128576769 | 0.05948238 | 1 |
| hsa03320 | PPAR signaling pathway | 0.090085634 | 0.128576769 | 0.05948238 | 1 |
| hsa04971 | Gastric acid secretion | 0.090085634 | 0.128576769 | 0.05948238 | 1 |
| hsa05220 | Chronic myeloid leukemia | 0.090085634 | 0.128576769 | 0.05948238 | 1 |
| hsa05100 | Bacterial invasion of epithelial cells | 0.091220615 | 0.129023753 | 0.059689165 | 1 |
| hsa04911 | Insulin secretion | 0.101378254 | 0.142110588 | 0.065743417 | 1 |
| hsa04211 | Longevity regulating pathway | 0.104741356 | 0.145525601 | 0.067323275 | 1 |
| hsa05032 | Morphine addiction | 0.106977126 | 0.147328148 | 0.068157172 | 1 |
| hsa04658 | Th1 and Th2 cell differentiation | 0.108093126 | 0.147570615 | 0.068269343 | 1 |
| hsa04970 | Salivary secretion | 0.10920787 | 0.147807203 | 0.068378793 | 1 |
| hsa04070 | Phosphatidylinositol signaling system | 0.113654319 | 0.148697734 | 0.068790772 | 1 |
| hsa04666 | Fc gamma R-mediated phagocytosis | 0.113654319 | 0.148697734 | 0.068790772 | 1 |
| hsa04713 | Circadian entrainment | 0.113654319 | 0.148697734 | 0.068790772 | 1 |
| hsa05215 | Prostate cancer | 0.113654319 | 0.148697734 | 0.068790772 | 1 |
| hsa04925 | Aldosterone synthesis and secretion | 0.114762805 | 0.148907111 | 0.068887634 | 1 |
| hsa04916 | Melanogenesis | 0.118080782 | 0.149845621 | 0.069321809 | 1 |
| hsa04972 | Pancreatic secretion | 0.119184285 | 0.149845621 | 0.069321809 | 1 |
| hsa05142 | Chagas disease | 0.119184285 | 0.149845621 | 0.069321809 | 1 |
| hsa04064 | NF-kappa B signaling pathway | 0.121387562 | 0.149845621 | 0.069321809 | 1 |
| hsa04620 | Toll-like receptor signaling pathway | 0.121387562 | 0.149845621 | 0.069321809 | 1 |
| hsa04660 | T cell receptor signaling pathway | 0.121387562 | 0.149845621 | 0.069321809 | 1 |
| hsa05168 | Herpes simplex virus 1 infection | 0.12216713 | 0.149845621 | 0.069321809 | 2 |
| hsa04928 | Parathyroid hormone synthesis, secretion and action | 0.123585879 | 0.150410721 | 0.069583237 | 1 |
| hsa04659 | Th17 cell differentiation | 0.125779244 | 0.150743063 | 0.069736985 | 1 |
| hsa04931 | Insulin resistance | 0.125779244 | 0.150743063 | 0.069736985 | 1 |
| hsa04066 | HIF-1 signaling pathway | 0.126874072 | 0.150903253 | 0.069811093 | 1 |
| hsa04725 | Cholinergic synapse | 0.131241058 | 0.153894573 | 0.071194942 | 1 |
| hsa04670 | Leukocyte transendothelial migration | 0.132329729 | 0.153894573 | 0.071194942 | 1 |
| hsa04724 | Glutamatergic synapse | 0.132329729 | 0.153894573 | 0.071194942 | 1 |
| hsa04722 | Neurotrophin signaling pathway | 0.137754685 | 0.159025629 | 0.073568679 | 1 |
| hsa04611 | Platelet activation | 0.143149096 | 0.164046775 | 0.075891569 | 1 |
| hsa04068 | FoxO signaling pathway | 0.150650246 | 0.171391946 | 0.079289603 | 1 |
| hsa04270 | Vascular smooth muscle contraction | 0.152782548 | 0.172567339 | 0.079833365 | 1 |
| hsa04910 | Insulin signaling pathway | 0.157032706 | 0.176031788 | 0.081436093 | 1 |
| hsa04915 | Estrogen signaling pathway | 0.158092242 | 0.176031788 | 0.081436093 | 1 |
| hsa04140 | Autophagy - animal | 0.161263665 | 0.178298559 | 0.082484751 | 1 |
| hsa05224 | Breast cancer | 0.167574273 | 0.183844685 | 0.085050508 | 1 |
| hsa04072 | Phospholipase D signaling pathway | 0.168621877 | 0.183844685 | 0.085050508 | 1 |
| hsa04261 | Adrenergic signaling in cardiomyocytes | 0.170713525 | 0.184841541 | 0.085511675 | 1 |
| hsa04150 | mTOR signaling pathway | 0.17592194 | 0.187889419 | 0.086921689 | 1 |
| hsa04934 | Cushing syndrome | 0.17592194 | 0.187889419 | 0.086921689 | 1 |
| hsa04141 | Protein processing in endoplasmic reticulum | 0.192391632 | 0.204091123 | 0.094416946 | 1 |
| hsa04621 | NOD-like receptor signaling pathway | 0.205554468 | 0.21659095 | 0.100199635 | 1 |
| hsa04062 | Chemokine signaling pathway | 0.213558387 | 0.223524445 | 0.103407219 | 1 |
| hsa04810 | Regulation of actin cytoskeleton | 0.239072432 | 0.247934698 | 0.114699927 | 1 |
| hsa04024 | cAMP signaling pathway | 0.240038689 | 0.247934698 | 0.114699927 | 1 |
| hsa04714 | Thermogenesis | 0.252499937 | 0.259101242 | 0.119865811 | 1 |
| hsa04020 | Calcium signaling pathway | 0.260076687 | 0.265143116 | 0.122660912 | 1 |
| hsa04144 | Endocytosis | 0.271312259 | 0.274813063 | 0.127134437 | 1 |
| hsa04151 | PI3K-Akt signaling pathway | 0.36079154 | 0.363104307 | 0.167979867 | 1 |
| hsa05014 | Amyotrophic lateral sclerosis | 0.369824399 | 0.369824399 | 0.171088726 | 1 |
